# Surface‐Functionalized LLZO‐Incorporated Multilayer Composite Solid Electrolytes for Dendrite Suppression and Efficient Ionic Conduction in Lithium–Metal Batteries

**DOI:** 10.1002/adma.73879

**Published:** 2026-06-25

**Authors:** Fazal Ur Rehman, Minhong Woo, Hyesoo Choi, Jihwan Kim, Yujin Kim, Sanghee Park, Serim Ahn, Jinsub Lim, Minyoung Kim, Mincheol Chang

**Affiliations:** ^1^ Department of Polymer Engineering Chonnam National University Gwangju South Korea; ^2^ Korea Institute of Industrial Technology Gwangju South Korea; ^3^ School of Polymer Science and Engineering Chonnam National University Gwangju South Korea

**Keywords:** dendrite suppression, ionic transport enhancement, lithium metal battery, surface modified LLZO, tri‐layered electrolytes

## Abstract

The development of solid polymer electrolytes is central to safe, high‐energy lithium‐metal batteries (LMBs); however, persistent challenges including dendritic‐lithium‐growth, interfacial instability, and low ionic‐conductivity impede their commercialization. Herein, we report a tri‐layered composite solid electrolyte (CSE) that couples interfacial engineering with mechanical‐reinforcement to address them. The outer layers consist of PEO/LiTFSI, while inner layer comprises a PEO/LiTFSI matrix reinforced with polydopamine‐coated Li_7_La_3_Zr_2_O_12_ (PDA@LLZO, 10–40 wt%) and poly(ethylene glycol)‐block‐poly(propylene glycol)‐block‐poly(ethylene glycol) (PPP). The PDA coating promotes strong hydrogen‐bonding with PEO‐matrix, leading to uniform dispersion and reduced interfacial resistance. LLZO enables percolated Li^+^‐transport channels and disrupts PEO crystallinity, advancing segmental dynamics. Simultaneously, PPP elastomers offer mechanical compliance, redistribute localized stress, and dissipate dendritic intrusions to suppress crack propagation. The optimized CSE‐30 (30 wt% PDA@LLZO) exhibits an ionic‐conductivity of 5.60×10^−3^ S cm^−^^1^ at 60°C and 8.04 × 10^−5^ S cm^−^^1^ at 25°C, nearly four‐times higher than PEO, with a Li^+^‐transference number of 0.81 and anodic stability up to 5.6 V vs. Li/Li^+^. In Li/LFP full cells, CSE‐30 delivered a capacity of 133.6 mAh g^−^^1^ at 0.5C with 80% retention after 1000 cycles and Li/Li symmetric cells sustained over 1000 h cycling without short‐circuiting. This multifunctional CSE design advances next‐generation solid‐state LMBs by integrating efficient Li^+^‐transport and mechanical resilience.

## Graphical abstract

A soft–hard tri‐layer composite electrolyte that couples fast Li^+^ transport with reinforced interfacial stability to enable high‐conductivity, mechanically robust, dendrite‐free lithium‐metal batteries.

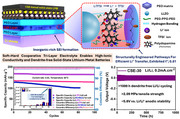

## 1 Introduction

The exploration of advanced rechargeable batteries has sparked significant interest in lithium‐metal batteries (LMBs), recognized for their remarkable theoretical specific capacity of about 3860 mAh g^−^^1^ and lower redox potential (‐3.04 V vs SHE) among all anode materials [1]. These characteristics position the lithium (Li) metal as a highly promising candidate for driving high‐energy‐density devices, ranging from portable electronics to enduring electric vehicles. However, the practical application of LMBs remains hampered due to persistent interfacial issues including dendrite formation, electrolyte breakdown, and uneven solid electrolyte interphases (SEIs) which detrimentally compromise the Coulombic efficiency, cycling stability, and operational safety [2]. Shifting from traditional flammable liquid electrolytes (LEs) to solid‐state electrolytes (SSEs) emerges as a promising strategy to address these challenges. Solid polymer electrolytes (SPEs), especially those composed of poly(ethylene oxide) (PEO) matrix, have gained attention owing to their advantageous electrochemical stability, mechanical flexibility, and facile manufacturing [3]. Yet, their practical application is restricted by inherently low ionic conductivity ranging from 10^−^^7^ to 10^−^^6^ S cm^−^^1^ at room temperature and higher semi‐crystallinity, which impede the Li^+^‐ion mobility and intensify interfacial mismatch with Li‐metal [4].

In order to overcome these challenges, researchers have integrated garnet‐type Li_7_La_3_Zr_2_O_12_ (LLZO) additives into PEO matrix to decrease crystallinity and improve Li^+^‐ion mobility [5, 6]. Although LLZO enhances ionic conductivity and mechanical stability, inadequate polymer‐ceramic compatibility often results in phase separation and increased interfacial resistance [7]. Surface functionalization of LLZO with polydopamine (PDA), a catecholamine‐based adhesive, efficiently improves interfacial adhesion and chemical compatibility with PEO [8, 9, 10]. The PDA layer forms hydrogen bonds with polymer chains, passivates reactive sites on the surface, and creates a flexible interface which promotes Li^+^‐ion transport [11, 12]. Additionally, using the multi‐layer SPE architectures have demonstrated potential in mitigating dendritic formation by spatially separating mechanical and ionic functionalities [13]. Despite these innovations, the combinatorial use of chemically tailored ceramic additives and flexible polymers within a multilayer structure remains insufficiently studied.

In addition to intrinsic ionic and mechanical factors, a critical advantage of multi‐layer SSE designs lies in reducing the interfacial impedance at cathode–electrolyte interface (CEI), exceptionally between ceramic‐rich phases and LFP electrodes [14]. Direct interaction between stiff ceramic electrolytes, such as LLZO, and cathode often results in higher interfacial resistance because of inadequate surface wetting, mechanical modulus difference, and interphase void formation during the cycling [15, 16, 17]. In order to alleviate these challenges, latest research have demonstrated that introducing the gradient, ion‐conducting inter‐layers as well as polymer‐rich buffer coatings can remarkedly suppress interfacial resistance by enhancing the Li^+^ flux continuity, mechanical strength, and electrochemical cycling stability [18, 19]. For instance, Hu et al. [20] constructed a tri‐layer SSE that improved cathode–electrolyte compatibility and controlled interfacial degradation using architectural layering strategies. A recent study by Liang et al. [21] revealed that inserting an ionic‐conductor inter‐layer drastically mitigates the interfacial potential drop at CEI, enhancing overall interfacial dynamics in SSBs. Also, Liu et al. [22] designed a gradient tri‐layer SSE, demonstrating improved interfacial compatibility at both electrodes by modifying the mechanical and ionic properties across the layers. Wang et al. [23] also emphasize that interfacial engineering by soft ion‐conducting inter‐layers, polymer–ceramic gradient hybrids, or phase boundary modifiers, is a key approach to alleviate interfacial impedance and enable long‐term cathode compatibility in composite SSBs. These findings collectively focus that interfacial engineering, particularly using multi‐layer and soft‐buffer strategies, characterizes a key design route for stabilizing solid–solid boundaries in composite SSEs.

In response to these challenges, we developed a tri‐layer composite solid electrolyte (CSE) that integrates interfacial chemistry and mechanical design to simultaneously enhance ion transport and suppress dendrite formation in PEO‐based systems. The tri‐layer architecture consists of soft PEO/LiTFSI outer layers that ensure intimate electrode contact and efficient Li^+^ transport, while the central interlayer comprises a PEO/LiTFSI matrix reinforced with polydopamine‐coated Li_7_La_3_Zr_2_O_12_ (PDA@LLZO) as a functional ceramic additive and poly(ethylene glycol)‐block‐poly(propylene glycol)‐block‐poly(ethylene glycol) (PPP) as a ductile polymeric component, providing mechanical strength and structural integrity. To the best of our knowledge, this is the first report to combine PDA@LLZO and PPP within a multilayer PEO framework, enabling a synergistic improvement in both electrochemical and structural stability. Mechanistically, the tri‐layer configuration decouples ionic and mechanical functions across spatial domains. The soft outer layers provide conformal Li^+^ transport channels and intimate electrode contact, while the PDA@LLZO0‐PPP interlayer offers mechanical reinforcement and continuous ion pathways through hydrogen‐bond coupling and percolated conduction networks. The PPP phase introduces a soft–hard gradient that redistributes stress and deflects dendritic intrusion, while the separation of the ceramic‐rich layer from the cathode minimizes interfacial impedance. The optimized CSE‐30 exhibits an ionic conductivity of 5.6 × 10^−^^3^ S cm^−^^1^ at 60°C and a lithium transference number of 0.81. In Li|Li symmetric cells, it delivers over 1000 h of dendrite‐free cycling at 0.2 mA cm^−^^2^, and in LFP|Li full cells, it achieves 133.6 mAh g^−^^1^ at 0.5C with 80% capacity retention and 99% Coulombic efficiency after 1000 cycles. These results highlight a co‐engineered electrolyte platform where chemical functionalization and architectural layering converge to achieve stable, high‐performance solid‐state LMBs.

## 2 Materials and Methods

All experimental details, including used materials (Figure S1), electrolytes preparation, characterization techniques, electrochemical measurements, and statistical analysis are described in the Supporting Information.

## 3 Results and Discussion

The explicit incorporation of pristine LLZO into PEO‐based SPEs encounters various challenges that hamper its efficiency. Pristine LLZO, characterized as a rigid ceramic matter, demonstrates poor compatibility with flexible and soft PEO matrix, resulting in inadequate interfacial adhesion [6]. This incompatibility leads to elevated interfacial resistance and irregular ion transport pathways, ultimately compromising the overall ionic conductivity of system. Moreover, LLZO surface is often contaminated with impurities like Li_2_CO_3_ and other air‐induced reaction by‐products, which not only diminishes intrinsic ionic conductivity of LLZO but also induces chemical instability when integrated with PEO matrix. Additionally, pristine LLZO particles have tendency to agglomerate due to robust ceramic particle–particle interactions, causing uneven dispersion within the PEO matrix and thereby limiting the advantages of incorporating ceramic additives. To address these challenges, PDA‐coated LLZO has emerged as a promising alternate solution. PDA, a bio‐inspired polymer with remarkable surface engineering properties, substantially improves the interfacial compatibility between PEO chains and LLZO [24]. The PDA coating introduces a soft organic layer that advances adhesion and diminishes interfacial impedance by establishing a more favorable interaction between the LLZO additive and PEO matrix [25]. This coating also prevents agglomeration by leveraging the adhesive and hydrophilic nature of PDA, ensuring a homogeneous dispersion of LLZO particles within PEO matrix [26]. Crucially, PDA passivates the LLZO surface chemically and physically, masking impurities and stabilizing the interface between ceramic and polymer. The functional groups inherent in PDA, like catechol and amine, further improve ionic transport by synergistically interacting with PEO chains and facilitating Li^+^ ion transport [27].

**FIGURE 1:**
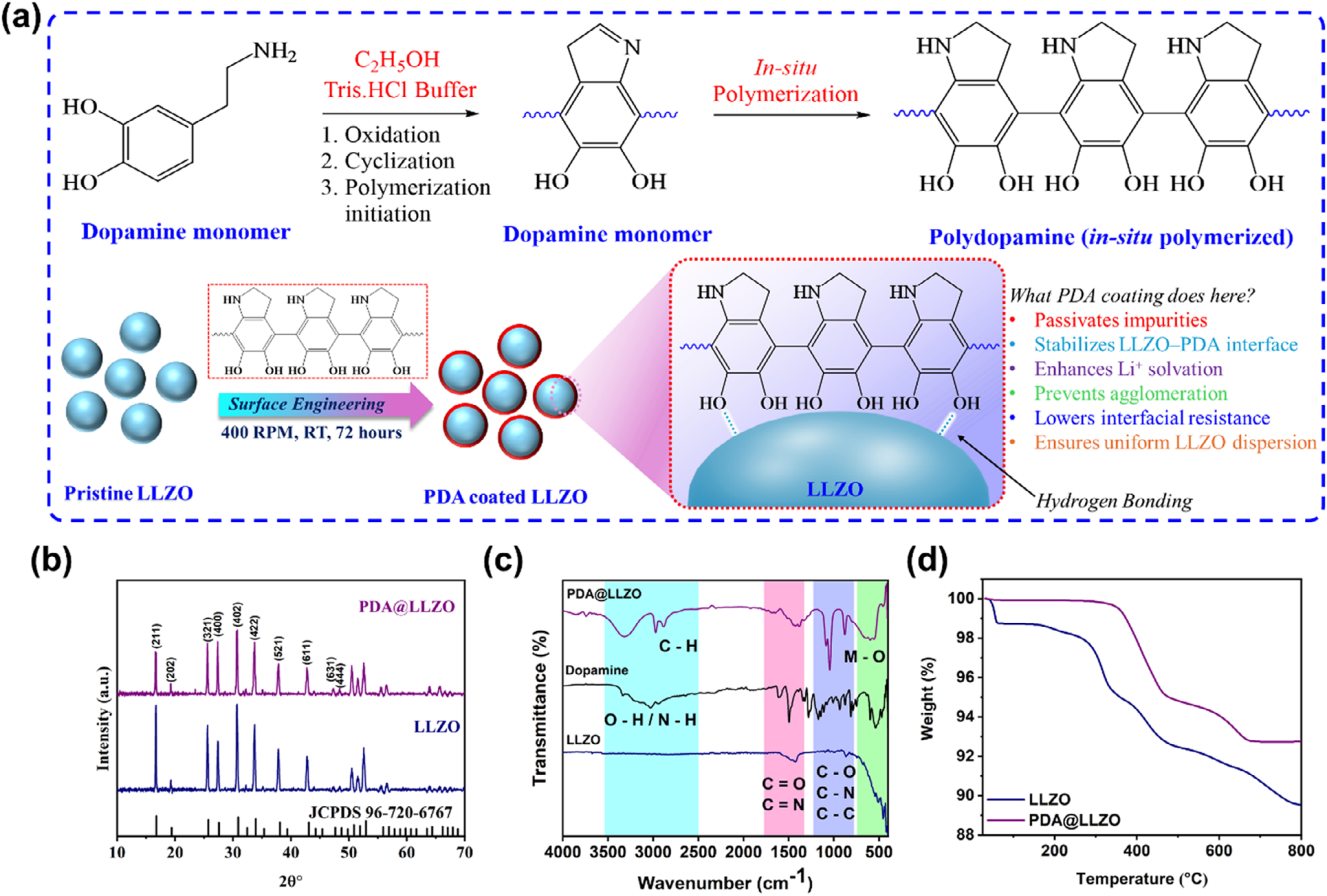
Schematic illustration and characterization of PDA surface modification on LLZO surface. (a) Dopamine undergoes oxidative self‐polymerization in tris–HCl buffer, forming an adhesive PDA layer that uniformly coats LLZO and introduces hydroxyl and amine groups for improved interfacial compatibility. (b) XRD patterns of LLZO and PDA@LLZO confirm phase retention and the presence of PDA peaks. (c) FTIR spectra reveal characteristic PDA bands (O─H, C─H, C═C, C─O) alongside Li─O stretching, verifying successful coating without structural degradation. (d) TGA curves show reduced weight loss and improved thermal stability of PDA@LLZO, confirming the protective role of the PDA layer.

In addition to advancing ionic conductivity of LLZO, PDA coating improves its electrochemical stability, thereby mitigating the unwanted side reactions and establishing a durable interphase when in contact with PEO‐matrix and Li‐metal anode. This stabilization results in improved cycling performance and decreases interfacial resistance over extended cycling. Additionally, the flexibility induced by the PDA coating enables LLZO particles to disperse uniformly within the PEO‐matrix, supporting the mechanical strength of CSE. Hence, the PDA induced modification on LLZO surface efficiently addresses the intrinsic challenges linked with pristine LLZO and notably improves the overall performance of PEO‐based SSEs. Thus, the successful deposition of PDA layer onto LLZO surface was accomplished through an in situ oxidative polymerization approach (Figure 1a), in which dopamine hydrochloride was dissolved in ethanol and buffered to mildly alkaline conditions with pH range between 8.5 and 10.0, using tris–HCl buffer solution. Upon initiation of stirring at ambient temperature, the solution exposed a rapid color change from off‐white to deep brown within first 1–2 min, indicating the oxidation and self‐polymerization of dopamine resulting in PDA formation. This colorimetric transition which is a characteristic of PDA formation reflects the onset of catechol oxidation, cyclization, and subsequent π‐conjugated polymer assembly (Figure S2), as previously reported for surface‐selective PDA film growth in alkaline environments [12].

LLZO powder incorporated into the polymerizing medium remained stayed during 24 h of stirring, ensuring the homogeneous deposition of PDA through non‐covalent π–π interactions and hydrogen bonding at particle‐solution interfaces. The resulting powder revealed an evident visual transformation from white to dark brown and a considerable refinement in surface morphology. This conflict in appearance functions as a qualitative marker for surface engineering and confirms the formation of a conformal PDA layer. The adherence of the coating was evidently robust, aligning with literature revealing the PDA's strong tendency for oxide surfaces via bidentate catechol coordination and auxiliary amine functionality [11]. This facile and scalable surface engineering approach not only modifies the surface chemistry of LLZO but also creates a nitrogen‐ and oxygen‐rich organic interface capable of facilitating the interfacial interactions, improving electrochemical compatibility, and empowering the subsequent improvements in PEO‐LLZO composite performance [28].

X‐ray diffraction (XRD) analysis was conducted to examine the structural stability of LLZO following PDA surface modification (Figure 1b). Both pristine and PDA‐coated LLZO samples demonstrated well‐defined diffraction patterns corresponding to the cubic garnet phase (JCPDS: 96‐720‐6767), proving that the intrinsic crystal structure remained unaffected throughout the coating process. The prominent diffraction peaks observed at 2*θ* ≈ 16.8°, 19.4°, 25.8°, 27.6°, 30.9°, and 33.9° correspond to the (211), (202), (321), (400), (402), and (422) crystallographic planes of the cubic LLZO, respectively. The sustained presence and alignment of these characteristic reflections in both pristine LLZO and PDA@LLZO samples substantiate that the cubic garnet structure was completely preserved following the PDA coating process.

In contrast, the PDA@LLZO sample exhibited the consistent diffraction pattern characterized by reduced peak intensities and marginal peak broadening, particularly around the principal crystallographic planes. This phenomenon is attributed to the interfacial disorder induced by the amorphous PDA layer rather than any core phase degradation or impurity formation, as no extraneous peaks were observed. Primarily, PDA coating facilitates molecular‐level tuning of the interfacial structure, where the preservation of bulk crystallinity confirms structural stability, while localized surface disorder is expected to influence interfacial ion transport. The XRD results thus substantiate a synergistic design that sustains lattice integrity while introducing a disordered interface to improve electrolyte performance via structure‐directed ion transport mechanisms.

Fourier‐transform infrared (FTIR) spectroscopy was utilized to reveal the surface chemistry evolution at LLZO surface subsequent to PDA functionalization (Figure 1c). The spectral analysis of pristine LLZO demonstrates a featureless spectral region above 1500 cm^−^^1^, indicating the absence of organic moieties. A distinct band below 700 cm^−^^1^ corresponds to metal─oxygen (M─O) stretching vibrations of La─O and Zr─O bonds, validating the garnet oxide structure and its chemically inert surface. Upon PDA modification, the PDA@LLZO spectrum unveils a series of new vibrational features reflecting those of dopamine, confirming efficient surface engineering. The emergence of a broad peak between 3400‐3200 cm^−^^1^ is attributed to O─H and N─H stretching, originating from hydroxyl and amine groups in the catechol and amine functionalities of PDA. Concurrently, Additional peaks at 2950–2850 cm^−^^1^ correspond to aliphatic C─H stretching, revealing the polymeric chain formations. The most noticeable transformations are observed in 1650–1450 cm^−^^1^ region, where intense C═O and C═N stretching modes are evident, characteristic of quinonoid and imine structures formed during oxidative self‐polymerization of dopamine. These characteristics are absent in pristine LLZO, verifying their organic interphase origin. Additionally, absorptions in the 1300–1000 cm^−^^1^ regions, reflecting C─O, C─N, and aromatic C─C vibrations, further validate the presence of catecholamine backbone in PDA layer. Hence, FTIR confirms the formation of a chemically surface‐engineered PDA nanolayer on LLZO, establishing a multi‐functional interface that reconciles structural and chemical disparities in hybrid SSEs.

Thermogravimetric analysis (TGA), coupled with derivative thermogravimetry (DTG), was engaged to explore the thermal stability and compositional dynamics of pristine LLZO and PDA@LLZO particles. As illustrated in Figure 1d, the thermogravimetric traces disclose distinct mass loss events and degradation pathways modulated by surface functionalization, offering mechanistic insight into the thermal behavior of organic–inorganic interface. For pristine LLZO, a progressive multistep weight loss is observed from ambient temperature to 800°C. The initial decline near 100°C is ascribed to the evaporation of physiosorbed moisture and surface hydroxyl groups. Subsequent gradual loss up to 450°C likely derives from trace volatile surface organics or residual precursors. A significant reduction between 450 and 650°C may reflect surface reconstruction or destabilization of metastable species. These processes are corroborated by DTG profile (Figure S3), revealing broad peaks at about 280°C and sharper transitions between 450 and 500°C, indicating thermally activated rearrangement steps or oxygen sub‐lattice reordering during garnet phase stabilization. In contrast, the PDA@LLZO profile exhibits a distinctive thermal behavior. The initial stable phase up to 300°C signifies the resilience of LLZO structure. A sharp decline between 300 and 500°C, absent in pristine LLZO, corresponds to oxidative degradation of the PDA coating. This phase captures the exothermic breakdown of catecholamine‐based polymer matrix through the cleavage of C─N and C─O bonds. The corresponding DTG profile reveals a broad peak in this region, indicating the overlapping decomposition events. Following the decomposition, the curve levels off beyond 500°C, representing the sustained integrity of LLZO core, with thermal evolution like pristine LLZO up to 800°C.

These comparative TGA/DTG analyses reinforce the presence and subsequent evaporation of PDA, confirming the successful surface modification. Remarkably, the PDA@LLZO composite exhibits a slightly delayed initiation of mass loss which implies a transient thermal shielding effect by the polymer shell, potentially advantageous for the applications requiring heat buffering heat‐buffering potential. In design perspective, the PDA layer provides low‐temperature processability while maintaining the durability at high‐temperature, thereby supporting its dual functionality in interface engineering for LMB systems. These thermal analyses verify both the compositional integrity and thermally stable characteristics of PDA@LLZO. The system demonstrates controlled degradability below 500°C and ceramic endurance above, making it highly ideal for applications demanding stability across wide temperature regimes.

Differential scanning calorimetry (DSC) was utilized to investigate the thermal characteristics and energetic transitions of pristine LLZO and PDA@LLZO within a temperature range of 0–400°C (Figure S4). The resulting thermograms uncover valuable insights into the impacts of PDA functionalization on interfacial energetics and phase performance, revealing its stabilizing function in thermally dynamic environments like solid‐state LMB systems. The DSC curve of pristine LLZO exhibits a distinctive endothermic peak in 100–200°C interval, typically correlated with desorption of physiosorbed H_2_O and the release of weakly bonded volatile compounds or solvents. This is succeeded by a broader thermal transition at about 300°C, potentially indicative of subtle structural reconfigurations or initial phase transitions within LLZO lattice, likely linked to surface defect relaxation or restructuring of Li‐subdomains.

Conversely, the DSC thermogram of PDA@LLZO reveals a smoother thermal profile characterized by a broad, less intense endothermic events between 100 and 300°C, aligning with gradual thermal degradation of PDA layers. The absence of sharp transitions in this region indicates that PDA layer functions as a thermal buffer, absorbing and dispersing thermal energy in a more gradual manner. Furthermore, the initiation of thermal events is marginally shifted to elevated temperatures, indicating that the PDA shell improves interfacial thermal resistance and retards structural transformations or decomposition. Notably, the reduced heat flow intensity at elevated temperatures between 300 and 400°C in PDA@LLZO, compared to distinct endotherm in pristine LLZO, further underscore the perception that organic interphase mitigates thermally induced phase evolutions. This mitigation is likely attributed to the hydrogen‐bonding framework and π–π interactions present within PDA layer, which alleviate local stress and suppress abrupt structural reactions to thermal input. Hence, these findings encourage that PDA functionalization not only modifies the surface chemical landscape of LLZO but also improves its thermal resistance, facilitating smoother energy transfers and restraining unwanted phase evolutions.

**FIGURE 2:**
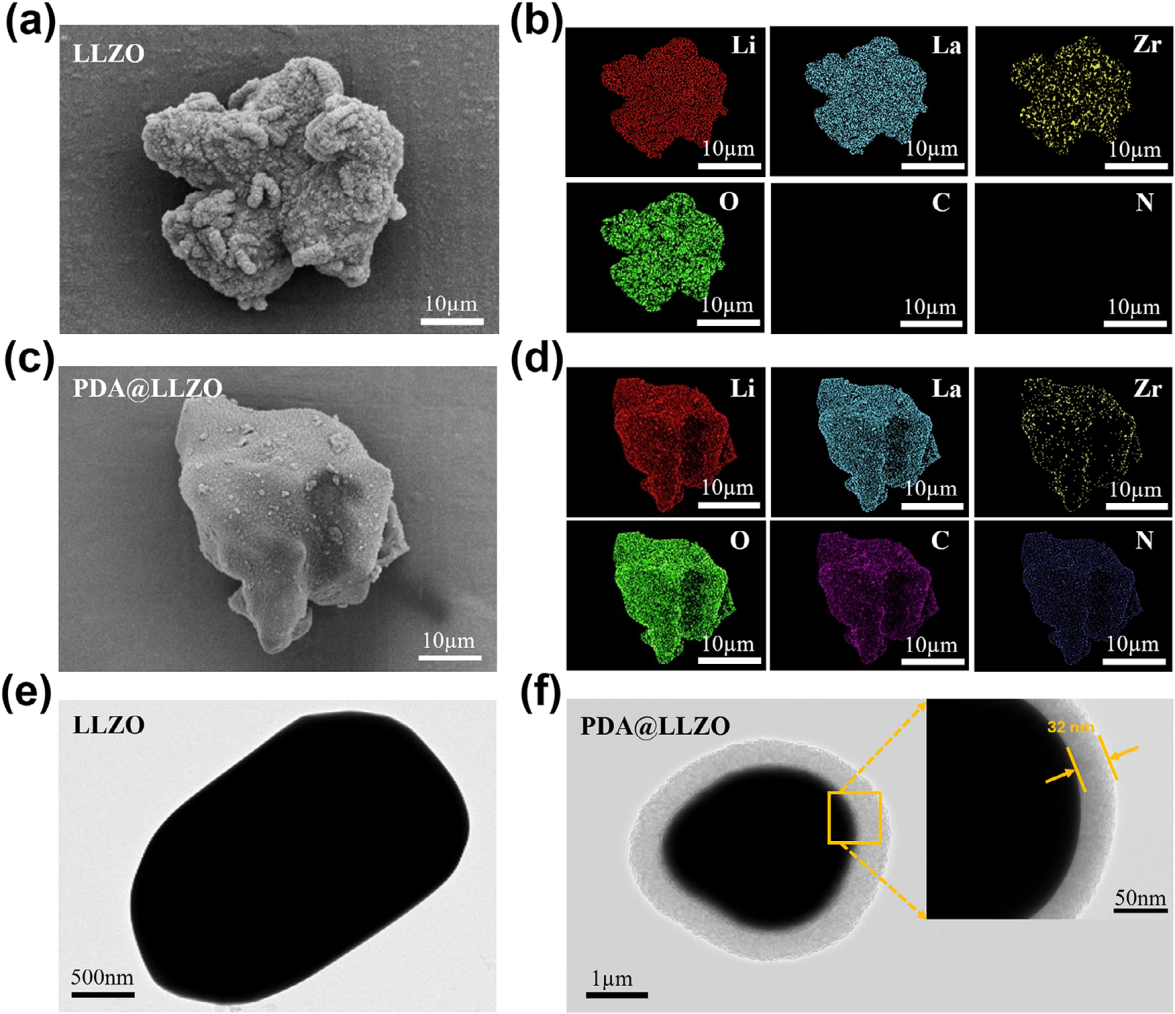
SEM, EDS, and TEM characterization of pristine LLZO and PDA@LLZO. (a) SEM image and (b) EDS mapping of pristine LLZO showing uniform distribution of Li, La, Zr, and O without detectable C or N, confirming its phase purity. (c) SEM image and (d) EDS mapping of PDA@LLZO revealing additional C and N signals uniformly distributed across the surface, verifying successful and homogeneous PDA coating. (e) TEM image of pristine LLZO showing a clean, crystalline surface, (f) TEM image of PDA@LLZO, displays a clear core–shell structure with an amorphous PDA layer uniformly covering the LLZO core, confirming effective surface modification.

To uncover the physicochemical consequences of surface functionalization, field‐emission scanning electron microscopy (FE‐SEM) coupled with energy‐dispersive X‐ray spectroscopy (EDS) was employed to assess morphological and compositional evolution of both pristine and PDA‐functionalized LLZO particles (Figure 2a–f). The pristine LLZO microstructure (Figure 2a) shows an uneven and porous morphology characterized by irregular particle surfaces and prominent intergranular voids. These surface irregularities can function to impede the Li^+^ flux, leading to increased interfacial resistance and reduced mechanical integrity when interfaced with polymeric or electrode binding materials. Elemental mapping (Figure 2b) demonstrates a homogeneous distribution of La, Zr, and O throughout the particle surface, confirming the stoichiometric accuracy and phase purity of LLZO particles. Particularly, the absence of carbon and nitrogen signals endorses the pristine nature of surface, which lacks the chemical versatility, necessary to construct robust interfacial interactions despite its structural crystallinity. This interpretation is further revealed by EDS spectrum (Figure S5), where the main peaks correspond to O (48.25 wt%), La (37.62 wt%), and Zr (14.13 wt%), aligned with expected garnet structure. The absence of C and N further corroborates the chemically inert nature of the LLZO surface, underscoring the imperative for tailored interfacial modifications to address its inherent passivity. Following the PDA functionalization, a dramatic morphological evolution is reflected. As illustrated in Figure 2c, the PDA@LLZO particles reveal a more compact and consolidated structure with substantially reduced porosity. The surface topography infers successful deposition of a conformal PDA layer, which homogenizes the particle interface and enhances particle–matrix connectivity. This microscale surface refinement is critical for reducing interfacial voids and creating continuous ionic pathways within CSE architectures.

EDS elemental mapping of PDA@LLZO (Figure 2d) offers direct compositional evidence of PDA incorporation on LLZO surface, with evident signals corresponding to C and N evenly dispersed alongside La, Zr, and O. Despite the low intensity of the nitrogen signal, its presence is particularly significant as it specifies the existence of imine and amine groups, key features of PDA's catecholamine‐rich framework that facilitate π–π stacking, hydrogen bonding, and improved electrochemical coupling at the interface. This finding is further supported by quantitative EDS spectroscopy shown in Figure S6, where noticeable peaks corresponding to C (19.45 wt%) and N (1.48 wt%) emerge while the oxygen content reduces to 25.71 wt%, because of partial masking of LLZO surface signal by coated organic layer. La (40.43 wt%) and Zr (12.93 wt%) remain relatively constant, indicating that the bulk composition is sustained and PDA coating is strictly superficial. These results justify that the PDA coating layer is not only morphologically continuous but also chemically integrated, developing a chemically active interfacial nanolayer.

High‐resolution transmission electron microscopy (HR‐TEM) reveals compelling visual demonstration of interfacial and morphological changes induced by PDA surface functionalization. Pristine LLZO particles (Figure 2e) expose a sharply faceted structure with smooth, unremarkable surfaces, attribute of pristine LLZO crystallites. The absence of any peripheral amorphous contrast in high‐magnification images confirms that LLZO surface remains chemically unmodified, as shown by FE‐SEM & EDS results. This clean, native interface, while structurally robust, provides marginal chemical functionality for interfacial bonding or ionic mediation. In contrast, surface modified LLZO particles with PDA layer (Figure 2f) demonstrate a distinct core–shell architecture, where each garnet particle is uniformly enveloped by a conformal, low‐density amorphous layer. This outer layer, with a thickness of 25–35 nm, is attributed to the PDA coating and appeared uniformly distributed across the particle surface in HR‐TEM. Multi‐scale elemental characterization was employed to establish the chemical composition of the particles. SEM‐EDS elemental mapping revealed a measurable nitrogen signal exclusively in PDA@LLZO sample, while TEM‐coupled EDS elemental mapping at the particle scale (Figure S7) and at the coating‐edge scale (Figure S8) further corroborated the uniform spatial distribution of N and C within the amorphous shell region, with a progressive diminution of Zr and La signals toward the outer edge, directly confirming the core–shell elemental separation between the crystalline LLZO core and the homogeneous PDA coating. The amorphous nature of coating layer distinguishes it from the crystalline core and reflects the disordered polymeric composition of PDA, characterized by abundant catechol, amine, and imine functionalities. Importantly, this coating is not a passive morphological layer but rather a chemically active interface that facilitates multiple functions critical for LMBs [29].

**FIGURE 3:**
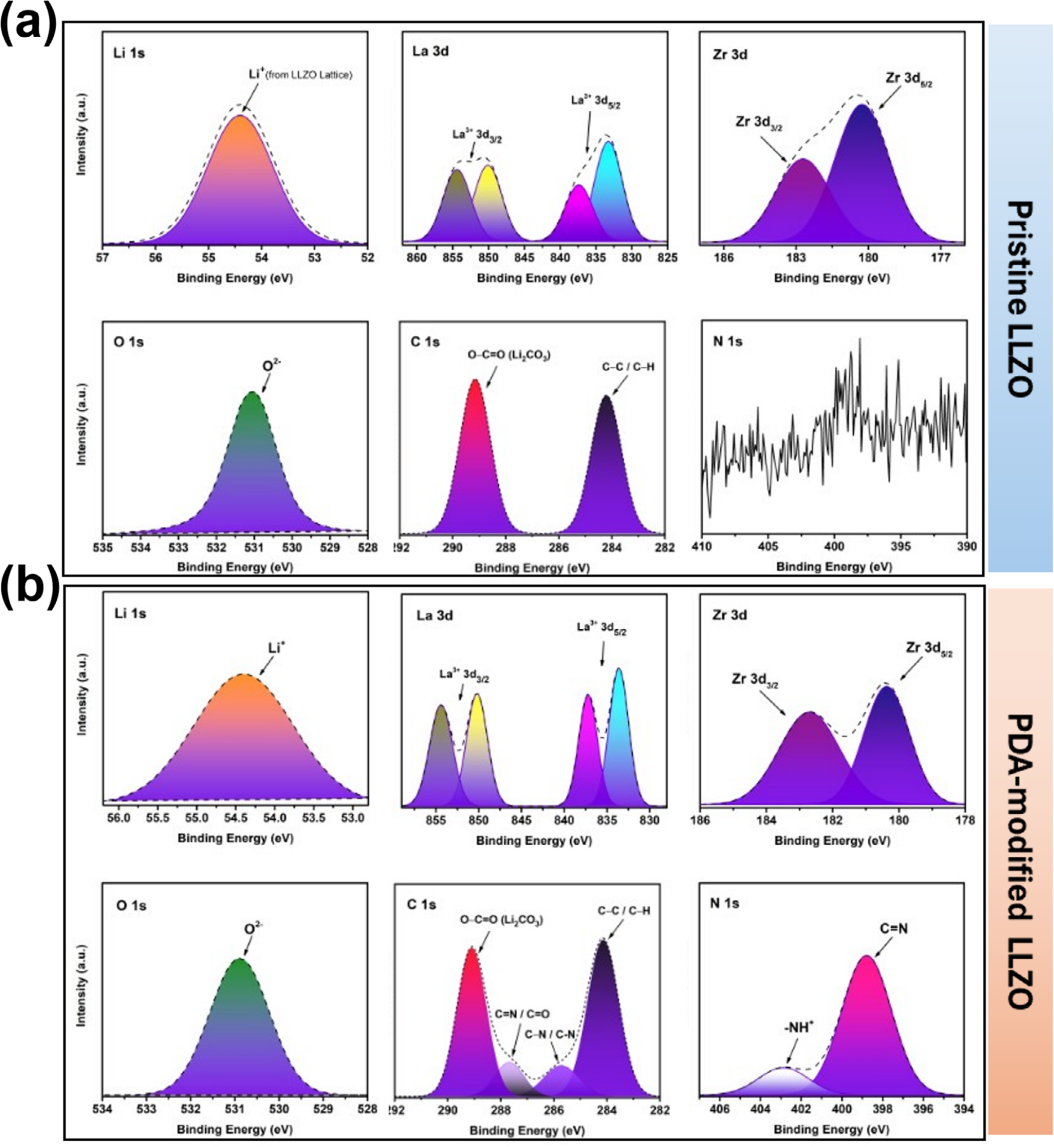
High‐resolution XPS spectra of (a) pristine LLZO and (b) PDA@LLZO showing chemical changes after surface modification. The unchanged Li 1s, La 3d, Zr 3d, and O 1s peaks confirm structural integrity of LLZO, while the new N 1s signal in PDA@LLZO verifies nitrogen‐containing PDA deposition. Deconvoluted C 1s and N 1s spectra reveal O─C═O, C═N/C═O, C─N/C─O, C═N, and ─NH^+^ species, characteristic of oxidized catecholamine structures, indicating enhanced interfacial functionality without compromising the garnet framework.

To uncover the chemical and electronic transformations induced by PDA surface modification, X‐ray photoelectron spectroscopy (XPS) was utilized to examine both pristine and PDA‐coated LLZO (Figure 3a,b). The obtained results reinforce that while the bulk LLZO framework remains unchanged, PDA functionalization creates a surface chemistry enriched in nitrogen and oxygen, thereby modifying the interfacial environment while maintaining the structural integrity [30].

The Li 1s spectra of both samples show distinct peaks at 54.7 eV for the pristine sample and 54.5 eV for PDA@LLZO, which suggests that the Li^+^ environments are intact and that no decomposition products like LiOH or Li_2_CO_3_ are present. This stability in chemical composition demonstrates that PDA coating keeps the ionically active lattice necessary for long‐range Li^+^‐ion transport. In contrast, the C 1s and N 1s spectra provide distinct evidence of surface modification. Both pristine LLZO and PDA@LLZO exhibit a prominent hydrocarbon peak at 284.6 eV, along with a higher binding energy component at 289.0 eV attributed to surface carbonate (O─C═O) species, specifically Li_2_CO_3_, formed after air exposure. Interestingly, the PDA@LLZO sample exposes additional contributions in the 286–288 eV region, which are assigned to C─N/C─O and C═N/C═O functionalities originated from the polydopamine coating. Particularly, the C─N/C─O component originates from secondary amine and hydroxyl groups, while the C═N/C═O contribution corresponds to quinonoid imine and carbonyl species formed during the oxidative polymerization of PDA.

Furthermore, the emergence of N 1s peaks in PDA@LLZO provides direct evidence of successful surface functionalization. Notably, two separate N 1s peaks appear at 398.5 and 401.5 eV, associated with C═N and protonated ─NH^+^ groups. These functional sites, characteristics of PDA, function as Lewis base centers and hydrogen‐bonding anchors that can modulate Li^+^ solvation and interfacial ion movement. The La 3d and Zr 3d spectra show no chemical variations, exhibiting characteristic La^3^^+^ (834.8 eV) and Zr^4^^+^ (180.2 eV) peaks, thereby confirming that the underlying oxide structure remains unaffected by redox perturbation. Similarly, the O 1s spectrum reveals a prominent lattice oxygen peak at 531.0 eV, with no high‐binding‐energy shoulders that would typically indicate hydroxyl or adsorbed water species. This suggests that PDA coating layer establishes a uniform, moisture‐resistant barrier that prevents Li loss and improves stability in ambient conditions.

To quantitatively evaluate whether the surface polymerization process perturbed the bulk composition of the LLZO core, inductively coupled plasma optical emission spectroscopy (ICP‐OES) analysis was conducted on pristine LLZO and PDA@LLZO using 10 mg of each sample under identical calibration and digestion conditions. The elemental concentrations obtained were systematically converted into molar quantities and normalized to determine the stoichiometric ratios (Table S1). Pristine LLZO showed a Li:La:Zr molar ratio of 7.01:2.99:1.99, which closely aligns with the nominal stoichiometry of Lithium Lanthanum Zirconium Oxide (Li_7_La_3_Zr_2_O_12_), confirming the compositional integrity of the initial material. Following PDA deposition, the ratio remained largely unchanged at 6.98:2.97:1.98, suggesting that the coating process did not cause significant Li depletion, cation leaching, or compositional redistribution within the detection limits of the ICP‐OES.

The slight deviation between pristine and coated samples (≤0.03 for Li and ≤0.02 for La and Zr) is within the range of experimental uncertainty, indicating that the bulk cationic framework is preserved during surface modification. This compositional consistency, along with the preserved cubic garnet patterns observed in X‐ray diffraction (XRD) and the detection of N‐containing surface species identified by X‐ray photoelectron spectroscopy (XPS), confirms that the PDA layer forms a conformal coating while preserving both the stoichiometry and crystallographic structure of the LLZO core.

**FIGURE 4:**
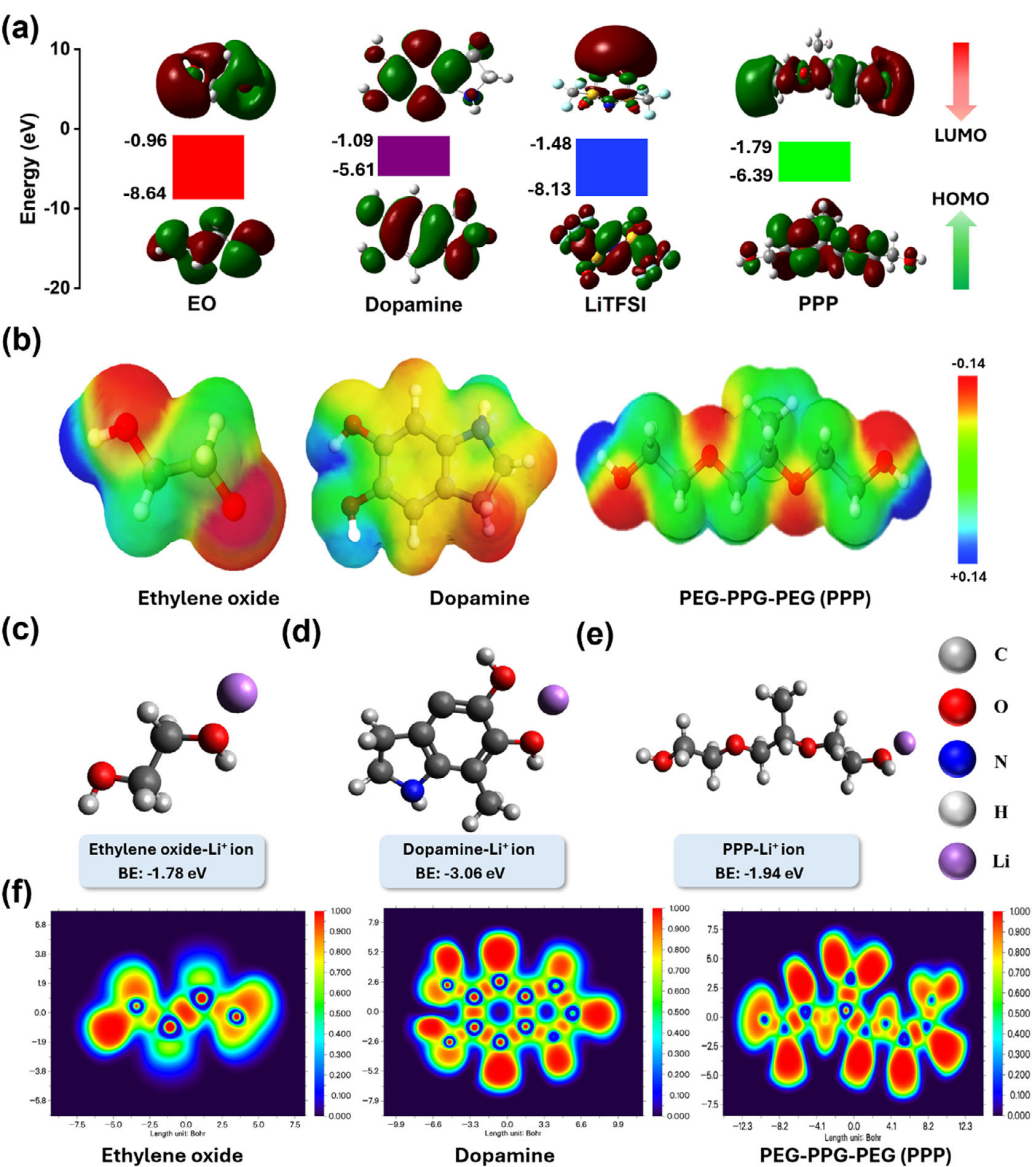
DFT analysis of electronic and interfacial properties of electrolyte components. (a) HOMO–LUMO energy levels of EO, dopamine, LiTFSI, and PPP reveal their distinct electronic gaps, with PEO showing the widest (7.68 eV) and dopamine the narrowest (4.52 eV), indicating its higher reactivity. (b) MEP surfaces highlight electron‐rich sites on ether oxygens and catechol groups as Li^+^ coordination centers. (c–e) Calculated Li^+^ binding energies show dopamine (‐3.06 eV) > PPP (‐1.94 eV) > EO (‐1.78 eV), confirming dopamine's strongest Li^+^ affinity. (f) ELF maps display dopamine's localized electron density and PPP's delocalized regions, illustrating cooperative Li^+^ transport and anion repulsion.

To reveal the fundamental electronic properties and potential molecular interactions of the electrolyte components, density functional theory (DFT) computations were conducted utilizing the Gaussian 09 W software package. The geometries were fully optimized using the B3LYP exchange‐correlation functional paired with the 6‐31G(d) basis set [31], and the frontier molecular orbitals were computed to find out the HOMO–LUMO energy levels of each species (Figure 4a; Table S2). These electronic descriptors function as indicators of relative oxidative stability, electron‐donating or accepting capabilities, and potential coordination sites that are essential for electrolyte performance. The ethylene oxide (EO) exhibited a LUMO at ‐0.96 eV and a HOMO energy of −8.64 eV, resulting in an energy bandgap (*E*_g_) of 7.68 eV. Such a wide gap implies excellent electrochemical stability and low electronic conduction, which reinforces its function as an insulating ion‐conducting polymer [32, 33]. Conversely, dopamine presents a narrower band gap of 4.52 eV with its HOMO energy of ‐5.61 eV, and LUMO energy of −1.09 eV, suggesting its electron‐rich character and potential to develop π‐π interactions or hydrogen bonding with adjacent molecules [34]. The LiTFSI salt revealed a HOMO energy of −8.13 eV and a LUMO of −1.48 eV, resulting in an energy gap of 6.65 eV, consistent with its role as a stable ionic conductor exhibiting high dissociation. Interestingly, the poly(propylene glycol)‐block‐poly(ethylene glycol)‐block‐poly(propylene glycol) (PPP) component unveiled a HOMO energy of −6.39 eV and a LUMO energy of −1.79 eV, leading to a gap of 4.60 eV. The electron‐dense ether linkages present in PPP may function as coordination sites for Li^+^‐ion, enhancing the ionic mobility and contributing to mechanical flexibility of electrolyte system [35, 36].

To rationalize the electronic surface topology and potential of ion‐dipole interactions among molecular components, molecular electrostatic potential (MEP) maps and total electron density (TED) distributions were calculated. The MEP profiles of EO, dopamine, LiTFSI, and PPP (PEG–PPG–PEG) (Figure 4b) show distinct areas of electron abundance and deficiency, which play crucial role in determining the local Li^+^‐ion coordination and salt dissociation behavior. The MEP surface of EO discloses highly negative electrostatic regions (‐0.14 a.u., red regions) surrounding the ether oxygen atoms, affirming their function as key sites for Li^+^ solvation [37, 38]. Dopamine features a polarizable catechol ring with electron‐rich hydroxyl groups and a slightly less electronegative amino group, providing multi‐dentate coordination sites that improve interfacial compatibility when grafted onto the LLZO surface. The PPP segment displays alternating zones of negative and neutral potentials throughout the ether and methyl‐rich regions, indicating that mobility driven by segmental flexibility facilitates ion hopping along the flexible framework [39]. These characteristics collectively underpin the excellent ionic mobility in CSE‐30 by creating continuous pathways for Li^+^‐ion transport. For LiTFSI (Figure S9), the electronegative TFSIˉ groups show deep electrostatic wells, enabling facile salt dissociation while ensuring electrochemical stability.

To further validate these electronic characteristics under more realistic polymeric conditions, MEP distributions were computed for oligomeric models of PEO_(10)_, PDA_(3)_, and PPP_(3)_ (Figure S10). The polymeric MEP profiles closely resembled those of the corresponding monomeric units, indicating that the electrostatic landscape was primarily dictated by local functional groups rather than extended chain length. Specifically, PEO_(10)_ exhibited continuous negative potential regions along the polymer backbone, centered on ether oxygen atoms, suggesting the formation of extended Li^+^‐coordination pathways. Similarly, PDA_(3)_ retains a strongly localized negative potential around the catechol and oxygen‐containing sites, with only minor spatial delocalization upon oligomerization, thereby preserving its multidentate coordination capability. In PPP_(3)_, distributed negative regions across the ether linkages coexist with relatively neutral methyl‐rich domains, forming a heterogeneous yet continuous electrostatic network that supports Li^+^ migration through segmental motion. Importantly, no qualitative redistribution of the electrostatic potential was observed upon transitioning from the monomeric to the oligomeric form, confirming that the primary coordination‐active sites remained unchanged. This consistency demonstrates that the MEP‐derived interpretation of Li^+^ interaction and transport is robust and not an artifact of simplified molecular models, thereby validating its relevance to the experimentally realized polymeric interphase.

The TED maps offer qualitative insights into the distribution of electron density across molecular structures, serving primarily as a visual representation of electron localization rather than a direct measure of electronic delocalization (Figure S11a–d). Owing to their dependence on iso‐value selection and molecular size, a direct comparison of TED iso‐surfaces across different molecules is inherently limited. However, regions of concentrated electron density can be linked with coordination‐active sites and structural‐stabilization effects [40]. EO and PPP demonstrated spatially extended electron density distributions along the polymer chains, whereas dopamine and LiTFSI exhibited more localized densities around specific functional groups, reflecting their distinct interfacial roles. These observations are aligned with the MEP analysis, which offers a more precise and comparable description of the electronic environment governing Li^+^ coordination.

In order to understand the molecular‐level insights into the selective coordination processes governing the ionic transport within CSE, DFT calculations were carried out at B3LYP/6‐31G(d) level to assess the binding energies (BEs) between Li^+^, TFSI^−^ ions and key molecular components comprising of EO, dopamine, and PPP. The calculated BEs of Li^+^ reveal a distinct affinity ranking: dopamine‐Li^+^ (−3.06 eV) > PPP‐Li^+^ (−1.94 eV) > EO‐Li^+^ (−1.78 eV). This pattern underscores the significantly stronger Li^+^ coordination capability of dopamine, attributed to its electron‐rich catechol and amine groups, which offer several active sites for Li^+^ interaction (Figure 4c–e). This strong interaction is predictable to enhance interfacial adhesion and regulate Li^+^ coordination at the PEO–LLZO interface, leading to transient Li^+^ immobilization without permanent trapping, thereby contributing to improved structural stability and promoting directional Li^+^‐ion flux [40]. Such localized and dynamically reversible coordination enables Li^+^ migration through a hopping mechanism between adjacent coordination sites while ensuring sufficient mobility for long‐range transport, consistent with prior reports on polymer electrolytes, where strong yet non‐blocking coordination supports ion conduction rather than obstructing it [41, 42].

The flexible ether linkages enriched PPP segment also demonstrates significant affinity for Li^+^ ions, surpassing that of the conventional PEO matrix and suggesting its essential role in ionic coordination within bulk‐phase. Because the experimentally formed interfacial layer possesses an intrinsically polymeric nature, additional examination was carried out to explore whether the oligomer length influences the computed Li^+^‐binding energetics to ensure that the above conclusions are not an artifact of monomer‐level modeling. Therefore, representative oligomeric structures were constructed and systematically compared with their monomeric counterparts (Figure 4c; Figure S12). For PEO, the binding energies of PEO_(1)_–Li^+^ and PEO_(10)_–Li^+^, corresponding to one and ten EO repeat units, respectively, were −1.78 and −1.82 eV, demonstrating that Li^+^ coordination was dominated by a local ether‐oxygen environment and rapidly converged with increasing chain length. An analogous trend was observed for the PDA‐related motif, where the binding energy changed only marginally from −3.06 eV for PDA_(1)_–Li^+^ to −3.13 eV for PDA_(3)_–Li^+^, corresponding to one and three dopamine repeat units (oligomer), respectively. This nominal variation exposes that the Li^+^ interaction remains primarily localized at the catechol and oxygen‐containing coordination sites, while extended oligomeric chain structures contribute only limited cooperative stabilization effects.

Similarly, for the PPP‐containing system, the binding energy increased marginally from −1.94 eV for PPP_(1)_–Li^+^ to −2.01 eV for PPP_(3)_–Li^+^, reflecting a modest cooperative contribution while maintaining an intermediate Li^+^ affinity accompanying with the ether‐rich backbone. This behavior further supports the role of PPP in creating a comparatively flexible coordination environment that facilitates Li^+^ transport without inducing strong ion‐trapping.

Importantly, the relative energetic hierarchy remained consistent upon oligomerization, with PDA consistently demonstrating a substantially stronger Li^+^ affinity compared to PPP and PEO. This result confirms that the mechanistic interpretation derived from a single monomer is robust and governed by intrinsic local coordination chemistry rather than model simplification, thereby validating the use of representative molecular fragments while simultaneously aligning the computational analysis with a realistic polymeric interphase. These results reinforce that PDA‐derived chemistry establishes a preferential and strongly interacting coordination environment for Li^+^, while PPP contributes to a dynamically adaptive coordination environment, collectively sustaining the regulated ion transport and interfacial stability observed in the composite system.

In addition to molecular‐level interactions with Li^+^, the interface between dopamine and LLZO was examined to assess its structural affinity using DFT‐based interaction energy (IE) analysis. The computed IE of −1.39 eV (Figure S13a) reveals a stable non‐covalent interaction between the LLZO surface and dopamine. This interaction likely arises from electrostatic attractions and hydrogen bonding between the polar catechol and amine groups of dopamine and the oxygen‐enriched garnet structure of LLZO. Such promising interfacial adhesion is predictable to enhance stable ceramic‐polymer coupling, reduce interfacial delamination, and improve efficient Li^+^ transport across the organic‐inorganic interface [43].

Conversely, interactions with TFSI^−^ anion expose significantly reduced binding affinities across all components. The calculated BEs for dopamine‐TFSI^−^ (−1.05 eV) and EO‐TFSI^−^ (−1.03 eV) suggest merely weak electrostatic interactions (Figure S13b,c), enabling a dissociated ionic environment that enables high Li^+^ mobility. Notably, repeated attempts to achieve a stable PPP–TFSI^−^ complex continually failed to converge (Figure S13d), indicating a computationally substantiated anion‐repelling characteristic of PPP chain. This absence of interaction not only inhibits the anions crowding around polymer chains but also maintains the chemical flexibility of TFSI^−^ to engage in charge transfer [44]. This selective interaction behavior endorses a decoupled ion‐transport mechanism, in which Li^+^ is preferentially coordinated while anion involvement is minimized, thereby facilitating salt dissociation, improving Li^+^ transference, and reducing concentration polarization, as extensively reported for weakly coordinating polymer electrolyte systems [45, 46].

Collectively, these energetic insights reveal that the molecular structure of the CSE is meticulously designed to enable effective and selective Li^+^ coordination while simultaneously reducing anionic interactions. This well‐balanced configuration facilitates efficient salt dissociation, improves overall ionic conductivity, and confirms the development of stable solid‐electrolyte interface (SEI) throughout prolonged cycling operation.

To demonstrate the spatial distribution of electron localization and its significance in ion coordination, electron localization function (ELF) maps were computed for ethylene oxide, dopamine, and PPP (Figure 4d). The ELF profile of EO reveals a relatively symmetrical and localized electron density concentrated around the ether oxygen atoms, characteristic of its restricted but directional Li^+^ coordination by lone‐pair interactions. This localized electron confinement emphasizes the moderate binding strength perceived in EO‐Li^+^ complexes and rationalizes the limited flexibility of the polymer matrix in enabling dynamic ionic transport.

In stark contrast, dopamine demonstrates a significantly delocalized ELF topology, with concentrated electron‐rich zones over the catechol ring and extending toward the amine group. This broad electronic delocalization aligns with its excellent binding energy (‐3.06 eV) and reinforces its potential to function as a multidentate chelator for Li^+^ ions [44]. The conjugated π‐system present in aromatic moiety, combined with the electron‐donating hydroxyl and amino groups, establishes a highly polarizable environment that stabilizes cation coordination while preserving local electronic flexibility which is essential for accommodating the interfacial charge and suppressing the ion clustering.

The ELF of PPP demonstrates a hybridized landscape, exhibiting several scattered lobes of electron localization within its PEG‐rich ether segments, while maintaining conformational flexibility through PPG domains. This diffuse yet continuous electron distribution facilitates a percolative mechanism for Li^+^ hopping across the soft segmental matrix. Additionally, the comparatively reduced ELF intensity around the terminal poly(ethylene glycol) units corresponds with the experimentally observed anion‐repelling nature of PPP, offering a visual explanation for its failure to form stable TFSI^−^ complexes. The extended and spatially dispersed ELF of PPP not only improves polymer segmental motion but also promotes ionic homogeneity by preventing charge crowding and confirming uniform Li^+^ solvation. Hence, the DFT‐derived insights into binding energies, electronic structures, and electron localization function (ELF) mappings present a convincing molecular‐level rationale for the superior performance of the CSE system.

**FIGURE 5:**
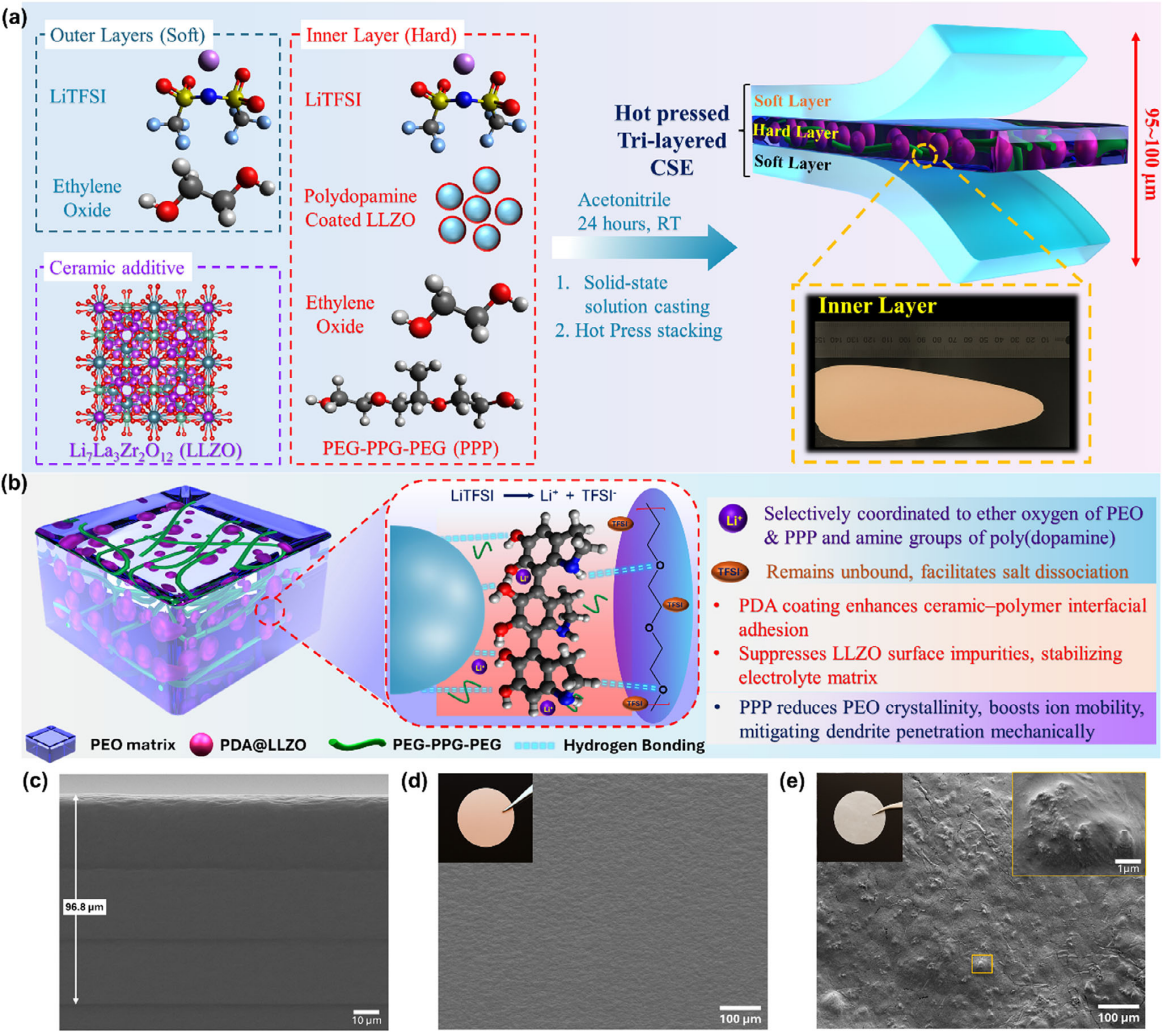
Schematic and morphological analysis of CSE fabrication. (a) Stepwise synthesis of the CSE film involving solvent‐assisted dispersion, tape casting, and thermal lamination for uniform integration of PDA@LLZO and PEG–PPG–PEG (PPP) into a PEO–LiTFSI matrix. (b) 3D rendering of the laminated CSE structure. (c) Cross‐sectional SEM image, showing a 96.8 µm thick, defect‐free multilayer membrane with excellent interfacial cohesion. (d) Top‐view SEM revealing a smooth, crack‐free surface indicative of uniform ceramic dispersion in optimized tri‐layer CSE. (e) Control SEM image highlighting severe LLZO agglomeration in the absence of PDA coating, demonstrating the necessity of surface functionalization for morphological homogeneity and electrochemical stability.

The CSE was prepared through a precisely controlled multi‐step route involving solvent‐mediated dispersion, followed by solution casting and tape casting, and finalized by thermal lamination. Initially, all functional components including ceramic additive (PDA@LLZO), triblock copolymer (PEG–PPG–PEG, denoted as PPP), and polymer salt components (PEO and LiTFSI) were dispersed in acetonitrile (ACN) to achieve a homogenous electrolyte slurry (Figure 5a). The use of ACN facilitated superior solubility and dispersion of all components, enabling intimate molecular‐level homogenization. First, PDA@LLZO powder was mixed into ACN under combined magnetic stirring and sonication to attain uniform distribution. Subsequently, PPP and the PEO–LiTFSI matrix were incorporated to form a composite mixture slurry that was then cast into films using a tape‐casting approach, yielding a flexible, self‐supporting membrane with excellent film‐forming characteristics.

To reinforce structural robustness and develop interfacial compatibility, a tri‐layered membrane was designed by sandwiching a mechanically reinforced CSE as an inner core between two PEO‐LiTFSI outer layers. These layers were assembled and subjected to hot pressing step at adequate temperature and pressure, enabling polymer chain inter‐diffusion and interfacial adhesion. The hot‐pressing step not only facilitated a smooth integration of individual layers into a single compact architecture but also enhanced morphological densification by removing interfacial voids and trapped residual solvents.

As a result, thermally laminated membrane exhibited excellent flexibility while demonstrating improved homogeneity and cohesive strength, compelling it appropriate for practical LMB assembly. The schematic in Figure 5b illustrates the multifunctional role of inner layer, composed of PEO/LiTFSI/PDA@LLZO/PPP, in stabilizing polymer–ceramic CSE. Li^+^‐ions are selectively coordinated to ether oxygens of PEO and PPP, as well as amine groups of polydopamine, thereby facilitating efficient conduction of ions. The PDA functionalization serves a dual role as it effectively improves the ceramic–polymer interfacial coupling while also mitigating LLZO surface impurities and establishes a stabilized electrolyte framework. Simultaneously, incorporation of PPP into PEO suppresses PEO's crystallinity and facilitates ionic transport, while mechanically reinforcing the CSE system against dendrite growth. This synergistic interaction of PPP and PDA@LLZO leads to advanced ionic conductivity and robust mechanical stability, essential for long‐term LMB cycling.

The cross‐sectional SEM of fully laminated membrane (Figure 5c) revealed a compact and uniform morphology with a total thickness of 96.8 µm. Remarkably, no evidence of delamination or interlayer cracking was detected, suggesting excellent compatibility and adhesion between all three functional layers. Such uniform lamination is crucial to avoid interfacial resistance build‐up during cycling and enables long‐range ion transport without interfacial challenges. The surface SEM image of top layer of CSE film (Figure 5d), revealed a smooth, crack‐free morphology, evidencing successful uniform dispersion of ceramic additive and efficient inhibition of phase segregation. This morphology is key to realizing uniform Li^+^‐ion flux across the entire membrane, specifically during prolonged charge–discharge cycling where surface irregularities often behave as dendrite nucleation sites. The critical role of PDA surface functionalization becomes evident in Figure 5e, which exposes the surface morphology of a control sample in which pristine LLZO was incorporated into PEO matrix without PDA layer deposition. Here, large ceramic clusters are clearly visible, suggesting poor interfacial compatibility and phase immiscibility between PEO host and LLZO. These inhomogeneous domains function as mechanical and ionic dead zones, hindering Li^+^ transport and localizing the mechanical stress that may trigger micro‐cracking or dendrite nucleation. Such structural defects compromise both mechanical robustness and ionic conductivity, making the membrane unsuitable for reliable battery operation.

**FIGURE 6:**
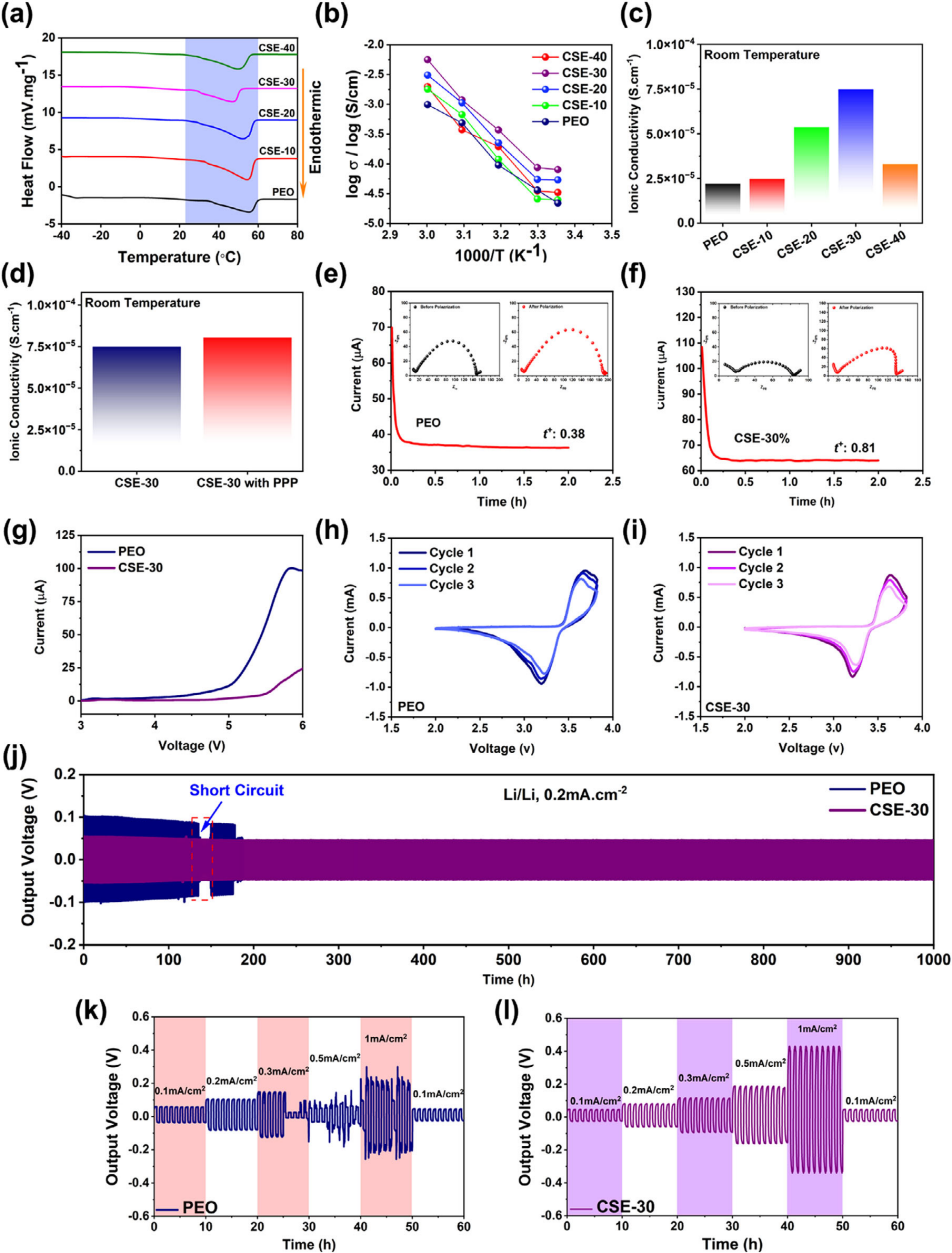
Thermal, ionic, and electrochemical characterization of PEO and CSE‐30‐based electrolytes and cells. (a) DSC traces reveal progressive suppression of PEO crystallinity upon PDA@LLZO and PPP incorporation, with CSE‐30 showing the lowest enthalpy of fusion, confirming enhanced amorphization. (b, c) Arrhenius and room‐temperature conductivity plots show thermally activated Li^+^ transport, with CSE‐30 achieving the highest conductivity due to optimal filler dispersion and matrix flexibility. (d) Comparison of CSE‐30 with and without PPP shows slightly higher conductivity and, (e, f) a significant increase in Li^+^ transference number from 0.38 (PEO) to 0.81 (CSE‐30), indicating restricted TFSI^−^ mobility and selective Li^+^ transport. (g) LSV curves confirm an extended stability window of CSE‐30 beyond 5.5 V. (h, i) CV profiles demonstrate reversible redox behavior for CSE‐30 versus poor reversibility for PEO, evidencing superior interfacial stability. (j) Long‐term Li plating/stripping at 0.2 mA cm^−^^2^ shows dendrite‐free cycling of CSE‐30 over 1000 h with minimal polarization. (k, l) Rate performance of Li/Li symmetric cells shows that PEO fails beyond 0.3 mA cm^−^^2^, while CSE‐30 maintains stable symmetric voltage profiles up to 1.0 mA cm^−^^2^, confirming the robustness of the optimized composite electrolyte.

Differential scanning calorimetry (DSC) was used to examine the thermal transitions and degree of crystallinity in CSEs (Figure 6a). All samples reveal a wide endothermic event between 50 and 60°C, attributed to the melting of crystalline PEO segments. The pristine PEO sample exhibits the sharpest endothermic peak, corresponding to a melting enthalpy (Δ*H*) of 46.78 J g^−^^1^ and a crystallinity of 23.05% (Figure S14). The incorporation of PDA coated LLZO particles together with PPP, a consistent decline in both parameters is observed, reflecting progressive disruption of long‐range chain structural alignment. Among all modified samples, CSE‐30 attains lowest enthalpy of melting at 33.16 J g^−^^1^ (Figure S15) and lowest crystallinity at 16.34%, indicating the strongest suppression of PEO crystalline phase growth. This suppression corresponds to synergistic interfacial interactions, exceptionally the hydrogen bonding developed between the ether oxygen of PEO and PDA, as well as the steric hindrance caused by flexible PPP network and dispersed LLZO particles. CSE‐10 and CSE‐20 present intermediate crystallinity values of 20.09 and 19.37%, respectively, accompanied by Δ*H* values of 40.77 and 39.33 J g^−^^1^. Interestingly, at increased additive loading (CSE 40), a minor increase in crystallinity of about 20.39% and Δ*H* of 41.38 J g^−^^1^ is noted, likely arising from excessive LLZO contents that restrict polymer chain mobility and induces localized recrystallization. These results emphasize the significance of appropriate concentration of additives in polymer microstructural engineering. Reduced crystallinity improves the amorphous phase fraction, which promotes polymer segmental dynamics and facilitates Li^+^‐ion hopping. The optimized composition (CSE‐30) balances strong interfacial cohesion and controlled polymer flexibility, resulting in a promising morphology for superior ionic conductivity and stable electrochemical cycling.

Following the thermal analysis, structural optimization of the tri‐layered CSEs was conducted by varying the relative layer thicknesses to evaluate the impact of layer geometry on ionic transport and interfacial stability. To this end, three thickness‐controlled variants; CSE‐30 (1:1:1), CSE‐30B (0.5:2:0.5), and CSE‐30C (0.25:3.5:0.25) were systematically designed and evaluated with respect to their electrochemical stability, ionic conductivity, and interfacial dynamics to rationally determine the most effective structural configuration for subsequent device‐level exploration. Each configuration maintained an identical overall thickness but differed in the relative dimensions of the outer and inner layers, thereby enabling a direct assessment of how layer geometry governs ion flux, stress distribution, and dendrite mitigation in symmetric Li∣Li and full‐cell configurations.

During galvanostatic cycling, the Li∣CSE‐30∣Li cell exhibited perfectly symmetric and stable voltage oscillations from 0.1 to 1.0 mA cm^−^^2^ without any indication of short‐circuiting, underscoring its balanced ionic transport and mechanical robustness. Conversely, both CSE‐30B and CSE‐30C exposed a continuous polarization increase at 0.5 mA cm^−^^2^, with subsequent voltage collapse at 1.0 mA cm^−^^2^, reflecting dendritic propagation through the thinner outer layers (Figures S16 and S17). This comparative observation determines that balanced layer thickness ensures the most uniform stress distribution and homogeneous interfacial current density, effectively mitigating localized Li plating and preventing mechanical failure under higher current loads. The uniform tri‐layer design therefore ensures that the LLZO‐enriched inner layer maintains uninterrupted Li^+^ conduction while the outer polymer‐enriched layers provide enough flexibility to accommodate the interfacial strain.

Quantitative transport analyses further provide additional support for these results. The Li^+^‐ion transference number $\left(t_{Li^{+}}\right)$ follows the trends as CSE‐30> CSE‐30B > CSE‐30C, with corresponding values of 0.81, 0.76, and 0.71, respectively (Figures S18 and S19). The significantly higher $t_{Li^{+}}$ of CSE‐30 indicated a more selective Li^+^ conduction pathway due to well‐distributed PDA@LLZO interfaces and diminished anion mobility. The equal‐thickness architecture improves the interfacial coordination environment, where the electron‐rich PDA coating on LLZO establishes hydrogen‐bonded networks with PEO chains, enabling improved Li^+^ desolvation and transfer while maintaining structural integrity. The lower $t_{Li^{+}}$ in CSE‐30C is consistent with its non‐uniform electric field and elevated anionic polarization near the electrode interfaces, which intensify local concentration gradients and encourage dendritic instability [47].

Temperature‐dependent ionic conductivity measurements substantiate this structure–transport correlation. Within the temperature range of 25–60°C, CSE‐30 consistently demonstrates the highest conductivity (8.04 × 10^−^^5^ S cm^−^^1^ at RT, 5.60 × 10^−^^3^ S cm^−^^1^ at 60°C), superior to both CSE‐30B and CSE‐30C (Figures S20, S21 and Table S3). The Arrhenius slope reveals the lowest activation energy for CSE‐30, indicating improved Li^+^ mobility facilitated by a percolated conduction network resulting from homogeneous additive dispersion and optimal polymer flexibility. Conversely, CSE‐30C experiences the discontinuous Li^+^ pathways and partial agglomeration of LLZO, resulting in sluggish conduction kinetics and thermal instability. These results suggest that the uniform tri‐layer configurations extends both the effective conduction cross‐section and interlayer connectivity, thereby ensuring cooperative transport across the entire membrane thickness.

To further elucidate the role of structural geometry, the impact of layer thickness distribution on electrochemical performance was systematically investigated. The superior performance exhibited by CSE‐30 originates from its structurally symmetric and compositionally balanced trilayer configuration, which creates a continuous and isotropic Li^+^ transport network. In contrast, CSE‐30B and CSE‐30C, with progressively thinned outer layers and thickened intermediate regions, revealed non‐uniform potential gradients and localized current accumulation at the Li interface, leading to higher interfacial resistance and the initiation of Li filament nucleation under high current densities.

This imbalance between the ionic flux and mechanical compliance induces early polarization and unstable cycling behavior. This effect becomes more pronounced in CSE‐30C, where excessively thin outer layers fail to provide sufficient elastic buffering and interfacial sealing, resulting in poor electrode wettability, discontinuous Li^+^ pathways, and increased charge‐transfer resistance. Simultaneously, the overly thick central additive‐rich layer introduces tortuous conduction pathways and enhances Li^+^ trapping within high‐binding‐energy coordination sites, thereby amplifying the concentration polarization and hindering effective ion percolation.

In contrast, the equal‐thickness configuration of CSE‐30 establishes a synergistic balance between interfacial stabilization and bulk ion transport, where the intermediate layer ensures regulated Li^+^‐ion coordination and flux distribution, and the outer PEO‐rich layers facilitated continuous conduction pathways and mechanical flexibility to accommodate interfacial stress. This integrated structure facilitates a uniform electric field distribution, minimizes concentration gradients, and promotes a homogeneous Li^+^ flux across the membrane. Consequently, the optimized architecture demonstrated the simultaneous enhancements in ionic conductivity, Li^+^ transference number, and long‐term cycling stability, revealing that its superiority originates from a structure–transport–mechanics coupling rather than geometric arbitrariness.

After identifying CSE‐30 (1:1:1) as the structurally optimized and electrochemically superior configuration, further investigations focused exclusively on this system to investigate its intrinsic transport dynamics, interfacial behavior, and full‐cell performance. The ionic conductivity of CSEs containing 10–40 wt% additive was systematically investigated across the temperature range of 25–60^°C^ (Figure 6b). All samples exhibited Arrhenius‐type behavior, confirming thermally activated Li^+^‐ion transport mediated by the segmental motion of the polymer. At every measured temperature, the CSEs systems demonstrated superior conductivity compared to pristine PEO, with performance strongly influenced by the ceramic additive content (Figures S22 and S23). At 25°C, the ionic conductivity of the reference PEO membrane was 2.20 × 10^−^^5^ S cm^−^^1^. The incorporation of PDA‐coated LLZO and PPP resulted in conductivities of 2.49 × 10^−^^5^, 5.38 × 10^−^^5^, and 3.31 × 10^−^^5^ S cm^−^^1^ for CSE‐10, CSE‐20, and CSE‐40, respectively (Figure 6c). Notably, CSE‐30 exhibited the highest room‐temperature conductivity (8.04 × 10^−^^5^ S cm^−^^1^), nearly four times that of the pristine PEO system. As the temperature increases to 60°C, the conductivity enhancement becomes more pronounced. The PEO electrolyte reaches 9.88 × 10^−^^4^ S cm^−^^1^, while CSE‐10, CSE‐20, and CSE‐40 achieve 1.80 × 10^−^^3^, 3.09 × 10^−^^3^, and 1.98 × 10^−^^3^ S cm^−^^1^ respectively, all remaining below the optimized CSE‐30 (5.60 × 10^−^^3^ S cm^−1^). The conductivity of CSE‐30 remained consistently the highest across all temperatures, reflecting an optimal balance between the ceramic content and matrix flexibility. The conductivity trend strongly correlates with the thermal and morphological data. The enhanced performance of CSE‐30 is attributed to its lower crystallinity and well‐dispersed PDA‐coated LLZO domains, which disrupt chain packing and create percolative ion transport pathways. The presence of PPP contributes to matrix flexibility, promoting segmental motion without a phase separation. At a higher ceramic content (CSE‐40), a modest decline in conductivity was observed despite a comparable room temperature value to that of CSE‐20, likely due to particle aggregation and reduced polymer chain dynamics. These results confirm that the rational tuning of the additive content and interfacial chemistry can significantly enhance Li^+^‐ion mobility in composite electrolytes by manipulating both the structural order and dynamic polymer behavior.

The temperature‐dependent conductivities were further analyzed using the Arrhenius relation to extract the activation energies (*E*_a_) for Li^+^ migration (Table S4). The pristine PEO electrolyte exhibited an activation energy of 0.97 eV, consistent with Li^+^ transport dominated by segmental motion through the semicrystalline polymer domains. Upon introducing the PDA@LLZO additive and PPP, *E*_a_ initially increased to 1.14 eV for CSE‐10, reflecting interfacial resistance and incomplete amorphization at low filler loading. The pronounced decrease to 1.06 eV for CSE‐20 and 1.07 eV for CSE‐30 indicates the establishment of an optimized conduction network, where hydrogen‐bonding interactions between PDA and PEO reduce the crystallinity and facilitate cooperative Li^+^ hopping through interconnected amorphous regions. At an excessive filler concentration (CSE‐40), *E*_a_ decreased to 1.01 eV, suggesting that although partial aggregation may occur, the system still maintains relatively accessible Li^+^ transport pathways compared to lower‐loading interfacial resistance‐dominated regimes. Overall, the activation energy trend mirrors the conductivity behavior, confirming that CSE‐30 achieves the most favorable balance between segmental mobility and interfacial conduction pathways, yielding the most efficient Li^+^‐ion transport across the temperature range.

To isolate the functional contribution of the triblock copolymer PPP, the ionic conductivity of CSE‐30 was compared with and without PPP incorporation over a similar temperature range (Figure S24). At all temperatures, the PPP‐containing membrane exhibited slightly higher conductivity, with values increasing from 8.04 × 10^−^^5^ S cm^−^^1^ at 25°C to 5.60 × 10^−^^3^ S cm^−^^1^ at 60°C (Figure 6d). The corresponding values for the PPP‐free membrane were 7.50 × 10^−^^5^ and 5.28 × 10^−^^3^ S cm^−^^1^, respectively. This enhancement, although modest, is systematic and highlights the cooperative role of PPP in facilitating Li^+^‐ion transport. Significantly, the observed improvement in the ionic conductivity cannot be attributed solely to mechanical reinforcement or crystallinity suppression effects typically associated with inert fillers. Instead, the PDA‐functionalized LLZO introduces chemically active interfaces that promote Li^+^ transport by modulating the local coordination environment and enabling the ion migration pathways. This distinction is critical, as inert oxide fillers generally serve as passive structural modifiers and do not significantly affect ion transport beyond their morphological impacts.

To further elucidate the influence of compositional design on selective Li^+^ conduction, the lithium transference number (t^+^) was evaluated for all CSEs using the Bruce–Vincent–Evans method, based on chronoamperometry and impedance spectra collected before and after DC polarization. The pristine PEO electrolyte exhibited a low t^+^ of 0.38 (Figure 6e), indicative of dominant anion mobility and poor cation selectivity, which are known to accelerate concentration polarization and interface degradation during long‐term cycling. Upon incorporation of the ceramic additive and block copolymer reinforcement, all CSEs samples exhibited an increased t^+^, reflecting the progressive suppression of TFSI^−^ transport and more favorable Li^+^ conduction. CSE‐10 and CSE‐20 achieved values of 0.49 and 0.52, respectively (Figures S25 and S26), whereas CSE‐30 delivered a substantially enhanced t^+^ of 0.81 (Figure 6f), approaching the regime of single‐ion conduction. The improvement in CSE‐30 is attributed to a highly synergistic network in which PDA‐functionalized LLZO particles act as surface‐tethered anion traps, whereas PPP imparts additional flexibility and coordinates Li^+^ ions through its ether‐rich PEG segments. This elevated *t* ^+^ value (0.81) further reinforces that charge transport is predominantly governed by Li^+^ ions, while TFSI^−^ mobility is effectively restricted at the macroscopic scale. This suppression of anion transport reduces their accumulation at the electrode interfaces, thereby reducing the concentration polarization and mitigating parasitic interfacial reactions during electrochemical operation.

Interestingly, the *t* ^+^ of CSE‐40 achieves a value of 0.41 (Figure S27), which shows a decline relative to CSE‐30, despite higher ceramic content. This behavior is consistent with filler overloading, where excessive LLZO aggregation disrupts the continuity of the polymer matrix, limits chain mobility, and reduces the segmental relaxation dynamics necessary for Li^+^ hopping. The marginal increase over pristine PEO highlights the competing interplay between anion immobilization and polymer chain disruption at high additive loadings. This “anion immobilization” does not originate from strong chemical binding of TFSI^−^ anion with the polymer components; rather, consistent with the DFT results suggesting weak dopamine–TFSI^−^ interactions, TFSI^−^ anions remain largely dissociated at the molecular level, however, experience restricted mobility at the macroscopic scale owing to the heterogeneous polymer–ceramic architecture and preferential Li^+^ coordination environment. Overall, the CSE‐30 composition emerged as the optimal formulation, balancing mechanical cohesion, ionic selectivity, and structural dynamics. The increase in *t* ^+^ observed across all the CSEs demonstrates the capability of engineered chemically active interfaces and polymer‐additive synergy to selectively improve Li^+^‐ion transport while diminishing electrochemical polarization effects in LMB systems, in contrast to conventional inert fillers that primarily serve as passive structural modifiers without efficiently decoupling cation and anion transport.

Mechanistically, Li^+^ migration in conventional PEO‐based SSE follows a thermally activated, segmental‐motion‐assisted hopping mechanism in which ether oxygens transiently coordinate Li^+^ ions. This process is strongly governed by polymer chain mobility; crystalline domains restrict motion and act as insulating barriers, while amorphous regions, though favorable for transport, occupy only a limited fraction of matrix. Moreover, strong Li^+^–TFSI^−^ association leads to ion pairing and cluster formation, reducing free‐carrier density and inducing concentration polarization, which promotes uneven Li^+^ flux and dendritic growth during cycling.

Incorporating PDA‐functionalized LLZO additive and triblock copolymer PPP into the PEO matrix fundamentally redefines this transport landscape. PDA@LLZO serves both as an ionically conductive additive and an interfacial coupling agent. The hydroxyl, amine, and catechol groups of PDA form hydrogen bonds with PEO chains, ensuring uniform additive dispersion and minimizing phase segregation. Concurrently, the polar PDA surface selectively interacts with TFSI^−^ anions, immobilizing them through dipole and π–π interactions, thereby increasing free Li^+^ concentration and enhancing the transference number. LLZO contributes a rigid, superionic framework that introduces percolating Li^+^ pathways decoupled from polymer dynamics, reducing conduction tortuosity and enabling multi‐directional transport even at moderate temperatures.

The PPP copolymer further reinforces this hybrid network by combining mechanical elasticity with ionic functionality. Its PEG‐rich segments provide additional Li^+^ coordination sites, while the flexible PPG blocks impart mechanical compliance and dissipate local stress. Acting as a compatibilizer, PPP bridges the stiff LLZO domains and the flexible PEO matrix, ensuring cohesive interfaces and maintaining ionic continuity under repeated plating and stripping. Collectively, these interactions yield a dual‐phase conduction mechanism in which Li^+^ transport occurs through fast ceramic channels and segmental‐motion‐assisted hopping across amorphous polymer regions. This hierarchically interconnected network transforms the composite from a diffusion‐limited medium into a percolating Li^+^ conductor with high ionic conductivity, elevated transference number, and reduced interfacial resistance.

At the macroscopic scale, the tri‐layer CSE‐30 architecture also establishes a complementary soft–hard–soft configuration that orchestrates Li^+^ flux uniformly across interfaces. The outer PEO‐rich layers provide soft, conformal ionic contact with Li metal, while the inner PDA@LLZO‐PPP layer forms a rigid, ionically conductive backbone that resists dendritic penetration. This graded mechanical profile balances interfacial adaptability with structural rigidity, enabling stress dissipation, homogeneous Li^+^ diffusion, and long‐term cycling stability. In essence, the tri‐layer CSE‐30 embodies a hierarchically engineered electrolyte in which molecular‐level interactions and architectural design converge to deliver fast Li^+^ transport, interfacial integrity, and dendrite‐resistant operation, offering a generalizable framework for next‐generation polymer–ceramic hybrid electrolytes.

To evaluate the anodic stability of the developed CSEs, linear sweep voltammetry (LSV) was conducted at 60°C, and the results are presented in Figure 6g. The measurements were executed using a Li/CSE/stainless steel (SS) configuration, where the SS serves as an inert blocking electrode. The pristine PEO electrolyte exhibited an oxidation onset at approximately 4.5 V vs Li/Li^+^, beyond which a steep increase in current was observed, signifying the electrochemical degradation of the polymer backbone under oxidative conditions. In contrast, CSE‐30 demonstrated a significantly delayed oxidation onset at 5.5 V vs Li/Li^+^, accompanied by a notably lower current response across the scanned potential range. The extended electrochemical stability window of CSE‐30 is attributed to the synergistic interplay between its constituents. The PDA@LLZO particles, uniformly dispersed within the PEO matrix, serve not only as electrochemically inert scaffolds but also facilitate strong hydrogen‐bonding interactions with the PEO chains. These interactions limit the mobility of the ether segments and suppress the high‐energy chain conformations that are susceptible to oxidation. Moreover, the electron‐donating catechol moieties of PDA form a passivating interphase that kinetically hinders electron transfer at high potentials. Simultaneously, PPP acts as a flexible entanglement network that reinforces the mechanical stability of the polymer phase and mitigates localized dielectric breakdown under high‐voltage stress. Notably, the observed delayed oxidation behavior reflects kinetically suppressed interfacial oxidation reactions enabled by the tri‐layer structure, which regulates the ion distribution and diminishes localized polarization when high potentials are applied. The suppressed anodic current in CSE‐30 further underscores the reduction in parasitic faradaic processes, indicating a stable electrochemical environment at voltages suitable for advanced cathode chemistries [48]. These findings establish that rational design through the incorporation of functionalized ceramic additives and soft block copolymers can substantially enhance the anodic tolerance of PEO‐based SSEs, thereby enabling their integration into high‐voltage solid‐state LMB systems.

Cyclic voltammetry was employed to study the redox characteristics and Li plating/stripping dynamics of both the PEO electrolyte and CSE‐30 using a Li/electrolyte/LFP setup, allowing for the investigation of the interfacial electrochemical behavior under practical cathode conditions. The PEO electrolyte exhibited broad and weakly defined redox characteristics, with oxidation and reduction peaks at 3.67 and 3.20 V, respectively (Figure 6h). The anodic and cathodic peak currents consistently diminished over the cycles, from +0.957 to +0.820 mA and from −0.942 to −0.778 mA, respectively, exposing no sign of stabilization, indicating ongoing interfacial degradation and poor electrochemical reversibility. The marginal narrowing of the peak separation from 0.480 to 0.411 V (Δ = 0.069 V) across three cycles further indicates the persistently sluggish charge‐transfer kinetics, which are characteristic of highly crystalline, ionically resistive frameworks with limited Li^+^ mobility.

In contrast, CSE‐30 demonstrated a significantly superior CV response within a similar voltage window, with sharper, more symmetric, and well‐defined redox peaks (Figure 6i). The anodic–cathodic peak separation narrowed markedly from 0.518 V (Cycle 1) to 0.373 V (Cycle 3), a reduction of 0.145 V, which is more than twice that of PEO, directly indicating the gradually reduced interfacial polarization, accelerated interfacial charge transfer kinetics, and improved electrochemical reversibility. The progressive stabilization of anodic peak currents from +0.876 to +0.685 mA and cathodic peak currents from −0.832 to −0.637 mA, in contrast to the continuous decay in PEO, indicates a more uniform Li stripping/plating at the anode, reflecting progressive stabilization of the Li metal interface consistent with SEI maturation. Simultaneously, the stabilized cathodic response reveals reversible lithiation/delithiation at the LFP cathode, aligns with gradual CEI formation. Collectively, these characteristics confirm enhanced Li^+^ transport and more effective electrode wetting within an ionically and mechanically adaptive electrolyte interface.

The simultaneous narrowing of the peak separation and sharpening of redox profiles collectively reinforced the reduced polarization, faster interfacial charge transfer, and progressive stabilization of both electrode interfaces. These improvements originate from the trilayer structure of CSE‐30, in which the LLZO phase from the inner layer contributes to high intrinsic Li^+^ conductivity and encourages lateral ion diffusion, whereas the PDA coating ensures interfacial compatibility with the PEO matrix through hydrogen bonding and dipole interactions. This functional coating reduces the crystallinity of PEO and supports a homogeneous Li^+^‐ion flux. Simultaneously, PPP introduces elastic mechanical reinforcement that maintains electrolyte integrity during Li cycling and buffers against volume‐change‐induced stress. The progressive stabilization of the current response in CSE‐30 is consistent with the gradual establishment of a stable CEI at the cathode interface, which enables reversible lithiation/delithiation without promoting parasitic side reactions. The symmetric and reproducible CV curves further reinforce the electrochemical stability of both electrode interfaces, which is consistent with the reduced interfacial resistance and sustained redox activity across repeated cycles. These results collectively reveal that, despite the intrinsic instability of PEO, the trilayer architecture facilitates kinetically stabilized electrode interfaces, thereby suppressing degradation under practical operating conditions.

The long‐term galvanostatic Li plating/stripping behavior of symmetric Li‖Li cells assembled with pristine PEO and a series of CSEs (CSE‐10, CSE‐20, CSE‐30, and CSE‐40) was systematically investigated at a current density of 0.2 mA cm^−^^2^ and 60°C. The voltage‐time profiles distinctly reveal the critical role of interfacial stability between the Li‐metal anode and electrolyte in dictating cycling durability and resistance to short‐circuiting.

The pristine PEO‐based cell showed a rapidly increasing overpotential and unstable voltage oscillations within the first 200 h, culminating in short‐circuit failure (Figure 6j). These fluctuations reflect poor interfacial contact, high interfacial resistance, and uncontrolled Li dendrite growth through a mechanically soft PEO matrix. The broadening of the voltage hysteresis with time signifies progressive interface degradation, which is associated with localized current concentration and non‐uniform Li deposition. In contrast, the CSE‐30 system exhibited exceptional interfacial robustness, maintaining a symmetric and stable voltage profile (±75 mV) for 1000 h of uninterrupted cycling. The enhanced stability originates from the synergistic interplay of PDA@LLZO and flexible PPP, where LLZO contributes to fast Li^+^ conduction and provides a rigid backbone for ion transport, PDA@LLZO ensures uniform Li flux and strong interfacial adhesion through hydrogen bonding and Lewis acid‐base interactions, and PPP imparts mechanical elasticity that preserves intimate Li contact and accommodates volume fluctuations without fracture or delamination. This stable behavior is attributed to Li^+^‐dominated transport, which suppresses anion accumulation at the interface, thereby minimizing the concentration polarization and enabling uniform Li plating/stripping over prolonged cycling.

Electrolytes with suboptimal additive contents (CSE‐10 and CSE‐20) initially showed stable cycling but gradually accumulated overpotential beyond 350 h (Figure S28) and 580 h (Figure S29), respectively. This suggests incomplete passivation of the Li‐electrolyte interface, where the partial suppression of dendrites delays but does not entirely prevent short‐circuit events. Conversely, CSE‐40 (Figure S30), with excessive ceramic loading, displayed a higher interfacial resistance and an earlier failure at 190 h, likely due to reduced ionic percolation pathways and increased interphase rigidity, leading to stress‐induced interfacial breakdown.

To validate the robustness of the Li electrolyte interface (SEI) under dynamic electrochemical conditions, rate capability testing of Li‖Li symmetric cells was performed by progressively increasing and decreasing the current density from 0.1 to 1.0 mA cm^−^^2^. The pristine PEO‐based electrolyte exhibited pronounced voltage noise and rapid loss of interfacial stability beyond 0.3 mA cm^−^^2^ (Figure 6k), with severe polarization fluctuations and short‐circuit‐like behavior emerging prominently at 0.5 and 1.0 mA cm^−^^2^. These instabilities reflect poor Li^+^ flux accommodation, limited ionic conductivity, and mechanically induced interfacial disruption under an elevated current stress. The inability to re‐establish stable voltage profiles upon returning to 0.1 mA cm^−^^2^ further underscores irreversible interfacial degradation and dendrite‐induced breakdown.

In contrast, the CSE‐30 electrolyte exhibited outstanding electrochemical flexibility across all tested current densities (Figure 6l). The voltage profiles remained symmetric, smooth, and narrowly confined within ±0.15 V even at 1.0 mA cm^−^^2^, without evidence of voltage spiking or morphological instability. Upon returning to 0.1 mA cm^−^^2^, the CSE‐30 cell rapidly recovered its original polarization, indicating highly reversible Li plating/stripping and the absence of structural fatigue at the interface. These results underscore the critical importance of a balanced electrolyte design that synergistically integrates mechanical robustness, interfacial compatibility, and ionic homogeneity. The CSE‐30 architecture exemplifies this balance, uniquely combining polymer–ceramic interactions to effectively suppress dendrite nucleation and propagation. Its demonstrated ability to sustain prolonged cycling without voltage instability or polarization drift confirms that optimal additive chemistry and polymer dynamics are essential for stabilizing Li electrolyte interfaces under practical current densities. Moreover, CSE‐30's resilience extends to accommodating rapid Li^+^ flux, preserving interfacial integrity even under high‐rate operation, thereby realizing the key performance requirements for high‐energy solid‐state LMBs. Collectively, these findings position CSE‐30 as a benchmark system that highlights the pivotal interplay between material composition and interfacial engineering in advancing dendrite‐free, durable SSEs.

**FIGURE 7:**
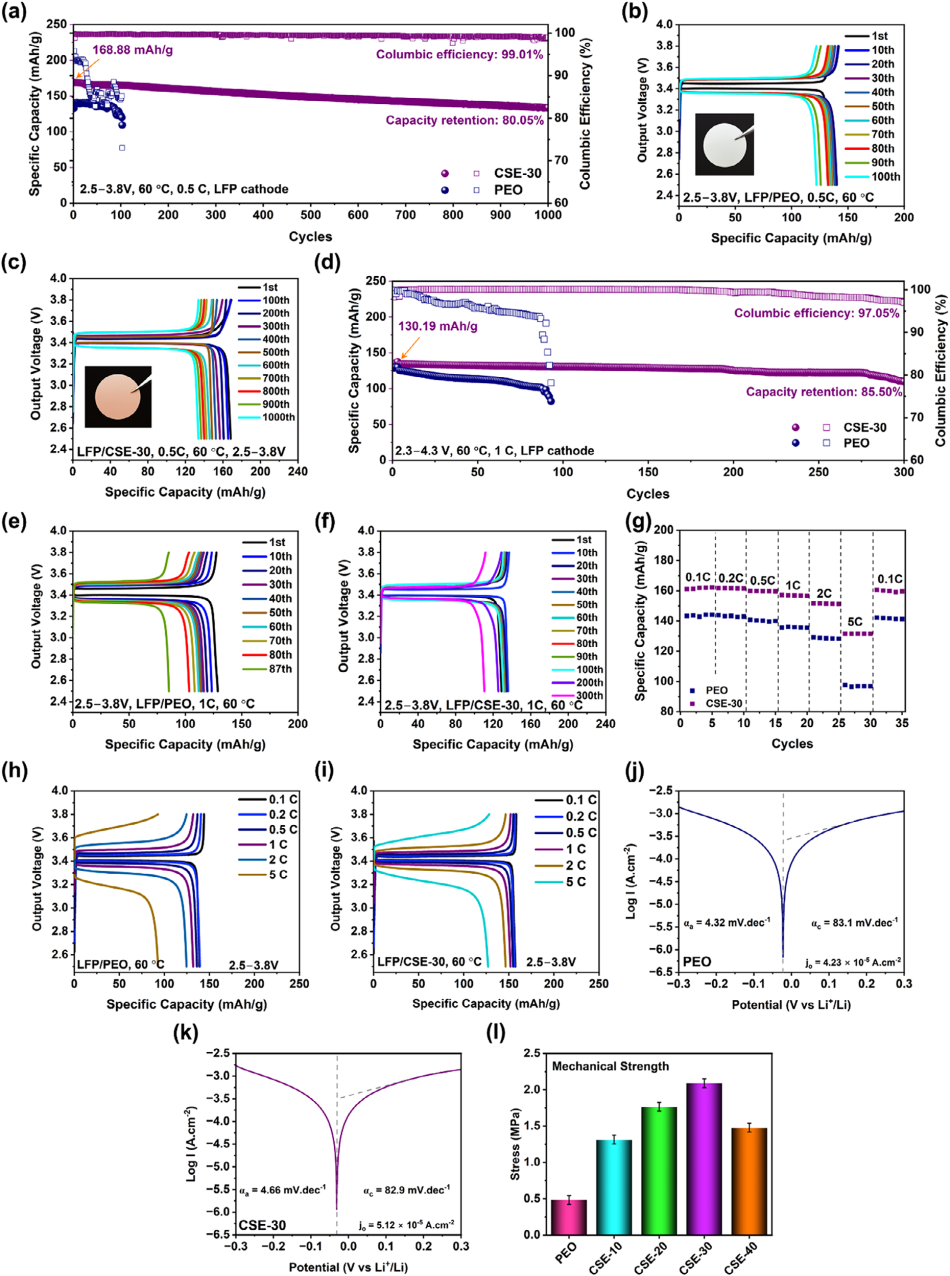
Electrochemical performance and mechanical characteristics of PEO and CSEs. (a) Cycling performance at 0.5C shows that CSE‐30 delivers stable capacity retention (133.66 mAh g^−^^1^ at 1000 cycles) and high CE (99%), while the PEO suffers rapid degradation and complete failure before 200 cycles. Voltage profiles illustrate severe polarization and distorted plateaus for (b) PEO by 100^th^ cycle, in contrast to (c) CSE‐30 cell, which maintains sharp and overlapping charge–discharge plateaus up to 1000^th^ cycle. (d) Long‐term cycling at 1C reveals that CSE‐30 maintains high specific capacity (110.08 mAh g^−^^1^ after 300 cycles with 97% CE), while PEO suffers rapid capacity fade and fails before 100 cycles. Voltage profiles over multiple cycles demonstrate stable and overlapping charge‐discharge plateaus in (e) PEO, in stark contrast to (f) CSE‐30 cell, which shows increasing polarization and distorted voltage profiles. (g) Rate capability comparison from 0.1C to 5C and back to 0.1C highlights superior capacity retention and rate reversibility in CSE‐30, whereas the PEO suffers irreversible capacity loss at high rates. (h) Voltage profile of PEO at various C‐rates. (i) Voltage profiles of CSE‐30 at various C‐rates exhibit narrow overpotentials and preserved voltage plateaus, confirming its robust interfacial kinetics and structural stability under dynamic conditions. (j) Tafel plot of pristine PEO shows low exchange current density (*j*_0_ = 4.23 × 10^−^^5^ A cm^−^^2^) and sluggish charge‐transfer kinetics. (k) Tafel plot of CSE‐30 exhibits higher *j*_0_ (5.12 × 10^−^^5^ A cm^−^^2^) and improved charge‐transfer behavior. (l) Mechanical strength of PEO and CSEs, with CSE‐30 shows the highest stress (2.09 MPa), confirming optimal reinforcement and dendrite resistance.

To validate the long‐term cycling stability and interfacial integrity of the CSEs under realistic full‐cell operating conditions, LFP‖Li full cells were assembled using CSE‐30 and pristine PEO electrolytes and evaluated at 0.5C and 60°C. As shown in Figure 7a, the CSE‐30‐based full cell demonstrated remarkable electrochemical durability over 1000 cycles, delivering an initial specific discharge capacity of 168.92 mAh g^−^^1^ with a high Coulombic efficiency (CE) of 97.9%, corresponding to a minimal Li loss of only 2.10%, which confirms highly stable interfacial chemistry and negligible irreversible Li consumption (Figure S31). Even after 200 cycles (Figure S32), the cell retained a capacity of 160.66 mAh g^−^^1^ with negligible decay and a consistent CE of 99.65%, eventually maintaining 133.66 mAh g^−^^1^ at the 1000^th^ cycle with a CE of 99%. This exceptionally low first‐cycle loss validates the efficient electrode‐electrolyte contact and the ability of the PDA@LLZO–PPP framework to suppress parasitic side reactions typically observed in solid‐state interfaces. Importantly, this stable long‐term cycling performance and Coulombic efficiency further reveal that the tri‐layer architecture efficiently regulates and stabilizes both the Li metal and cathode interfaces, despite direct contact with PEO outer layers. This enhanced performance originates from kinetically regulated interfacial processes, where the inner PDA@LLZO/PPP layer maintains a uniform Li^+^ flux, suppresses localized polarization, and mitigates parasitic oxidative reactions at the cathode, thereby enabling stable CEI formation during prolonged cycling.

In contrast, the PEO‐based cell exhibited pronounced capacity fading and CE deterioration. The specific capacity rapidly declined from 132.64 mAh g^−^^1^ (CE of 95.34%) in the first cycle to 109.30 mAh g^−^^1^ with only 72.98% CE by the 103^rd^ cycle. Complete electrochemical failure occurred before the 200th cycle, highlighting the severe interfacial degradation and internal impedance growth. These results were further confirmed by the charge–discharge voltage profiles (Figure 7b). The CSE‐30‐based full cell exhibited minimal polarization and highly stable voltage plateaus up to 1000 cycles, demonstrating suppressed dendrite growth and sustained electrolyte–electrode contact. In contrast, the PEO‐based cell exhibited progressive voltage polarization and flattened plateaus by the 100^th^ cycle (Figure 7c), indicating poor Li^+^‐ion transport and interfacial instability. Hence, the superior capacity retention, near‐unity CE, and stable electrochemical signatures of CSE‐30 confirm the structural robustness and interfacial compatibility of the PDA@LLZO‐PPP‐reinforced CSE. These results emphasize the promise of rationally engineered CSEs for achieving long cycling stability and safety in practical LMB architectures.

Building upon the prolonged cycling performance observed at 0.5C, the full‐cell behavior was further explored at a higher current density of 1C to evaluate the interfacial resilience and rate adaptability of CSE‐30 in more challenging environments. As shown in Figure 7d, the CSE‐30‐based Li‖LFP full cell delivered a stable initial discharge capacity of 128.80 mAh g^−^^1^ with a CE of 98.34%, gradually stabilizing to 130.98 mAh g^−^^1^ at the 93^rd^ cycle and maintaining 110.08 mAh g^−^^1^ with 97.05% CE even after 300 cycles. This long‐term performance evidences the potential of the electrolyte to suppress interfacial degradation and facilitate efficient Li^+^ transport even at higher C‐rates. Conversely, the PEO‐based cell showed rapid electrochemical deterioration, with the capacity reducing to 82.36 mAh g^−^^1^ and the CE falling to 78.15% by the 93^rd^ cycle, followed by complete failure before reaching 100 cycles, highlighting its instability at high current densities. The corresponding voltage profiles (Figure 7e,f) further reinforce the superior charge–discharge reversibility of the CSE‐30 system, which retained flat and overlapping voltage plateaus up to 300 cycles, whereas the PEO cell suffered severe polarization, distorted plateaus, and early voltage breakdown, consistent with Li dendrite growth and unstable SEI formation.

To assess the rate adaptability and structural resilience, the LFP‐based full cells were examined under a stepped current protocol ranging from 0.1 to 5C and then returned to 0.1C (Figure 7g). The CSE‐30‐based cell consistently delivered high and stable capacities at all rates, achieving 162 mAh g^−^^1^ at 0.1C, 161 mAh g^−^^1^ at 0.2C, 159 mAh g^−^^1^ at 0.5C, 156 mAh g^−^^1^ at 1C, 151 mAh g^−^^1^ at 2C, and 136 mAh g^−^^1^ at 5C. Upon returning to 0.1C, the capacity recovered to 159 mAh g^−^^1^, demonstrating excellent rate reversibility and minimal structural fatigue under dynamic conditions. In comparison, the PEO‐based cell exhibited significantly lower capacities of 145 mAh g^−^^1^ at 0.1C, 143 mAh g^−^^1^ at 0.2C, 140 mAh g^−^^1^ at 0.5C, 135 mAh g^−^^1^ at 1C, 128 mAh g^−^^1^ at 2C, and only 97 mAh g^−^^1^ at 5C. Notably, its recovered capacity at 0.1C was 141 mAh g^−^^1^, which remained below the initial value, indicating irreversible degradation and poor interfacial recovery at high rates. The PEO cell showed increasing overpotentials and distorted profiles with higher currents (Figure 7h), suggesting sluggish ionic transport and interfacial instability. Conversely, the voltage profiles for the CSE‐30 cell exhibited narrow polarization gaps and well‐defined charge–discharge plateaus across all C‐rates (Figure 7i), underscoring the robust interfacial kinetics and superficial Li^+^ mobility. Overall, these results highlight the rate‐resilient structure of the CSE‐30 electrolyte, which is capable of sustaining high‐power operation without compromising electrochemical integrity, a crucial benchmark for advancing the next‐generation LMBs.

To explore the interfacial charge‐transfer kinetics quantitatively at electrode‐electrolyte interface, Tafel polarization measurements were analyzed using Butler–Volmer formalism. The polarization curves of both the pristine PEO and the optimized tri‐layer CSE‐30 electrolytes exhibited distinct anodic and cathodic branches, confirming activation‐controlled electrochemical behavior. For pristine PEO electrolyte (Figure 7j), the linear extrapolation of Tafel regions, yielded anodic and cathodic slopes (α_a_ and α_c_) as 4.32 and 83.1 mV dec^−^^1^, respectively. The equilibrium (corrosion) potential (*E*_corr_) was identified at −0.01709 V vs Li^+^/Li, while the corresponding corrosion (exchange) current density (*I*_corr_ ≈ *j*_0_) was calculated as 4.23 × 10^−^^5^ A cm^−^^2^. For the optimized CSE‐30 electrolyte (Figure 7k), the anodic and cathodic slopes were significantly reduced to 4.66 and 82.9 mV dec^−^^1^, respectively. The equilibrium potential (*E*_corr_) was measured at −0.0255 V vs Li^+^/Li, and the corresponding corrosion (exchange) current density (*I*_corr_ ≈ j_0_) was calculated as 5.12 × 10^−^^5^ A cm^−^^2^. These parameters provide the fundamental descriptors required to reconstruct the Butler–Volmer kinetics of Li^+^/Li charge transfer across the electrolyte‐metal interface.

The generalized Butler–Volmer expression governing interfacial kinetics is given by:

$$j=j_{o}\;\left[\exp\left(\frac{\alpha_{a}nF\eta }{RT}\right)-\exp\left(-\frac{\alpha_{c}nF\eta }{RT}\right)\right]$$

where *j_0_* denotes the exchange current density, α_a_ and α_c_ are the anodic and cathodic charge transfer coefficients, *η = E – E*_eq_ is the overpotential, (*n*) is the number of transferred electrons, *F* is the Faraday constant (96485 C mol^−^^1^), *R* is the gas constant (8.314 J mol^−^^1^ K^−^^1^), and *T* is the absolute temperature (298 K).

At sufficiently high |*η*|, the equation simplifies into the classical Tafel form:

$$\eta =\left(\frac{2.303RT}{anF}\right)\;\mathrm{lo}g_{10}\left(\frac{j}{j_{o}}\right)$$

With a slope

$$b=\frac{2.303RT}{anF}$$

Using this expression, the charge‐transfer coefficients were obtained as:

$$\alpha_{a}=\frac{0.0591}{b_{a}}$$

and

$$\alpha_{c}=\frac{0.0591}{b_{c}}$$

Substituting the experimental slopes gives for the PEO electrolyte α_*a*_ =  5.43 and α_*c*_ =  0.23, while for the CSE‐30 electrolyte α_*a*_ =  12.67 and α_*c*_ =  0.71. The unusually large anodic coefficient (α_a_ > 1) values in both systems indicate non‐ideal anodic behavior dominated by partial ohmic and diffusion polarization, whereas the realistic cathodic coefficients (α_c_ ≈ 0.7) signify a kinetically controlled Li^+^ reduction process governed by electron‐transfer activation barriers. Consequently, the cathodic branch is considered the kinetically meaningful regime for describing the Butler–Volmer behavior in both electrolyte systems.

At equilibrium, the exchange current density (PEO; *j*_0 =_ 4.23 × 10^−^^5^ A cm^−^^2^ and CSE‐30; *j*_0 =_ 5.12 × 10^−^^5^ A cm^−^^2^) quantifies the intrinsic Li^+^ ⇌ Li^0^ interfacial conversion rate. The full Butler–Volmer expressions for both electrolytes can be expressed as:

For the pristine PEO electrolyte:

$$j=4.23\;\times 10^{-5}\;\left[\exp\left(\frac{0.71\;\times 96485\;\times \;\eta }{8.314\;\times 298}\right)-\exp\left(-\frac{0.5\;\times 96485\;\times \eta }{8.314\;\times 298}\right)\right]$$

and for the CSE‐30 electrolyte:

$$j=5.12\;\times 10^{-5}\;\left[\exp\left(\frac{0.71\;\times 96485\;\times \;\eta }{8.314\;\times 298}\right)-\exp\left(-\frac{0.5\;\times 96485\;\times \eta }{8.314\;\times 298}\right)\right]$$

The reconstructed j(η) profiles closely matched the experimental Tafel data within the low‐to‐moderate overpotential regions, confirming activation‐controlled kinetics for both electrolyte systems. The modest exchange current density and high cathodic Tafel slope (≈ 83 mV dec^−^^1^) in PEO system signify a sluggish Li^+^‐transfer process across the PEO interface, attributed to the high crystallinity and limited segmental mobility of PEO chains with high interfacial resistance. The asymmetric kinetic parameters (*α*_a_ ≫ *α*_c_) further suggest an uneven interfacial energy barrier, leading to polarization during Li stripping.

However, the lower cathodic Tafel slope and higher exchange current density of CSE‐30 demonstrate substantially improved Li^+^ interfacial transport compared to pristine PEO. Mechanistically, this enhancement is attributed to synergistic interfacial and structural effects [49]. PDA‐engineered LLZO particles introduce abundant surface –─OH, –─NH and catechol groups that form hydrogen bonds with ether‐oxygens in PEO, disrupting its crystallinity and establishing continuous, fast Li^+^ conduction pathways. PDA coating establishes robust chemical coupling between the inorganic filler and the polymer matrix. Simultaneously, the PPP copolymer reinforces the middle layer to contribute to mechanical flexibility by providing an elastic polymeric framework within the hybrid layer. The flexible PPG segments of PPP dissipate interfacial stress and accommodate volume fluctuations during Li plating and stripping. Whereas the PEG‐rich ends of PPP physically entangle with PEO to enhance local segmental motion, by improving interfacial contact and reducing the interfacial stress and acting as a compliant mechanical “bridge” between rigid PDA‐engineered LLZO particles and the PEO matrix. This dual chemical–mechanical coupling produces a self‐adaptive interphase to strengthen the cohesive integrity, mitigate interfacial void formation and delamination, and preserve the intimate contact between the filler and matrix under electrochemical operation. As a result, these modifications lower the interfacial energy barrier for Li^+^ desolvation and electron transfer, enhance Li^+^ mobility and electronic coupling at the metal‐electrolyte interface, leading to faster charge transfer (higher *j*_0_) and reduced polarization losses without altering the fundamental charge‐transfer mechanism [50].

Hence, the kinetic comparison between pristine PEO and CSE‐30 reveals a transition from slow, polarization‐dominated Li^+^ exchange to fast, activation‐controlled interfacial charge transfer. The optimized CSE‐30 electrolyte exhibits a more negative *E*_corr_, lower Tafel slope, and a higher exchange current density, confirming its superior interfacial stability and enhanced Li^+^ transport efficiency. These findings reveal hat interfacial engineering through PDA‐modified LLZO incorporation, PPP reinforcement, and tri‐layer structural integration effectively tailors the Butler–Volmer kinetics, rendering CSE‐30 a kinetically favorable, mechanically resilient, and dendrite‐resistant solid‐state electrolyte for high‐performance LMBs [51].

To rigorously evaluate the mechanical stability of the SPE and CSEs, uniaxial tensile tests were conducted under ambient conditions using a universal testing machine (UTM), with three independent samples measured for composition (*n* = 3). The resulting stress–strain behavior is summarized in Figure 7l, with additional data provided in Figures S33–S35. All reported tensile strength values represent the mean ± standard deviation (SD) of three independent measurements.

The pristine PEO‐based electrolyte exhibited a low maximum tensile stress of 0.48 ± 0.006 MPa together with a very high elongation at break of around 1400%, highlighting its intrinsically soft and highly ductile mechanical character. Linear regression analysis applied to the initial elastic region of the stress–strain curve yielded a slope of 2.05 × 10^−^^4^ MPa/% (*R* ^2^ = 0.538, Pearson's *r* = 0.734), consistent with PEO's low modulus and high chain mobility. Although such compliance is beneficial for flexibility and processability, it also indicates a limited ability to resist mechanical deformation, which can be detrimental under the stress conditions encountered during Li‐metal cycling.

Upon incorporation of ceramic fillers and the elastomeric PPP component, a clear enhancement in mechanical strength was observed while maintaining substantial deformability. CSE‐10 displayed an increased tensile strength of 1.31 ± 0.006 MPa with a linear fit slope of 5.67 × 10^−^^4^ MPa/% (*R* ^2^ = 0.789, Pearson's *r* = 0.888), reflecting the initial contribution of the inorganic domains to load bearing within the polymer matrix.

Further improvement was achieved in CSE‐20, which reached a maximum stress of 1.76 ± 0.006 MPa and a slope of 6.94 × 10^−^^4^ MPa/% (*R* ^2^ = 0.539, Pearson's *r* = 0.734), while still sustaining a large elongation at break of nearly 1600%, suggesting the formation of a mechanically reinforced yet highly stretchable composite network.

The most favorable mechanical performance was obtained for CSE‐30, which exhibited the highest tensile strength of 2.09 ± 0.010 MPa combined with an elongation exceeding 1700%, and the highest linear fit slope of 9.17 × 10^−^^4^ MPa/% (*R* ^2^ = 0.908, Pearson's *r* = 0.953). This unique combination of strength and ductility indicates a tough–elastic response arising from a well‐balanced microstructure. In this configuration, the uniformly distributed PDA@LLZO domains act as effective stress‐transfer points, while the PPP‐containing polymer network accommodates large deformations through efficient energy dissipation. Such synergistic architecture is particularly beneficial for suppressing localized stress accumulation and mitigating mechanically driven dendrite penetration in LMBs.

When the ceramic loading was further increased, CSE‐40 showed a reduction in tensile strength to 1.48 ± 0.010 MPa accompanied by a decrease in elongation at break, with a slope of 6.29 × 10^−^^4^ MPa/% (*R* ^2^ = 0.872, Pearson's *r* = 0.934). This behavior points to the onset of mechanical embrittlement at excessive filler content, likely caused by disruption of polymer chain continuity and the emergence of filler agglomerates that serve as stress concentration sites. To clarify the specific role of PPP, a control CSE‐30 sample was prepared without incorporating the block copolymer. In the absence of PPP, the electrolyte exhibited a tensile strength of 1.82 MPa and an elongation at break of around 1533%, in clear contrast to the 2.09 ± 0.010 MPa strength and >1700% strain achieved for CSE‐30 containing PPP. This comparison demonstrates that PPP plays a critical role in preserving ductility while enhancing mechanical strength, likely by redistributing mechanical strain, bridging rigid ceramic domains, and delaying crack initiation and propagation.

Overall, the mechanical results reveal a clear structure–property relationship, in which mechanical reinforcement improves progressively with increasing ceramic content up to an optimal loading of 30 wt%, beyond which further addition leads to diminishing returns and partial embrittlement. The cooperative effect between PDA@LLZO and PPP enables the construction of a mechanically robust yet highly deformable electrolyte, exemplified by the optimized CSE‐30 composition. Importantly, the PDA@LLZO component does not function only as a passive mechanical filler but also as a multifunctional phase that simultaneously modulates ion coordination, transport selectivity, and interfacial dynamics. This multifunctionality distinguishes the present system from conventional composite electrolyte systems employing inert fillers, where enhancements are typically restricted to mechanical reinforcement and partial crystallinity suppression without significant regulation of the ion transport pathways. This balanced mechanical profile is essential for maintaining interfacial integrity, resisting deformation induced by lithium plating and stripping, and supporting the long‐term stability and safety of solid‐state LMBs.

**FIGURE 8:**
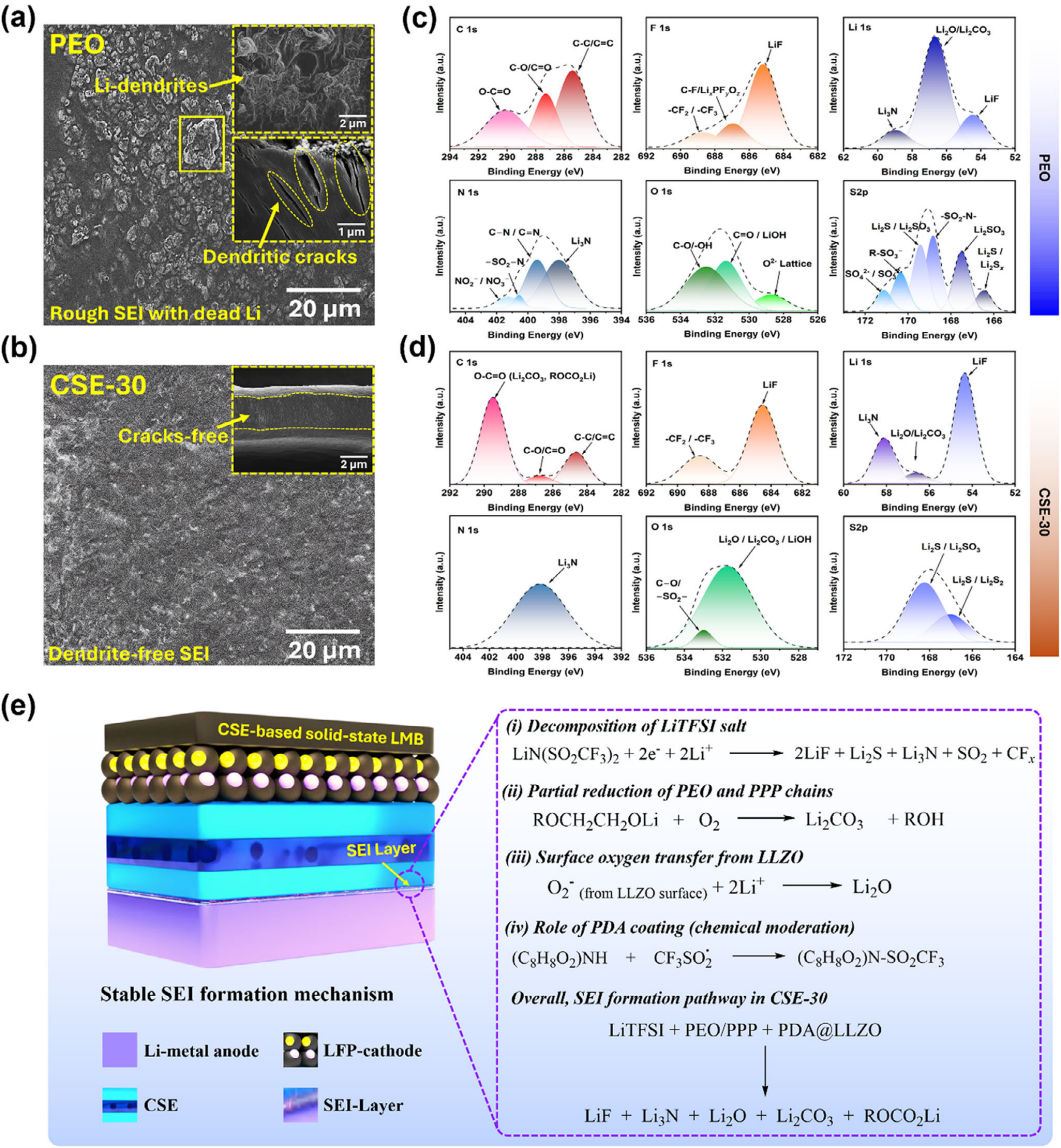
Comparative surface and interfacial characterization of cycled Li anodes paired with PEO and CSE‐30 electrolytes, illustrating morphological and compositional differences in the SEI and cross‐sectional integrity after charge–discharge cycling. (a) Top‐view SEM reveals a severely disrupted and dendrite‐rich SEI on the Li anode with PEO. Upper inset: Top‐view SEM image showing rough SEI features. Lower inset: Cross‐sectional SEM image of the Li|PEO interface showing dendritic cracks and SEI disruption, indicative of Li‐dendrite penetration and interfacial failure. (b) SEM image of Li anode cycled with CSE‐30 which maintains a smooth and dendrite‐free interfacial morphology. Inset: Cross‐sectional SEM image of Li|CSE‐30 interface showing dense, crack‐free morphology, confirming superior interfacial structural stability and mechanical robustness. (c) XPS spectra (C 1s, F 1s, Li 1s, N 1s, O 1s, S 2p) of the PEO‐based SEI revealing heterogeneous inorganic/organic components such as Li_2_CO_3_, LiF, and ROCO_2_Li. (d) XPS spectra of CSE‐30 SEI showing enriched LiF and Li_2_O species indicative of a more uniform and stable interphase. (e) Schematic illustration of proposed interfacial reactions on Li‐metal anodes in pristine PEO and CSE‐30 electrolytes, highlighting the formation of LiF, Li_3_N, Li_2_O, and Li_2_CO_3_ based composite SEI via controlled LiTFSI and polymer reduction reactions.

To substantiate the electrochemical observations from the charge–discharge cycling of the full cell, ex situ SEM imaging and elemental XPS analysis were conducted to examine the morphology of the lithium metal surface and the solid electrolyte interphase (SEI) after cycling with pristine PEO and optimized CSE‐30 electrolytes (Figure 8). The SEM micrographs (Figure 8a) of the Li‐metal anode surface cycled with the pristine PEO electrolyte revealed a severely degraded and uneven SEI morphology. The surface is covered with a rough, porous layer densely populated by nodular deposits and isolated clusters of electrochemically inactive “dead” lithium, reflecting uncontrolled interfacial reactions. High‐magnification imaging revealed a dense network of filamentous lithium dendrites, which is clear evidence of unstable Li deposition and localized current hotspots. Such dendritic protrusions and uneven SEI reconstruction signify a mechanically fragile and chemically heterogeneous interface that is prone to continuous rupture and repair during cycling [52]. The intrinsically weak mechanical strength of the PEO matrix fails to suppress localized stress accumulation, enabling spontaneous Li nucleation and the emergence of mossy, needle‐like dendritic growth. This morphological instability directly mirrors the electrochemical behavior observed earlier: high overpotentials, pronounced voltage hysteresis, and eventual short‐circuiting, confirming that the pristine PEO system cannot sustain stable Li plating and stripping. Cross‐sectional SEM images provide deeper mechanistic insights into the interfacial evolution of the Li‐PEO system. The microstructure revealed a severely disrupted interface characterized by vertical fissures, delaminated regions, and deep intrusions penetrating the Li substrate. These features are diagnostic of dendrite‐induced crack propagation, which arises from a localized current concentration and uneven Li^+^ flux across the interface. Sharp, channel‐like voids and fragmented SEI layers further indicate the mechanical collapse of the interphase during repeated cycling, accelerating Li filament propagation and amplifying interfacial stress. Together, these observations paint a clear picture of the electrochemical and mechanical instability, underscoring the inability of pristine PEO to regulate Li growth or maintain structural integrity under dynamic cycling conditions.

In stark contrast, the SEM image of the Li‐metal surface cycled with CSE‐30 (Figure 8b) reveals a uniform, compact, and dendrite‐free SEI morphology, indicative of a highly stable and well‐regulated interfacial environment. The surface remained topographically smooth, free of protrusions, voids, or filamentary deposits, with no evidence of “dead” Li accumulation. This structural uniformity underscores the synergistic role of the PDA@LLZO additive and PPP chains as mechanical fillers in stabilizing the Li‐electrolyte interface. The PDA@LLZO ceramic domains function as ion‐conductive scaffolds that homogenize the Li^+^ flux and minimize local overpotential gradients, while the flexible PPP matrix ensures intimate surface contact, dissipates mechanical stress, and prevents SEI delamination during cycling. Cross‐sectional SEM analysis further reinforced this stable interfacial architecture. The Li‐CSE‐30 interface was smooth, continuous, and crack‐free, with no visible voids or dendritic intrusions. The preserved flatness of the cross‐section demonstrates the formation of a mechanically robust and chemically passivating SEI capable of effectively blocking Li filament penetration. This superior stability arises from the cooperative action of the PDA@LLZO additives and the elastic PPP polymer network: the ceramic component acts as a physical barrier and flux homogenizer, whereas the polymeric scaffold accommodates strain and maintains cohesive interfacial adhesion. Such structural coherence directly correlates with the electrochemical performance, extended cycling stability, low interfacial resistance, and dendrite‐free Li deposition, as observed in both top‐view and cross‐sectional imaging. Collectively, these results demonstrate that CSE‐30 establishes a uniform, dendrite‐resistant, and mechanically resilient SEI, in sharp contrast to the fractured, dendrite‐infested interface formed with pristine PEO. The dense, continuous SEI maintained after prolonged cycling highlights the intrinsic interfacial stability and design efficacy of the CSE‐30 system, reinforcing its potential as a robust SSE platform for next‐generation LMBs [52].

The XPS spectra of the Li anode retrieved from the pristine PEO cell (Figure 8c) revealed a chemically complex and inhomogeneous SEI, consistent with the rough and dendrite‐laden surface observed in the SEM images. The C 1s spectrum shows strong contributions from the C─C/C═C, C─O─C, and O─C═O groups, indicative of extensive organic electrolyte decomposition and polymer degradation. Notably, the F 1s spectrum exhibits characteristic peaks corresponding to LiF and ─CF_2_/─CF_3_ moieties, indicating the partial decomposition of the LiTFSI salt. The formation of LiF, together with fluorinated organic residues, suggests the generation of an inorganic‐rich, yet structurally disordered, interphase during cycling [52]. The Li 1s spectrum is dominated by a strong Li_2_CO_3_/Li_2_O signal, whereas the LiF and Li_3_N components are present as significantly smaller and clearly resolved peaks, indicating that the SEI formed at the PEO/Li interface is primarily composed of inorganic oxides and carbonates, with LiF only as a minority phase and trace levels of Li_3_N. This composition reflects substantial electrolyte and anion decomposition but incomplete formation of the highly protective LiF‐rich layer, resulting in a heterogeneous organic–inorganic interphase. Additionally, the S 2p spectrum displayed multiple peaks for Li_2_S, Li_2_SO_4_, and RSO_3_^−^, indicating the extensive degradation of sulfonyl‐containing TFSI anions [53]. The N 1s signal was broad and convoluted, with overlapping peaks assigned to C─N, SO_2_─N, NO_3_^−^, and Li_3_N, again reflecting uncontrolled electrolyte decomposition and nitrogenous byproducts. These observations reveal a chemically unstable and mechanically fragile SEI, consistent with the rough, cracked surface and deep dendritic intrusions observed in the SEM images. The resulting interfacial heterogeneity facilitates dendrite propagation and accelerates the polarization buildup and premature shorting.

In stark contrast, the XPS spectra of the Li metal anode cycled with CSE‐30 (Figure 8d) exhibit a far more uniform and passivating interfacial chemistry, fully aligned with the smooth, crack‐free SEI observed in both surface and cross‐sectional SEM images (Figure 8b). The C 1s profile is dominated by peaks corresponding to O─C═O (Li_2_CO_3_, ROCO_2_Li) and minor C─O groups, indicative of controlled interfacial reactions and the effective formation of stable lithium alkyl carbonates. Importantly, the reduced intensity of the C─C/C═C and C─O─C signals suggest minimal polymer backbone degradation, demonstrating the chemical robustness of the engineered CSE‐30 matrix. The F 1s spectrum exhibits a sharp and dominant LiF peak, indicative of the formation of a highly uniform and ionically conductive LiF‐enriched SEI, which is a hallmark of stable interfaces in high‐performance Li‐metal systems. The Li 1s spectrum exhibits a pronounced shift toward a LiF‐rich interphase, with LiF evidently dominating the spectral response. The contributions from Li_2_CO_3_/Li_2_O are substantially reduced, whereas Li_3_N is more prominent compared to PEO. This suggests more complete anion decomposition and the formation of a stable inorganic SEI layer with minimal parasitic Li─O‐rich byproducts [10]. The S 2p peaks were limited to Li_2_S/Li_2_SO_3_, with the absence of higher‐order degradation products such as RSO_3_^−^ and SO_4_^2^^−^, confirming suppressed anion decomposition in CSE‐30. The O 1s spectrum supports the formation of a Li_2_O‐rich interphase with well‐defined lattice contributions. Meanwhile, the N 1s signal was sharp and singular, centered at Li_3_N, reinforcing the formation of beneficial inorganic SEI components that enhanced ionic conductivity and structural cohesion.

These chemical signatures collectively indicate that, in the CSE‐30 system, the controlled decomposition of LiTFSI and mild reduction of PEO/PPP chains at the Li surface yield a LiF, Li_3_N, Li_2_O, and Li_2_CO_3_ composite SEI (Figure 8e). The LiF and Li_3_N phases originate from the reductive cleavage of S─N and S─CF_3_ bonds in LiTFSI [54], forming an ionically conductive yet electronically insulating inner layer, while Li_2_CO_3_ and ROCO_2_Li arise from polymer reduction, providing chemical passivation and interfacial adhesion. Surface oxygen from LLZO contributes to Li_2_O formation, further reinforcing the interfacial stability [55]. The catechol and amine groups of PDA act as chemical scavengers that moderate radical intermediates and suppress the over‐decomposition of TFSI^−^, steering SEI evolution toward stable inorganic products rather than soluble organo‐sulfonates [56].

These XPS results highlight the mechanistic synergy within CSE‐30, where the PDA@LLZO fillers function as chemical stabilizers and Li^+^‐conductive sites that promote uniform SEI formation, while the PPP network imparts elasticity to prevent interfacial cracking. The resulting SEI was compositionally uniform, mechanically resilient, and electrochemically stable, maintaining structural integrity and suppressing dendrite growth during extended cycling.

**FIGURE 9:**
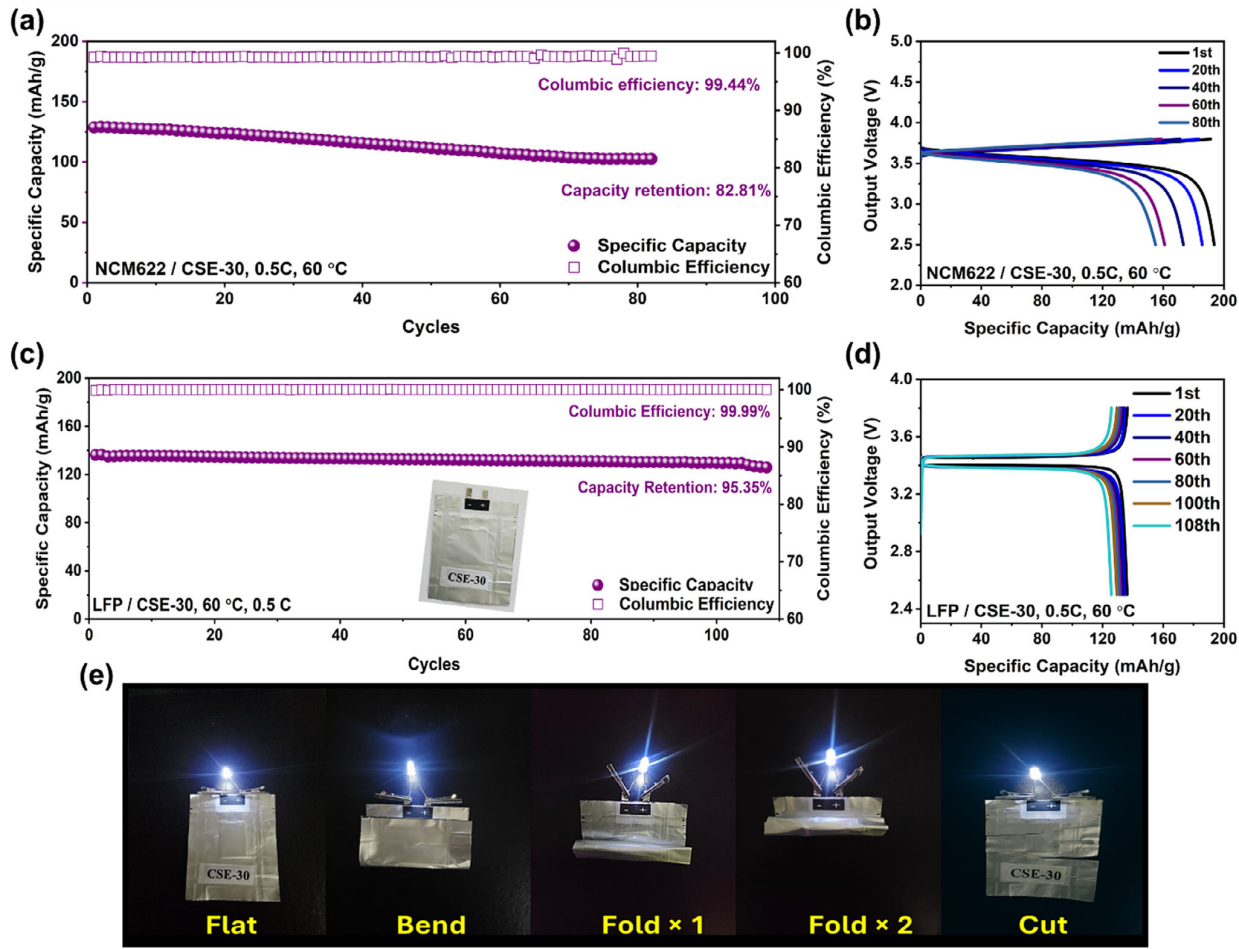
Electrochemical performance and mechanical robustness of CSE‐30 in full‐cell and flexible pouch cell configurations. (a) NCM622‖CSE‐30‖Li full cell at 0.5 C and 60°C delivers 128.74 mAh g^−^^1^ initially with >99% Coulombic efficiency and 80% capacity retention over 82 cycles. (b) Voltage profiles exhibit stable plateaus and minimal polarization drift, confirming interfacial compatibility with high‐voltage cathodes. (c) LFP‖CSE‐30‖Li pouch cell shows 92.45% capacity retention after 108 cycles with near‐unity Coulombic efficiency. (d) Overlapping charge–discharge curves indicate stable Li^+^ transport and suppressed interfacial degradation. (e) LED illumination under flat, bent, folded, and cut states demonstrates the mechanical durability and operational integrity of the CSE‐30 electrolyte.

To evaluate the practical applicability of the CSE‐30 system beyond conventional LFP cathodes, a full‐cell configuration was assembled using a high‐voltage NCM622 cathode and cycled at 0.5C and 60°C. The full cell exhibited an initial discharge capacity of 128.74 mAh.g^−^^1^ with a CE of 99.22%, reflecting stable interfacial kinetics and efficient Li^+^ utilization even under the elevated operating voltage of NCM‐based chemistry (Figure 9a). Over 82 cycles, the discharge capacity gradually declined to 102.57 mAh g^−^^1^, corresponding to a capacity retention of 82.81%. Remarkably, the CE remained consistently above 99% throughout the cycling period, signifying minimal side reactions and a stable SEI under the harsher electrochemical environment of layered oxide cathodes.

The voltage profiles (Figure 9b) across multiple cycles preserved a characteristic sloping plateau near 3.6–3.7 V, indicative of layered‐to‐spinel phase transitions in NCM622, with minimal polarization drift observed up to the 80^th^ cycle. This highlights the capability of the engineered CSE‐30 to accommodate the high‐voltage operation of Ni‐rich cathodes without compromising interfacial stability.

For comparison, a control full cell based on a conventional PEO electrolyte (NCM622‖PEO‖Li) was studied under identical conditions (Figures S36 and S37). The control cell revealed significantly inferior cycling stability, with a rapid capacity decay to 52.56% retention after only 29 cycles, despite maintaining a relatively high Coulombic efficiency (99.19%). This pronounced capacity fading reflects severe interfacial instability and accelerated degradation under high‐voltage application, exposing the limitations of PEO‐based systems in sustaining long‐term electrochemical performance with Ni‐rich cathodes.

The robust cycling behavior and sustained electrochemical reversibility affirm that the PDA@LLZO‐PPP reinforced CSE‐30 matrix can establish a conformal, ionically conductive, and chemically stable interphase, even under demanding cathode chemistries. These results underscore the versatility of the CSE‐30 electrolyte architecture, suggesting its compatibility with a broad range of cathode materials, including both olivine and layered oxide systems, thereby expanding its practical potential for integration into next‐generation LMBs.

To further assess the practical industrial applicability and mechanical resilience of the engineered CSE‐30 electrolyte, a flexible pouch cell (LFP‖CSE‐30‖Li) was assembled and evaluated at 60°C at a moderate rate of 0.5 C. For comparative analysis, a control pouch cell based on a conventional PEO electrolyte (LFP‖PEO‖Li) was also assembled and analyzed under identical conditions. The long‐term galvanostatic cycling performance, shown in Figure 9c, exhibited a high initial discharge capacity of 136.15 mAh g^−^^1^, with a remarkable capacity retention of 125.83 mAh g^−^^1^ after 108 cycles, corresponding to a retention rate of 92.45%. Notably, the CE remained nearly ideal, increasing slightly from 99.83% in the first cycle to 99.99% in the final cycle, suggesting highly reversible charge/discharge kinetics and minimal parasitic side reactions over prolonged operation. In contrast, the PEO‐based control pouch cell revealed rapid capacity fading, retaining only 72.24% of its initial capacity after 50 cycles, accompanied by a lower Coulombic efficiency of 89.60% and marked instability during prolonged cycling (Figure S38).

The corresponding charge–discharge voltage profiles (Figure 9d) displayed minimal polarization drift over 108 cycles, with overlapping plateaus and sharp redox features. These results strongly indicate a highly stable Li^+^ transport mechanism and interfacial integrity enabled by the CSE‐30 structure. In contrast, the PEO‐based pouch cell showed significant polarization growth, progressive distortion of voltage profiles, and loss of redox definition with cycling, ultimately leading to cell failure by the 50^th^ cycle (Figure S39). These results explicitly reveal that the CSE‐30 electrolyte stabilizes the electrode–electrolyte interface, suppresses polarization buildup, and enables sustained and highly reversible Li^+^ transport under practical pouch‐cell applications. This electrochemical consistency is consistent with earlier symmetric cell observations and underscores the critical role of the trilayered CSE architecture in improving ionic conductivity, suppressing dendrite growth, and overcoming the intrinsic limitations of conventional PEO‐based systems.

Beyond electrochemical cycling, the physical integrity and resilience of the CSE‐30‐based pouch cell were validated under harsh mechanical deformation tests. As shown in the photographic demonstration (Figure 9e), the pouch cell maintained uninterrupted LED illumination under three different conditions: flat, folded, and partially cut, highlighting its mechanical durability and functional reliability under real‐world stress scenarios. The ability to withstand such aggressive mechanical perturbations without failure is indicative of a mechanically robust electrolyte membrane and strong interfacial adhesion, which are essential for flexible or wearable solid‐state battery applications [57]. Collectively, these results emphasize the industrial viability of the CSE‐30 system, demonstrating not only excellent electrochemical performance in multilayer pouch configurations but also resilience to mechanical abuse. These characteristics are pivotal for the translation of laboratory‐scale innovations into commercial‐grade, safe, and high‐performance solid‐state LMBs.

## 4 Conclusion

This study presents a rationally engineered trilayer composite solid electrolyte (CSE) that integrates interfacial chemistry, mechanical design, and architectural engineering to overcome the long‐standing challenges of PEO‐based SSEs for LMBs. Through the synergistic incorporation of a polydopamine‐functionalized LLZO additive and a ductile triblock copolymer (PPP) within a spatially resolved multilayer framework, the CSE‐30 formulation achieves a unique balance between ionic conductivity, mechanical robustness, and interfacial stability. The PDA modification not only preserves the structural integrity of the LLZO garnet phase but also introduces a chemically adaptive interface that enhances polymer–ceramic compatibility, mitigates interfacial resistance, and promotes a homogeneous Li^+^ flux. Simultaneously, the PPP network acts as a flexible, energy‐dissipating matrix that bridges the rigid filler domains and distributes the mechanical strain, thereby suppressing the SEI rupture and dendritic intrusion during cycling. The optimized CSE‐30 achieved an impressive ionic conductivity of 5.60 × 10^−^^3^ S.cm^−^^1^ at 60°C and a high lithium transference number of 0.81, accompanied by exceptional mechanical strength and elongation. Symmetric Li|Li cells exhibited dendrite‐free cycling for over 1000 h, whereas full LiFePO_4_|Li cells delivered a stable discharge capacity of 133.6 mAh g^−^^1^ at 0.5C with 80% retention after 1000 cycles, underscoring both electrochemical durability and interfacial resilience. Morphological and spectroscopic examinations further revealed that CSE‐30 fosters the formation of a conformal, chemically uniform SEI enriched with LiF and polymer‐derived organic species, which collectively ensure interfacial continuity and electrochemical stability. These results establish a robust structure–property correlation, wherein the synergistic interaction between a surface‐engineered ceramic additive and a mechanically compliant polymer matrix yields a multifunctional electrolyte capable of sustaining high‐performance LMBs. The integrated design approach couples chemical functionalization with architectural layering to rationally tailor the transport and mechanical integrity, which not only advances the practical viability of polymer‐based LMBs but also offers a broadly applicable framework for interfacial engineering in next‐generation energy storage systems.

## Conflicts of Interest

The authors declare no conflicts of interest.

## Supporting information




**Supporting File**: adma73879‐sup‐0001‐SuppMat.docx.

## Data Availability

The data that support the findings of this study are available from the corresponding author upon reasonable request.
