# Supplementary material for: Surface‐Functionalized LLZO‐Incorporated Multilayer Composite Solid Electrolytes for Dendrite Suppression and Efficient Ionic Conduction in Lithium–Metal Batteries
Source: Adv Mater. 2026 Jun 25;38(43):e73879. doi: 10.1002/adma.73879 (PMC13431703; doi:10.1002/adma.73879)
Supplement: Supplementary file 1 — Supporting File: adma73879‐sup‐0001‐SuppMat.docx. [file ADMA-38-e73879-s001.docx]

**Supporting Information**

**Surface-functionalized LLZO-incorporated Multilayer Composite Solid Electrolytes for Dendrite Suppression and Efficient Ionic Conduction in Lithium-Metal Batteries**

Fazal Ur Rehman^1^, Minhong Woo^1^, Hyesoo Choi^1^, Jihwan Kim^1^, Yujin Kim^1^, Sanghee Park^1^, Serim Ahn^1^, Jinsub Lim^2^, Minyoung Kim^2^, Mincheol Chang*^1,3^

^1^Department of Polymer Engineering, Chonnam National University, Gwangju-61186, South Korea

^2^Korea Institute of Industrial Technology, Cheomdan-gwagiro 208-gil, Buk-gu, Gwangju, 61012, South Korea

^3^School of Polymer Science and Engineering, Chonnam National University, Gwangju-61186, South Korea

**Corresponding Author**

*E-mail: [mchang35@chonnam.ac.kr](mailto:mchang35@chonnam.ac.kr)

**Materials**

Poly(ethylene oxide) (PEO, Mw = 6 × 10⁵ g mol⁻¹) and poly(propylene glycol)-block-poly(ethylene glycol)-block-poly(propylene glycol) (PPG-PEG-PPG) were purchased from Sigma-Aldrich and used without further purification. Lithium bis(trifluoromethanesulfonyl)imide (LiTFSI, battery grade) and N-methyl-2-pyrrolidone (NMP) were obtained from MTI Corporation. Acetonitrile (anhydrous, 99.8%) and ethanol (anhydrous, 99.9%) were sourced from Sigma-Aldrich and DAEJUN Chemicals & Metals Co., Ltd., respectively. LiFePO₄ (LFP) powder, Super P conductive carbon, and poly(vinylidene fluoride) (PVDF, KF1300) were procured from MTI Corporation and Kureha Corporation. Lithium metal foil (diameter = 16 mm, thickness = 0.75 mm) was purchased from MTI Corporation. Tetragonal garnet-type Li₇La₃Zr₂O₁₂ (LLZO) ceramic powder was supplied by the Korea Institute of Industrial Technology (KITECH, South Korea). LiNi₀.₆Co₀.₂Mn₀.₂O₂ (NCM622) was purchased from MTI Corporation. All materials were stored and handled in an argon-filled glovebox (H₂O, O₂ < 0.1 ppm) unless otherwise specified.

**In-situ polymerization of poly(dopamine) for surface modification of LLZO**

Polydopamine-coated LLZO (PDA@LLZO) composites were prepared via an *in situ* polymerization approach in an ethanolic medium. Initially, 19 mg of dopamine hydrochloride was dissolved in 10 mL of ethanol under vigorous magnetic stirring. To initiate polymerization, 6 mg of Tris-HCl buffer (pH adjustment grade) was added to achieve a pH range of 8.5–10.0, thus providing an appropriate basic environment for dopamine polymerization. The mixture was stirred at 400 rpm and room temperature for 24 h to allow oxidative self-polymerization of dopamine, forming a poly(dopamine) (PDA) network. Subsequently, 0.5 g of LLZO powder was added directly to the polymerized solution and stirred under identical conditions for an additional 24 h to ensure uniform coating of PDA onto the ceramic surface. The obtained dispersion was filtered and washed thoroughly with ethanol to remove the residual reactants. The recovered powder was dried in a vacuum oven at 80 °C for 12 h and subsequently stored in an argon-filled glovebox. Unlike previous reports that employed methanol-based systems, ethanol was deliberately chosen as the solvent because of its superior coating performance in this study. The intermediate polarity of ethanol and its weaker hydrogen bonding compared to those of methanol were found to reduce uncontrolled PDA aggregation and facilitate the formation of a more conformal, defect-free coating on the LLZO surface. This tailored surface chemistry is essential for enhancing the polymer–ceramic interfacial compatibility in the subsequent composite electrolyte system.

**Materials Characterization**

Fourier-transform infrared spectroscopy (FTIR, PerkinElmer) was employed to identify the chemical bonding and functional groups present in the materials within the wavenumber range of 4000–400 cm⁻¹. XRD patterns of LLZO and PDA-coated LLZO were recorded using an Empyrean diffractometer (Malvern Panalytical) equipped with a Cu Kα X-ray source (40 kV, 50 mA), operating at a scan speed of 4°/min to 10°/min over an angular range of 10°–80° (2θ). The thermal properties, including phase transitions and thermal stability, were evaluated through thermogravimetric analysis (TGA) using a TGA analyzer (TA, Q-50) over a temperature range of 25^o^C to 800 ^o^C with 10^o^C/min and differential scanning calorimetry (DSC) measurements were conducted on a DSC3 instrument (Mettler Toledo) over a temperature range of −80°C to 90°C at a heating rate of 5°C/min. The surface morphology and structural details of the samples were analyzed using a field emission scanning electron microscope (FE-SEM, JSM-7900F) to observe the surface features and a transmission electron microscope (TEM, JEM-2010) to investigate the PDA coating on the LLZO surface and its finer structural details at the nanoscale. Elemental mapping was performed using energy-dispersive X-ray spectroscopy (EDS) to determine the distribution of the key elements in the samples. The chemical composition and oxidation states of the elements were determined using X-ray photoelectron spectroscopy (XPS) with a K-Alpha+ spectrometer (Thermo Fisher Scientific) equipped with an Al Kα X-ray source. Metallic elemental analysis was conducted using Inductively Coupled Plasma Optical Emission Spectroscopy (ICP-OES) (iCAP 7400 Duo MFC, Thermo Fisher Scientific).

**Electrolyte Fabrication**

The tri-layered sandwich CSE was prepared via a sequential solution casting and thermal lamination process conducted under an inert atmosphere. The outer electrolyte layers were composed of PEO and LiTFSI mixed at an ethylene oxide to Li^+^ ion molar ratio of 15:1. Both components were dissolved in anhydrous ACN under magnetic stirring to form a homogeneous solution, which was cast onto Teflon substrates and dried in an Ar-filled glove box at room temperature overnight to remove the residual solvent.

For the central layer, PDA-coated LLZO composites were incorporated into the same PEO/LiTFSI matrix at varying concentrations of 10, 20, 30, and 40 wt% relative to PEO. Additionally, 2 wt% poly(propylene glycol)-block-poly(ethylene glycol)-block-poly(propylene glycol) (PPP) was added as a mechanical reinforcing agent. The mixture was dispersed under stirring and sonication to ensure uniform slurry formation, cast onto a Teflon substrate under an argon atmosphere, and dried in an Ar-filled glove box at room temperature overnight to remove the residual solvent.

After drying, the two outer PEO/LiTFSI films and the central PDA-coated LLZO/PPP-containing film were sequentially stacked and laminated via thermal compression using a hot press at 60 °C under a mechanical pressure of 5 MPa for 10 min to ensure strong interfacial contact and structural cohesion. The resulting membrane was mechanically flexible and exhibited continuous ionic pathways across all layers. Its gradient-phase structure was engineered to enhance mechanical durability and suppress Li dendrite propagation during extended electrochemical cycling.

**LFP & NCM622 Cathode Fabrication**

LiFePO₄ (LFP) cathodes were fabricated using a conventional slurry casting method with a weight ratio of LFP:Super-P:poly(vinylidene fluoride) (PVDF) = 7.5:1.5:1. Super-P conductive carbon was pre-dried at 110 °C in a vacuum oven overnight to eliminate any residual moisture. A 5wt% PVDF binder solution was prepared by dissolving 0.05 g of PVDF in 0.95 g of N-methyl-2-pyrrolidone (NMP) under vigorous magnetic stirring until a uniform solution was achieved.

Separately, 0.3 g of LFP powder and 0.06 g of dried Super-P were weighed and loaded into a ball mill vial inside an argon-filled glove box to prevent atmospheric contamination. The mixture was subjected to ball milling to ensure a homogenous dispersion of the active material and conductive additive. The resulting composite was then combined with a PVDF binder solution to form a uniform slurry, which was cast onto aluminum foil (battery grade) using a doctor blade. The electrode films were dried in a vacuum oven at 110 °C for 24 h to remove residual solvent and achieve uniform adhesion. For the NCM-based cathodes, the same procedure, composition ratios, and processing conditions were employed, with LiNi₀.₆Co₀.₂Mn₀.₂O₂ (NCM622, MTI Corporation) was used instead of LFP as the active material at an identical weight of 0.3 g. The dried cathodes were stored in an argon-filled glove box until cell assembly.

**Assembly of Solid-State Li-Metal Batteries**

CR2032-type coin cells were assembled to evaluate the electrochemical performance of the trilayered composite solid electrolyte (CSE) in lithium-metal battery configurations. The cell architecture consisted of an LFP cathode (14 mm diameter, areal loading ≈ 2.3 mg cm⁻²), lithium metal anode (16 mm diameter, 200 μm thickness; MTI Corporation), and CSE membrane (19 mm diameter) positioned between the electrodes. All assembly steps were conducted in an argon-filled glove box (H₂O, O₂ < 0.1 ppm) to prevent contamination by ambient moisture or oxygen. No liquid electrolyte or separator was used in this study. The assembled coin cells were rested in a glovebox for 24 h to allow for interfacial stabilization (aging) prior to electrochemical testing to establish robust all-solid-state battery configurations.

**Electrochemical Studies**

The ionic conductivity (σ) of all tri-layered CSEs was determined via electrochemical impedance spectroscopy (EIS) using symmetric stainless steel (SS|CSE|SS) blocking cells. Measurements were performed over a temperature range of 25–60 °C using a WizEIS-1200 Premium impedance analyzer (NanoTech, Korea) with an AC amplitude of 10 mV in the frequency range of 1 MHz to 1 Hz. The bulk resistance (R_b_) was extracted from the high-frequency intercept of the Nyquist plot, and the ionic conductivity (σ) was calculated using the relation:

$$\sigma= \frac{T}{S\times R_{b}}$$

Where T is the thickness of the CSE; S is the area of the SS electrode; and R_b_ is the bulk resistance of CSE.

Linear sweep voltammetry (LSV) was conducted using Li|CSE|SS cells to evaluate the electrochemical stability window of the CSEs. Stainless steel served as the working electrode, and Li-metal acted as both counter and reference electrodes. The scans were recorded from 2.5 V to 5.5 V at a scan rate of 1 mV s⁻¹.

The lithium-ion transference number (t⁺) was determined via the *Vincent–Bruce method* using Li|CSE|Li symmetric cells. A DC polarization voltage of 10 mV was applied, and EIS spectra were collected before and after polarization with a 10 mV amplitude over the frequency range of 1 MHz to 1 Hz. The transference number (t⁺) was calculated using the equation:

$$t^{+}= \frac{I_{ss}(\Delta V-I_{0}R_{0}}{I_{0}(\Delta V-I_{s}R_{ss})}$$

Where ΔV is the applied polarization−voltage; I_0_ and I_SS_ are the initial and steady-state current, respectively; and R_0_ and R_SS_ are the initial and steady-state interfacial resistance after polarization for 2 h, respectively.

**Mechanical Strength Evaluation**

To rigorously assess the mechanical robustness of solid-state polymer electrolytes (SPEs) and their composite counterparts (CSEs), uniaxial tensile testing was performed using a universal testing machine (UTM) operated at room temperature. Electrolyte films were cast into freestanding membranes and precisely cut into rectangular strips with a fixed gauge length of 20 mm. Each specimen was mounted vertically, and tensile force was applied under a constant strain rate until mechanical failure. The force–displacement data were recorded in real time and subsequently converted into engineering stress–strain plots for quantitative evaluation of elasticity, tensile strength, elongation, and modulus.

The engineering stress (σ, MPa) was computed using the classical relation:

$$\text{Stress (MPa)}=\frac{\text{Force (N)}}{\text{Width (m)}\times\text{Thickness (m)}}\times{10}^{-6}$$

This equation normalizes the applied force by the cross-sectional area (in m²), with a scaling factor to convert from Pa to MPa. The strain (ε, %) was calculated as the ratio of extension to original gauge length, multiplied by 100, yielding relative deformation in percentage terms.

**Statistical Analysis**

All mechanical tensile tests were performed on three independent samples (n = 3) for each electrolyte composition using a universal testing machine (UTM) under ambient conditions. The reported tensile strength and elongation at break values represent the mean of these measurements, and data are presented as mean ± standard deviation (SD). Linear regression analysis was applied to the elastic region of the stress–strain curves to extract the slope, R², and Pearson's r values for each composition, from which the modulus was assessed. All data processing, linear fitting, and statistical calculations were performed using OriginPro software.

**Computational Calculations**

All computational calculations were performed using the Gaussian 09W program package. The molecular systems comprising ethylene oxide, LiTFSI, dopamine, PPP, poly(dopamine) oligomers, and the crystalline LLZO surface slab, were fully optimized prior to further analysis, and their representative geometries are shown in **Figure S1**. The geometry optimizations and subsequent property evaluations were conducted using density functional theory (DFT) at the B3LYP/6-31G(d) level of theory. The geometries of the individual molecules and their complexes were optimized using the density functional theory (DFT) method with the B3LYP exchange-correlation functional and the 6-31G(d) basis set. Frontier molecular orbital energies (HOMO and LUMO) were calculated to evaluate the electronic stability and reactivity of individual components and complexes. Intermolecular interactions were investigated by computing binding energies (ΔE_b_) for selected ion–molecule systems involving Li⁺, TFSI⁻, ethylene oxide, dopamine, and PPP segments. Binding energies were obtained using the supermolecular approach according to the following expression:

Δ*E_b_* = *E_total_* − ∑*E_frag_* ​

Where *E*_total_ ​ represents the overall electronic energy of the complexes, *E*_frag_ ​denotes the electronic energies of individual fragments. To ensure that the calculated binding energies were not influenced by arbitrary initial configurations, multiple feasible starting geometries were constructed for representative ion–molecule systems by changing their relative orientation and approach of Li⁺ or TFSI⁻ toward the coordination-active sites. All initial structures were subsequently subjected to full unconstrained geometry optimization, allowing the complexes to relax to their respective lowest-energy configurations on the potential energy surface. The final binding energies were evaluated exclusively from these optimized equilibrium structures. This approach minimizes any bias associated with the initial positioning and ensures that the reported energetics reflect the precise interaction characteristics of the molecular systems.

Electrostatic potential (ESP) and electron localization function (ELF) analyses were performed to investigate the electronic structure and Li⁺ coordination tendencies of electrolyte components. ESP maps were generated using the Jmol visualization package to identify regions of electrostatic nucleophilicity and electrophilicity, thereby elucidating potential Li⁺ binding sites and anion-repelling domains. ELF calculations were carried out using the Multiwfn 3.8 software package to assess localized electron density distributions and bonding topologies across key functional moieties.

All ESP and ELF computations were based on geometries optimized at the B3LYP/6-31G(d) level of theory using Gaussian 09W. These complementary descriptors provided spatially resolved insights into cation–polymer interactions and supported mechanistic interpretation of Li⁺ transport and anion exclusion behavior within the composite solid electrolyte matrix.

**Figure S1: Molecular and crystal models of electrolyte constituents used in DFT calculations.** The optimized structures of ethylene oxide, LiTFSI, dopamine, PPP triblock copolymer, Tris-HCl, poly(dopamine) oligomer, and LLZO crystal slab are shown with atom-specific color coding. These representative models were employed for binding energy, electrostatic potential, and orbital energy calculations to elucidate the molecular-level interactions within the composite solid electrolyte system.


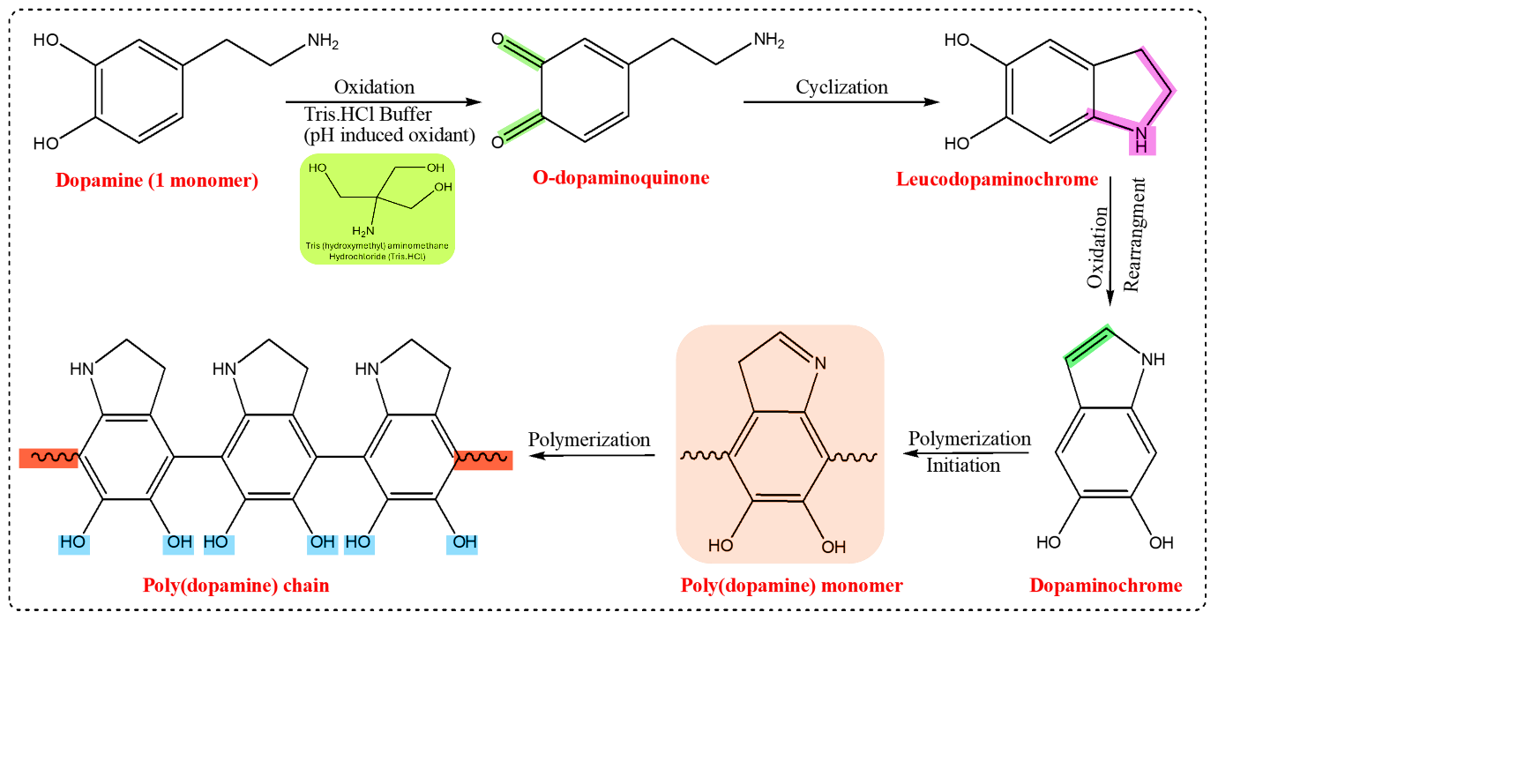


**Figure S2: Schematic illustration of the oxidative polymerization mechanism of dopamine to form poly(dopamine).** The process begins with the oxidation of dopamine monomers in a Tris-HCl buffer, which acts as a pH-induced oxidant. Dopamine is initially converted to its oxidized form, o-dopaminoquinone, via the removal of electrons from its catechol groups. This intermediate undergoes an intramolecular cyclization, forming leucodopaminochrome, a critical precursor for polymerization. Subsequent oxidation and rearrangement of leucodopaminochrome lead to the formation of dopaminochrome, a reactive compound containing an indole-like structure. Dopaminochrome undergoes polymerization initiation, driven by its high reactivity and ability to form covalent bonds, resulting in the formation of PDA monomers. These monomers further polymerize into long PDA chains, characterized by their conjugated structures and rich presence of hydroxyl (-OH) and amino (-NH) functional groups. The process ensures the uniform deposition of PDA as a surface coating, with the polymer's structure providing excellent adhesion, chemical stability, and functionalization capability. This mechanism is highly efficient for modifying various material surfaces, as it integrates the self-polymerizing nature of dopamine with the chemical versatility of its functional groups.


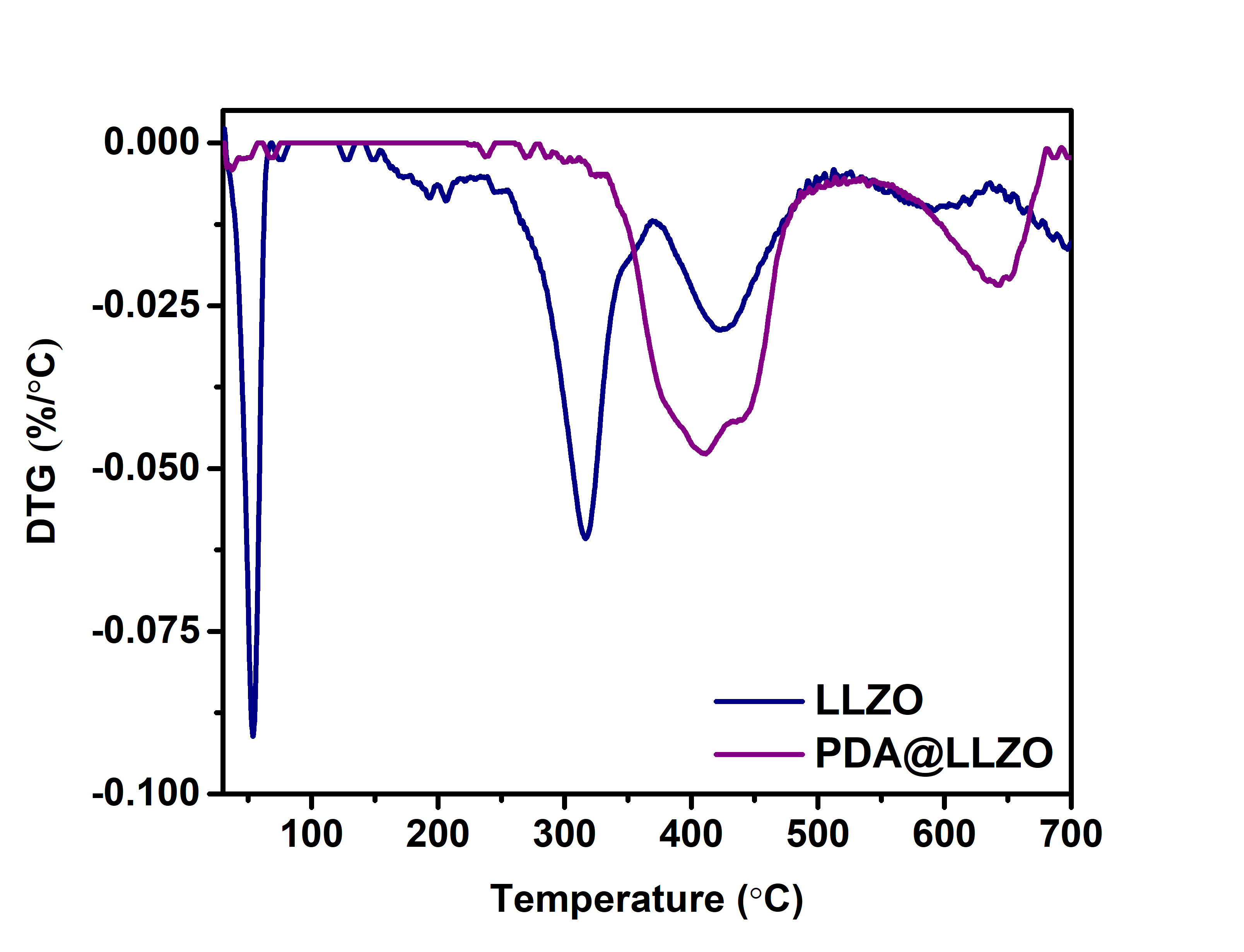


**Figure S3: Derivative thermogravimetric (DTG) profiles of pristine LLZO and PDA-functionalized LLZO (PDA@LLZO)**. It illustrates the rate of mass loss as a function of temperature. The emergence of distinct decomposition features in PDA@LLZO between 300–500 °C, absent in the uncoated sample, confirms the thermal degradation of the polydopamine layer and validates successful surface functionalization. The altered decomposition pathway further reflects the interfacial interaction and thermal modulation imparted by the organic coating.


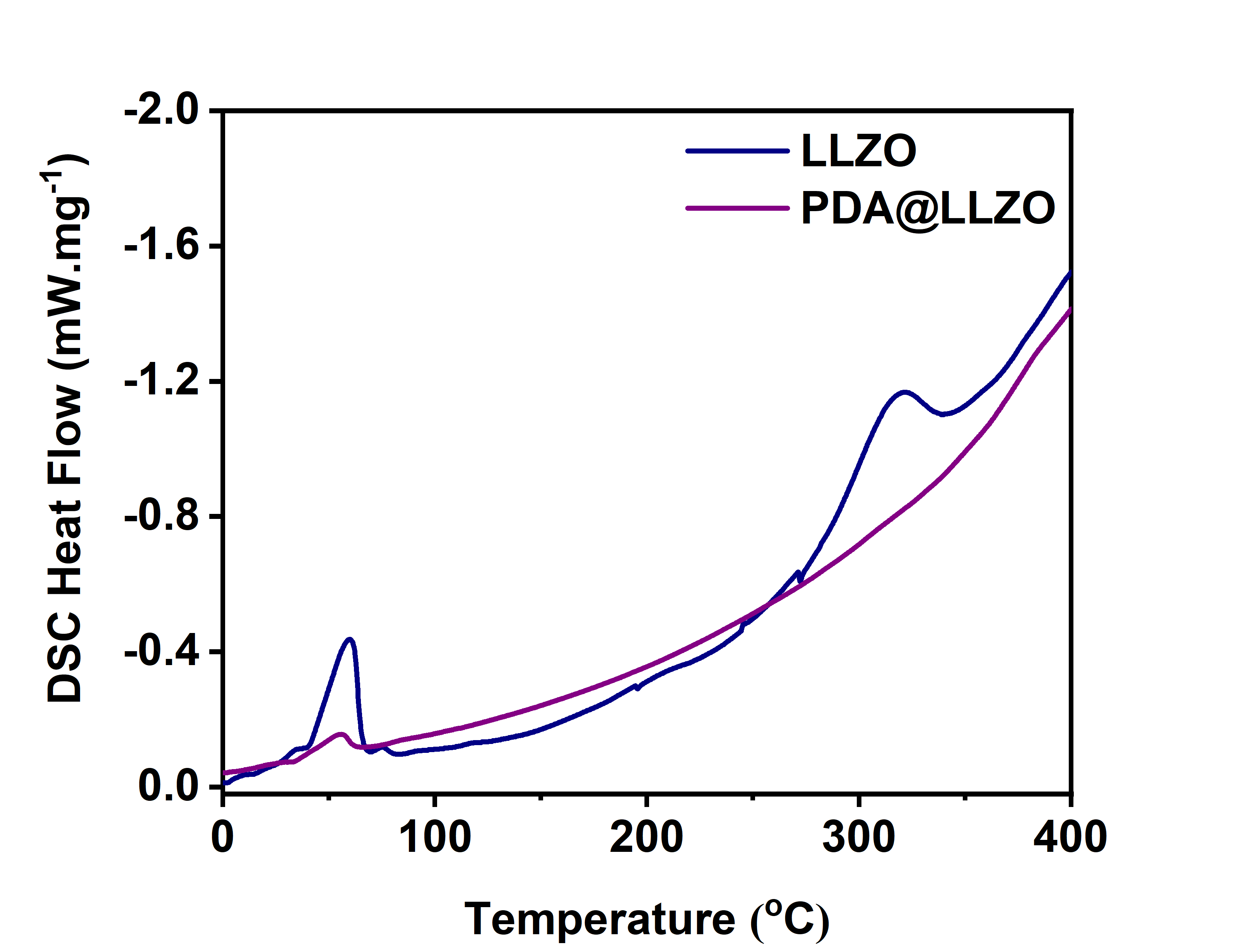


**Figure S4: DSC curves of LLZO and PDA@LLZO, revealing thermal transitions associated with PDA decomposition and structural stability, indicating the thermal behavior of the PDA@LLZO particles.**


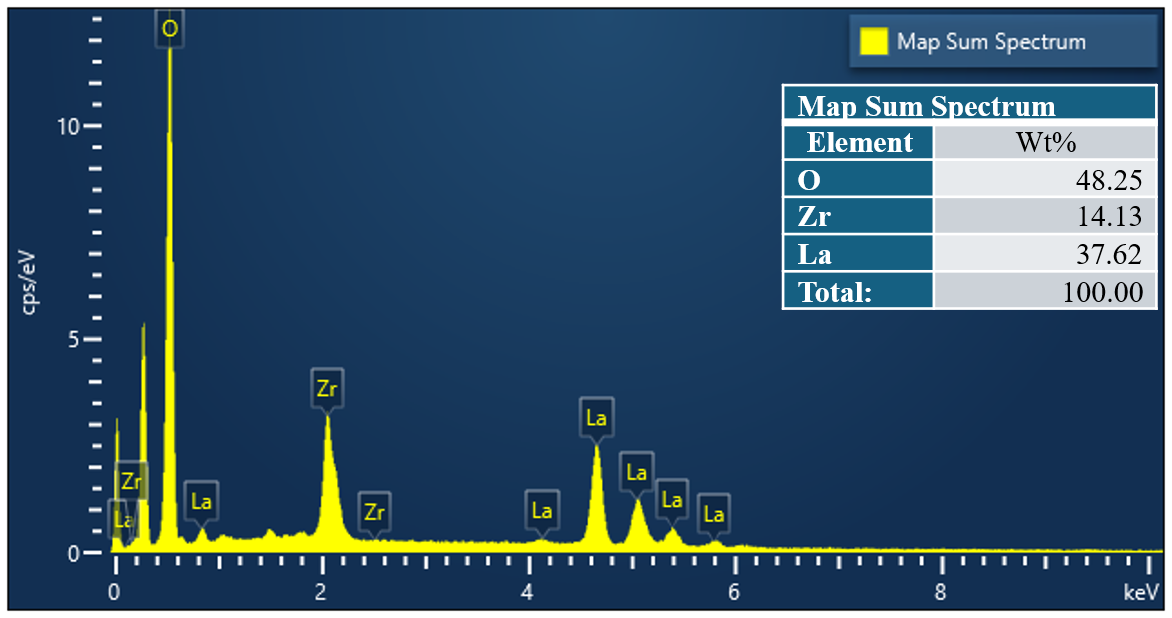


**Figure S5: EDS spectrum of pristine LLZO.** It confirmed the presence of O, Zr, and La with their respective weight percentages, confirming the characteristic garnet-phase composition. The absence of carbon (C) and nitrogen (N) signals confirms that the LLZO surface is unmodified, lacking any organic coating or functionalization. The high oxygen content (48.25 wt%) indicates a fully oxidized state, ensuring the structural stability of LLZO, making it suitable for use in solid-state electrolyte applications.


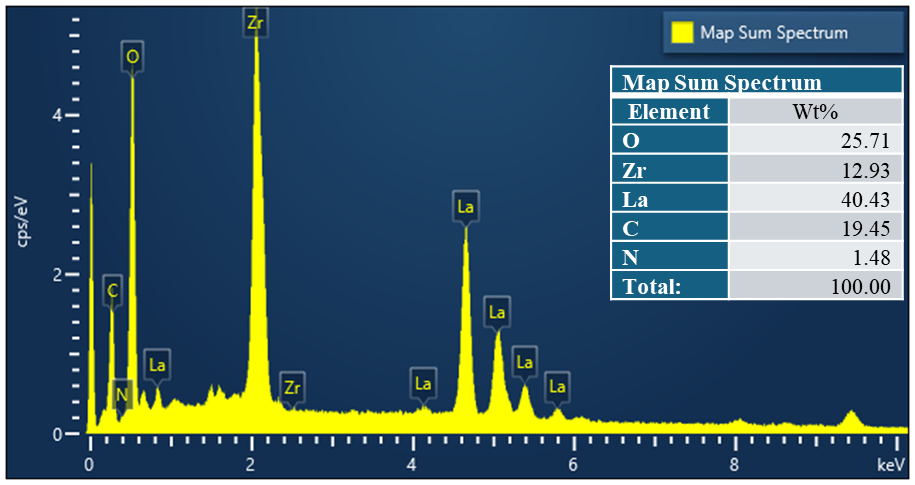


**Figure S6: EDS spectrum of PDA-coated LLZO.** It confirmed the presence of carbon (C) and nitrogen (N) along with O, Zr, and La, indicating successful polydopamine surface modification. The appearance of C (19.45 wt%) and N (1.48 wt%) signals confirms the deposition of the polydopamine (PDA) layer, validating the surface modification of LLZO. The reduced oxygen content (25.71 wt%) compared to pristine LLZO suggests partial surface coverage by PDA, which enhances interfacial compatibility in polymer electrolytes.


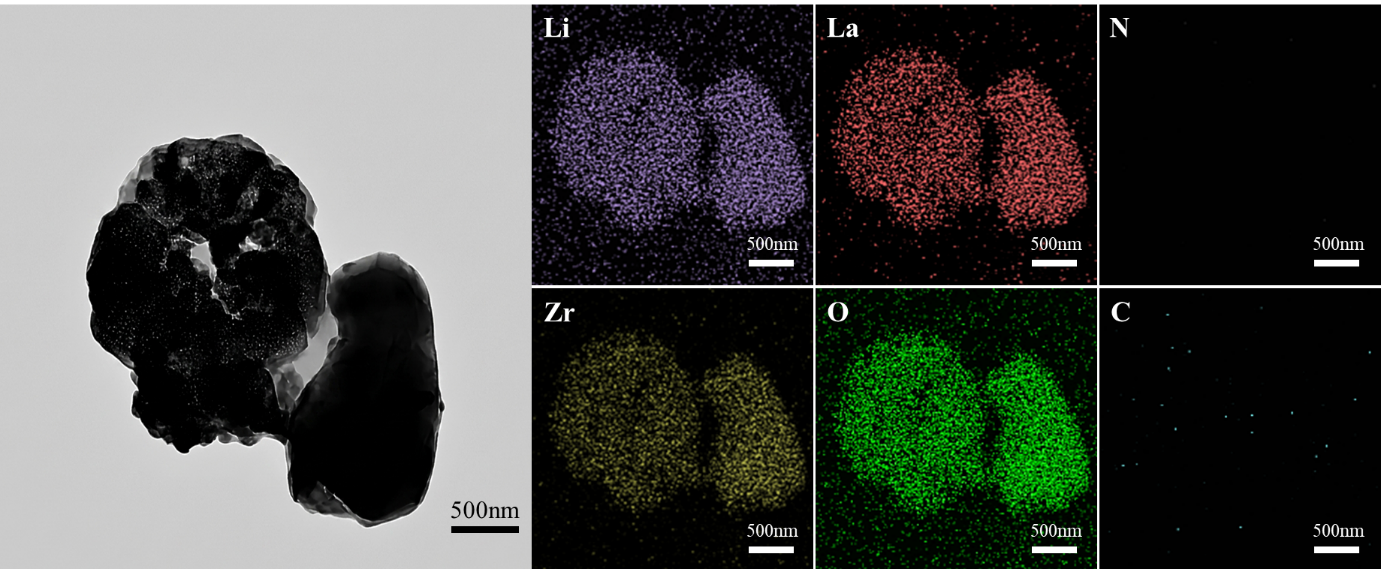


**Figure S7: TEM image and corresponding EDS elemental mapping of the pristine LLZO prior to the PDA coating.** The maps confirm the uniform distribution of La, Zr, and O throughout the particle, consistent with the intrinsic garnet structure of LLZO. No detectable nitrogen signal is observed, and only negligible carbon is present, which is attributed to residual ethanol used during sample preparation rather than the material itself. The absence of N and minimal C signals verifies the chemically clean surface of pristine LLZO before surface modification.


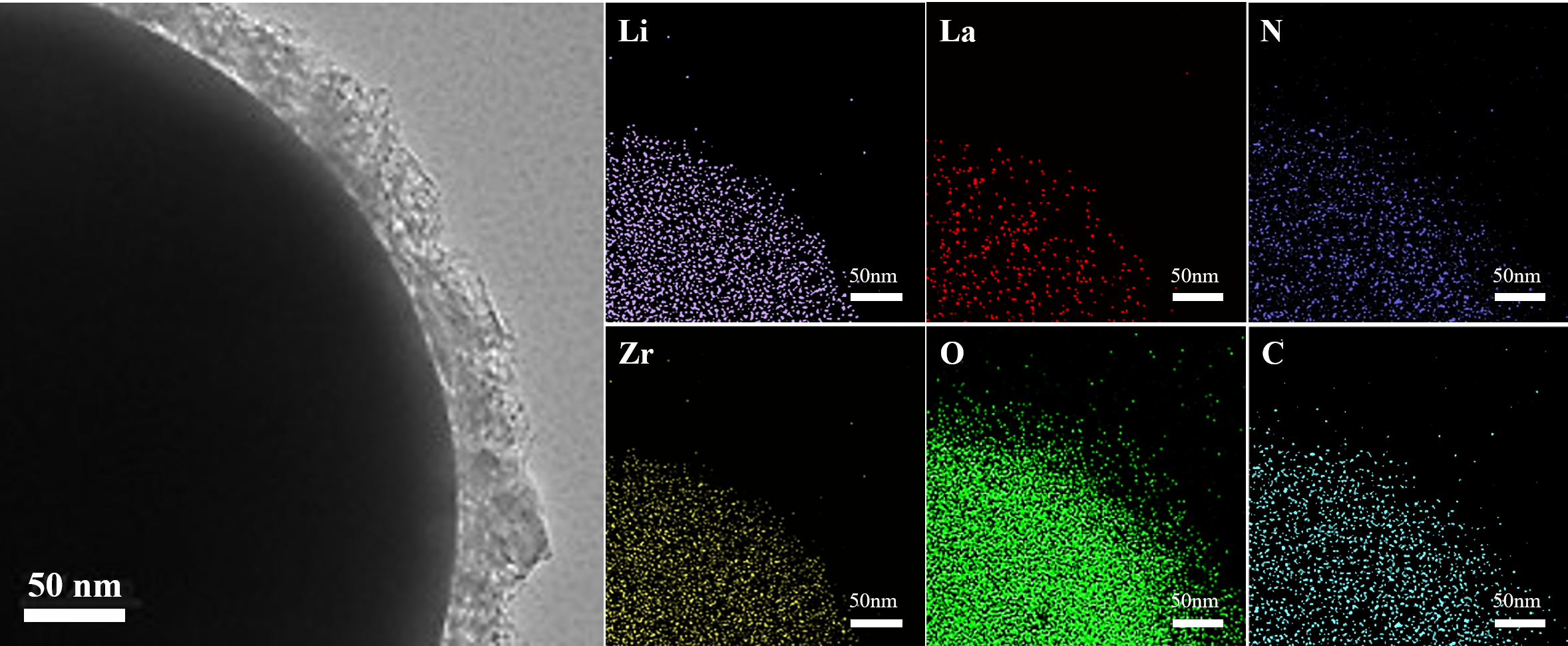


**Figure S8: TEM image and corresponding EDS elemental mapping of PDA-coated LLZO (PDA@LLZO).** In addition to the intrinsic elements (La, Zr, and O), clear signals of carbon (C) and nitrogen (N) are observed and uniformly distributed over the particle surface, confirming the successful deposition of the polydopamine layer. The spatial distribution shows that C and N are predominantly localized in the outer shell region, whereas the intensities of La and Zr decrease toward the particle edge, indicating the formation of a conformal core–shell structure. This uniform elemental distribution validates the homogeneous nature of the PDA coating and its effective surface coverage on the LLZO.

**Table S1: Quantitative analysis of elemental composition and stoichiometric reconstruction of pristine LLZO and PDA@LLZO based on Inductively Coupled Plasma Optical Emission Spectroscopy (ICP-OES).**

| Sample | Element | Conc. (mg/L) | Mass  (mg) | Mass  (g) | Moles  (mol) | Ratio  (÷ Zr) | Final Ratio  (Zr = 2) |
| --- | --- | --- | --- | --- | --- | --- | --- |
| LLZO | Li | 5.80 | 0.580 | 5.80×10⁻⁴ | 8.36×10⁻⁵ | 3.53 | 7.01 |
|  | La | 49.46 | 4.946 | 4.946×10⁻³ | 3.56×10⁻⁵ | 1.50 | 2.99 |
|  | Zr | 21.62 | 2.162 | 2.162×10⁻³ | 2.37×10⁻⁵ | 1.00 | 1.99 |
| PDA@LLZO | Li | 5.77 | 0.577 | 5.77×10⁻⁴ | 8.31×10⁻⁵ | 3.52 | 6.98 |
|  | La | 49.14 | 4.914 | 4.914×10⁻³ | 3.54×10⁻⁵ | 1.50 | 2.97 |
|  | Zr | 21.51 | 2.151 | 2.151×10⁻³ | 2.36×10⁻⁵ | 1.00 | 1.98 |

***Mathematical expressions used:**

- Mass (mg) = Concentration × 0.1 L
- Moles = Mass (g) / Atomic weight
- Ratio = Moles / Moles(Zr)
- Final Ratio = Ratio × 2

**Table S2: Energy Levels and Energy Band Gaps (E_g_) calculated after DFT calculations**

| Material | HOMO | LUMO | E_g_ (eV) |
| --- | --- | --- | --- |
| PEO | -8.64 | -0.96 | 7.68 |
| LiTFSI | -8.13 | -1.48 | 6.65 |
| Dopamine | -5.61 | -1.09 | 4.52 |
| PPP | -6.39 | -1.79 | 4.60 |


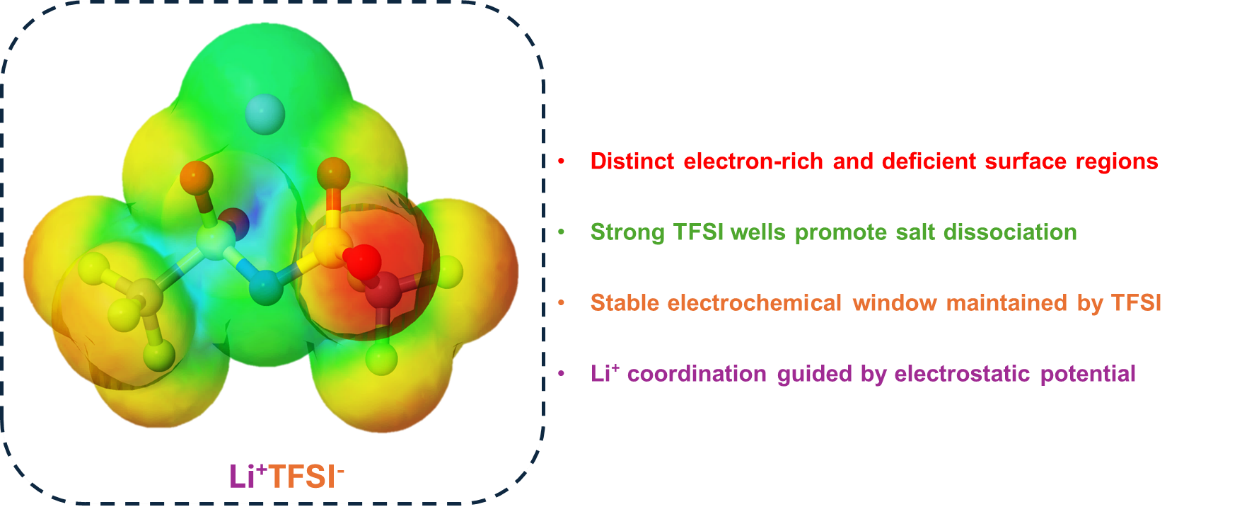


**Figure S9: ESP of LiTFSI.** It shows widespread electronegative regions promoting salt dissociation.


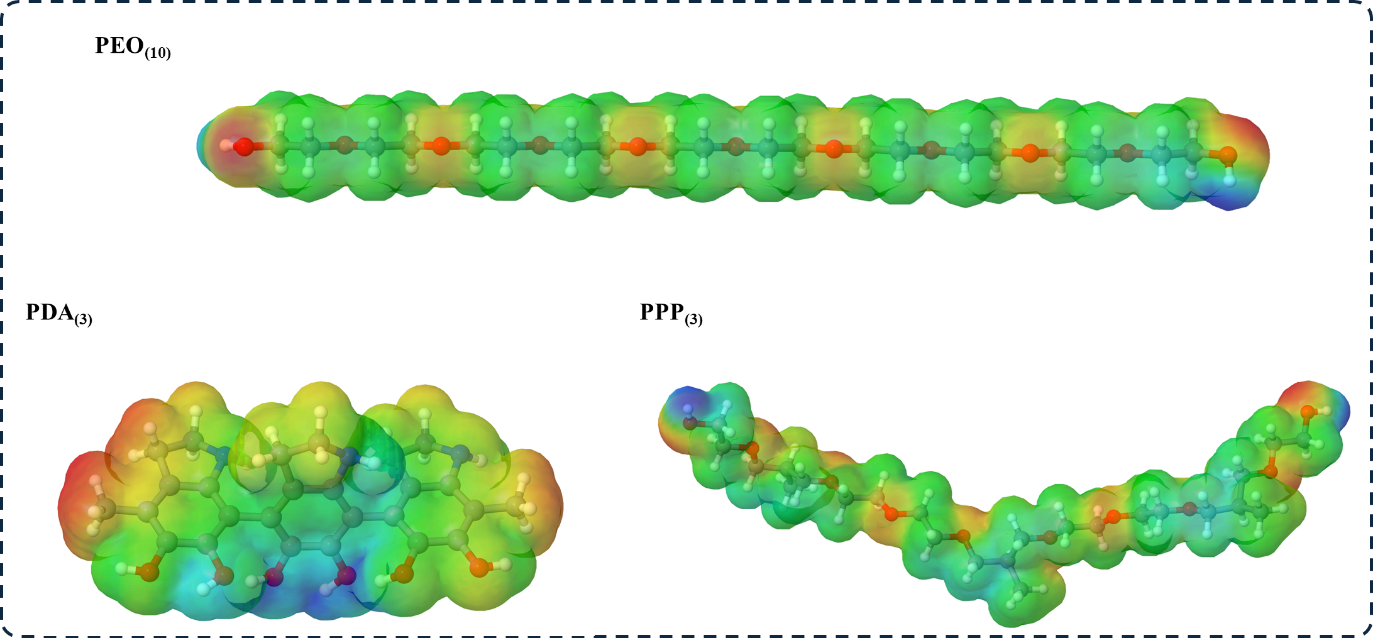


**Figure S10: MEP surfaces of oligomeric structures of PEO, PDA, and PPP.** These maps highlight the preservation of electron-rich coordination centers at ether oxygens and catechol groups, confirming that Li⁺ binding remains governed by local functional groups under polymeric conditions.

**
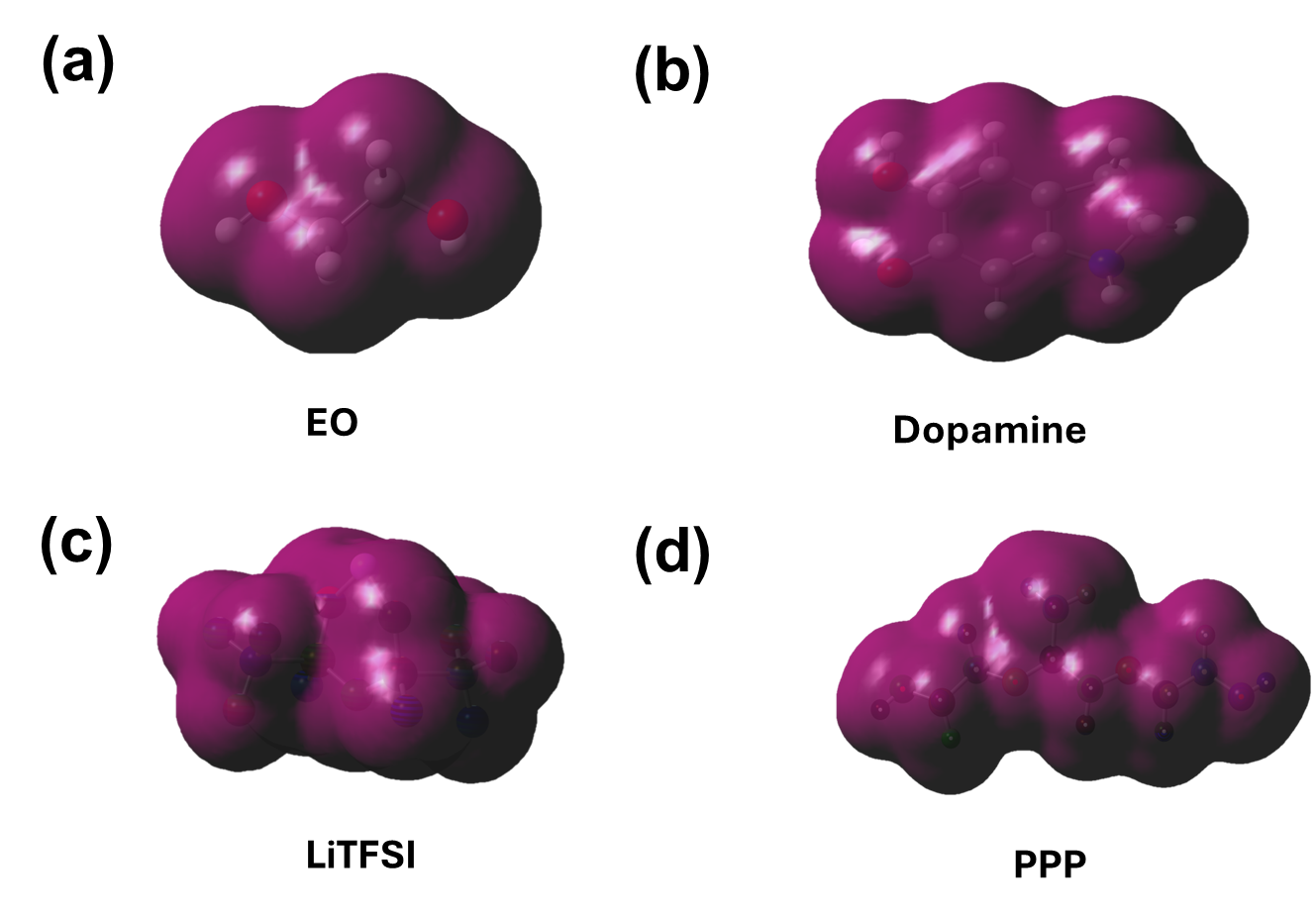
**

**Figure S11: TED maps of EO, Dopamine, LiTFSI and PPP.** These maps visualize electron density distributions, showing extended delocalization along polymer chains and localized density near functional groups, supporting their roles in ion transport and interfacial stability.


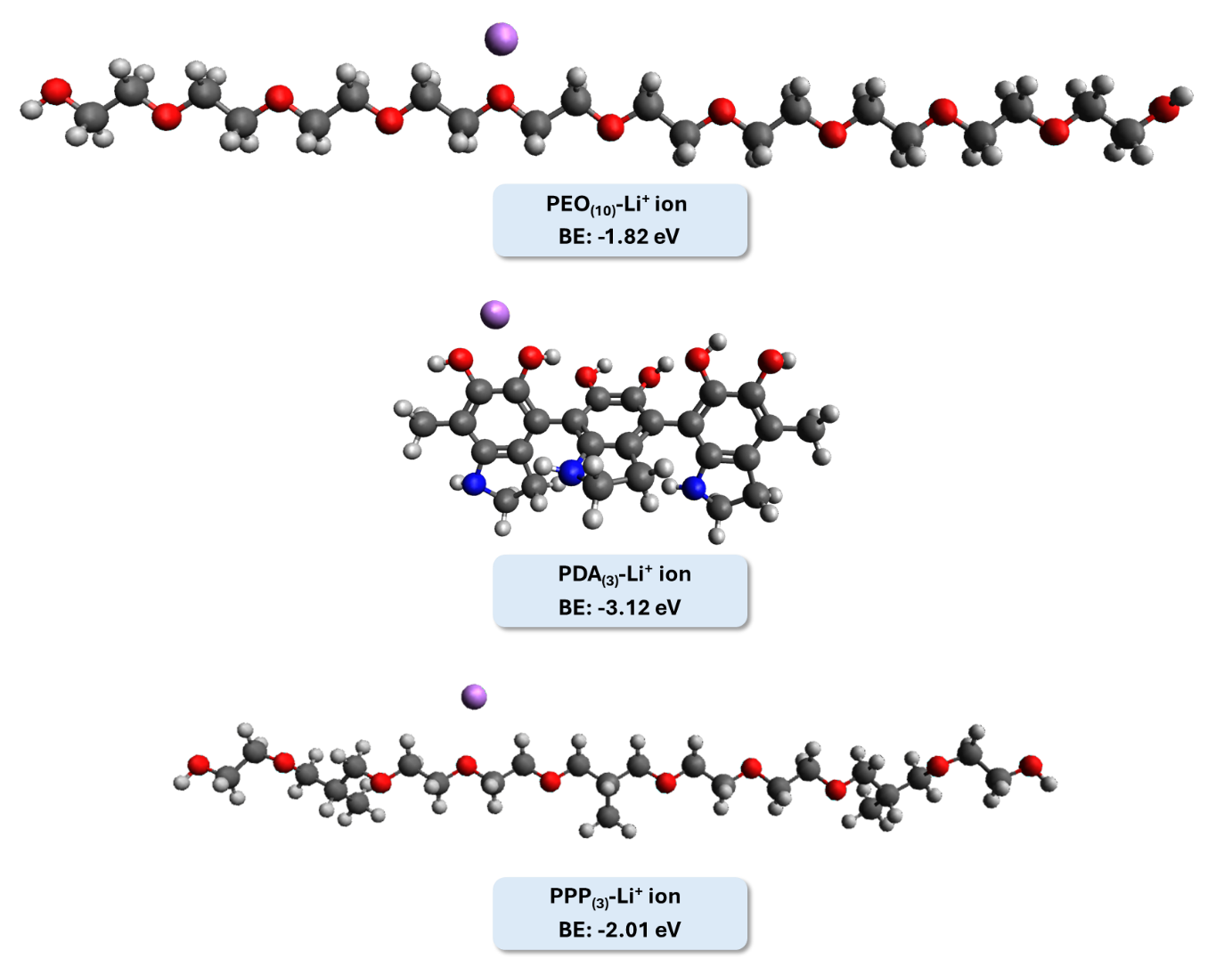


**Figure S12: DFT-optimized Li⁺ coordination configurations for the representative oligomeric models of the electrolyte components, PEO_(10)_, PDA_(3)_, and PPP_(3)_.** The calculated binding energies revealed distinct coordination strengths, with PDA_(3)_ (−3.12 eV) exhibiting the strongest Li⁺ interaction, followed by PPP_(3)_ (−2.01 eV) and PEO_(10)_ (−1.82 eV), reflecting the dominant role of local functional groups in governing Li⁺ binding behavior across the polymeric framework.

**
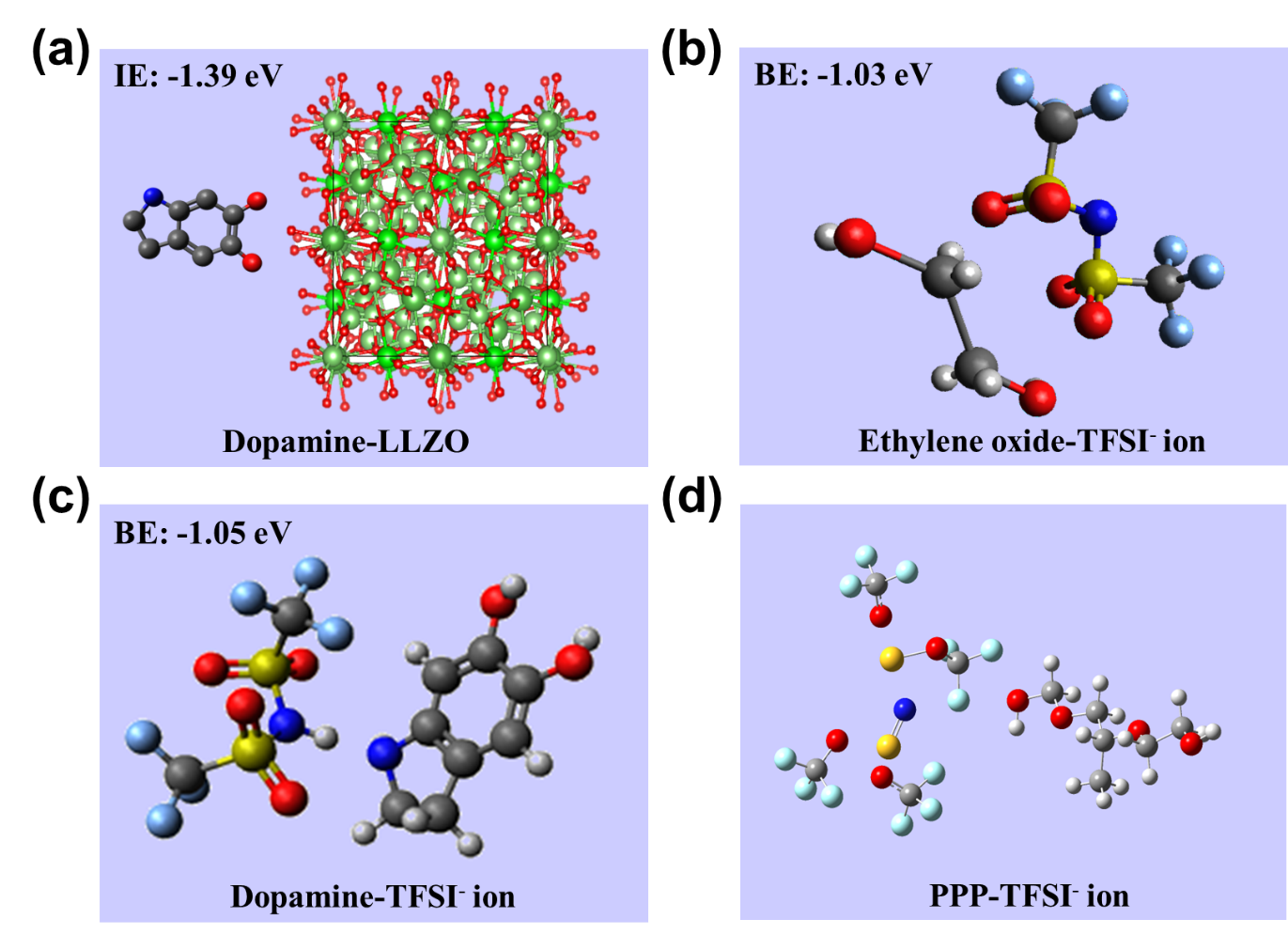
**

**Figure S13: DFT-calculated ion–molecule interaction energy of Dopamine-LLZO, binding energies of EO-TFSI anion, Dopamine-TFSI anion and PPP-TFSI anion.**


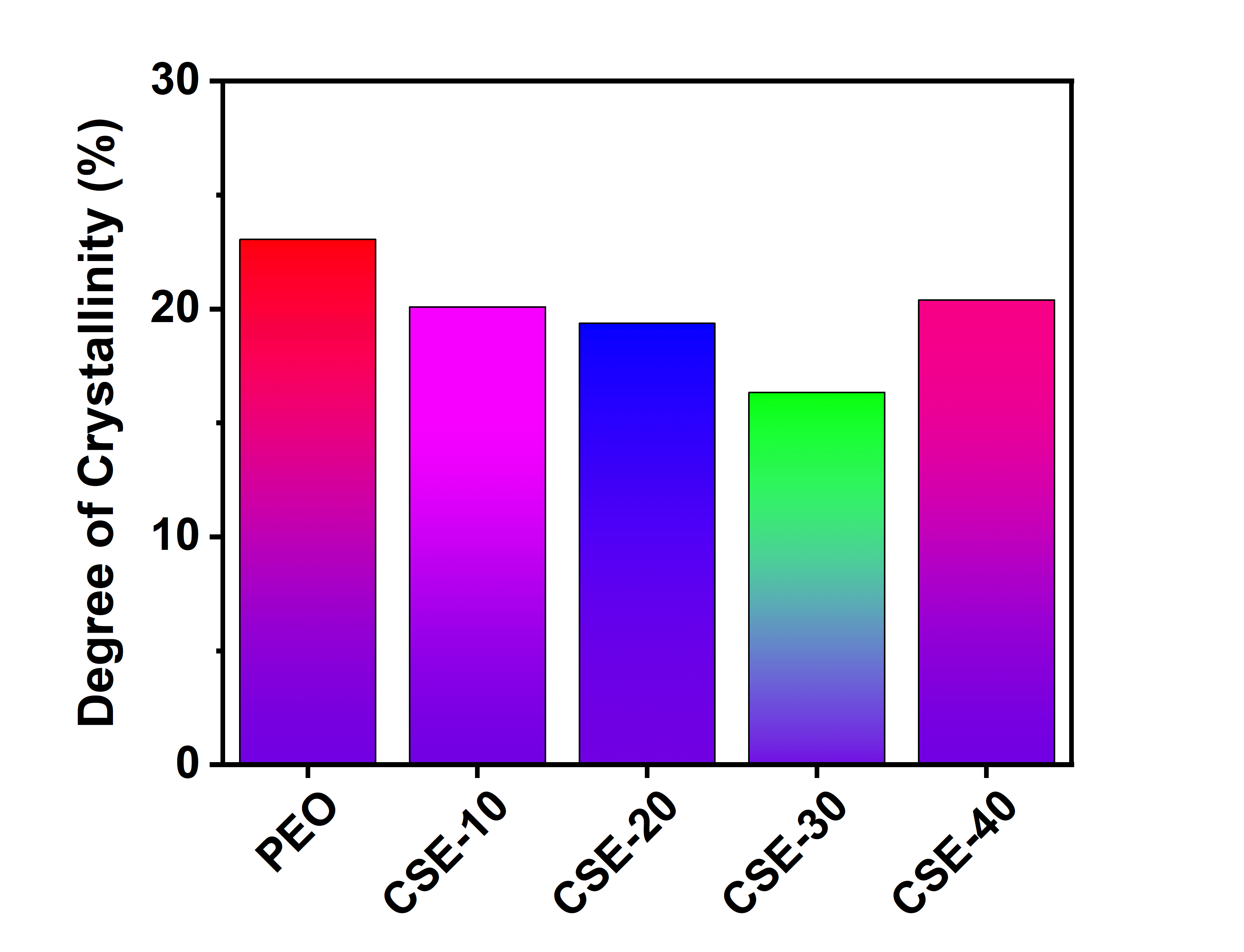


**Figure S14: Degree of crystallinity (%) in pristine PEO and all CSEs, determined from DSC analysis.** CSE-30 exhibits the lowest crystallinity, indicating effective disruption of PEO chain ordering by PDA@LLZO and PPP, which promotes amorphous domain formation for enhanced ion transport.


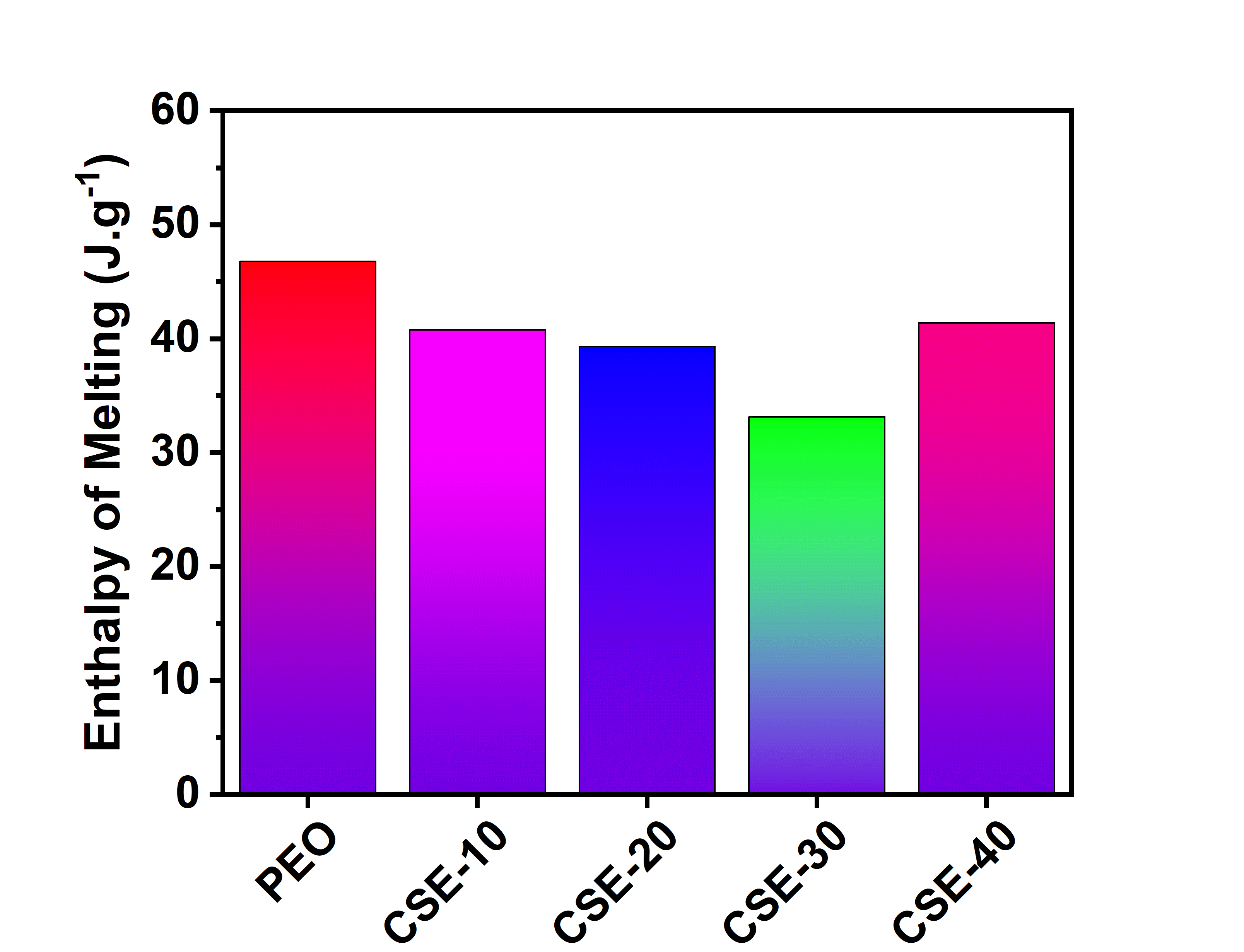


**Figure S15: Melting enthalpy (ΔH) of pristine PEO and composite solid electrolytes (CSE-10 to CSE-40) obtained from DSC analysis.** CSE-30 shows the lowest ΔH, confirming maximal disruption of PEO crystallites due to synergistic interaction with PDA@LLZO and PPP, which enhances amorphous phase content and ion mobility.


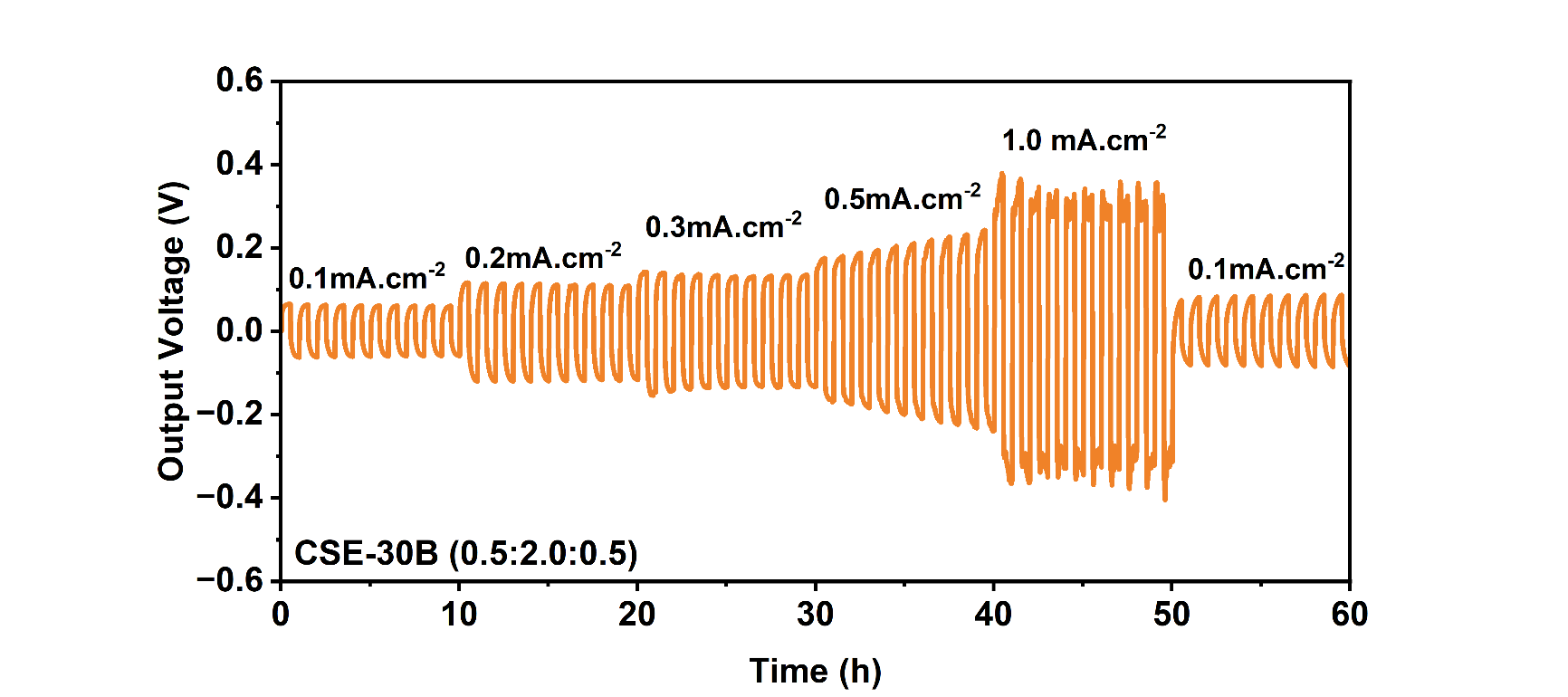


**Figure S16. Galvanostatic Li plating/stripping behavior of the CSE-30B electrolyte (layer thickness ratio 0.5:2:0.5) in a symmetric Li∣CSE-30B∣Li cell at 60 °C.** Stable and symmetric voltage profiles are observed at 0.1–0.3 mA cm⁻², indicating uniform Li deposition and stripping. A pronounced increase in polarization appears at 0.5 mA cm⁻², suggesting growing interfacial resistance and the onset of localized Li accumulation. Further elevation to 1.0 mA cm⁻² triggers a short-circuit event, attributed to dendritic penetration facilitated by the thinner outer layers, underscoring the critical role of layer-thickness balance in maintaining electrochemical stability.


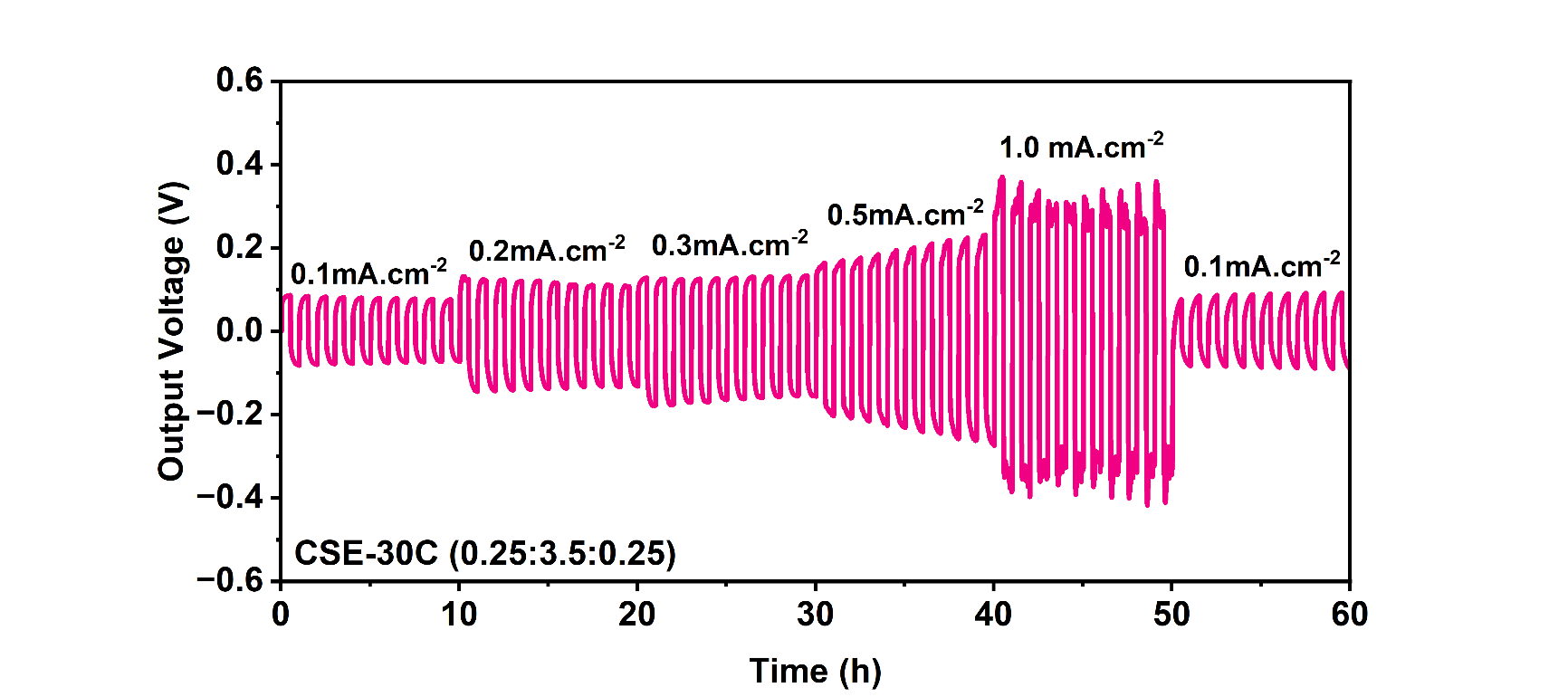


**Figure S17. Galvanostatic Li plating/stripping profile of the CSE-30C electrolyte (layer-thickness ratio 0.25:3.5:0.25) in a symmetric Li∣CSE-30C∣Li cell at 60 °C.** The cell exhibits stable and symmetric voltage oscillations at low current densities (0.1–0.3 mA cm⁻²), indicating uniform Li deposition and stripping. As the current density increases to 0.5 mA cm⁻², the polarization amplitude rises, suggesting increased interfacial resistance and incipient Li accumulation. Further elevation to 1.0 mA cm⁻² leads to rapid voltage decay and short-circuit failure, confirming that excessive thinning of the outer layers diminishes mechanical blocking strength and allows dendritic penetration through the electrolyte despite the high ionic conductivity of the central layer.


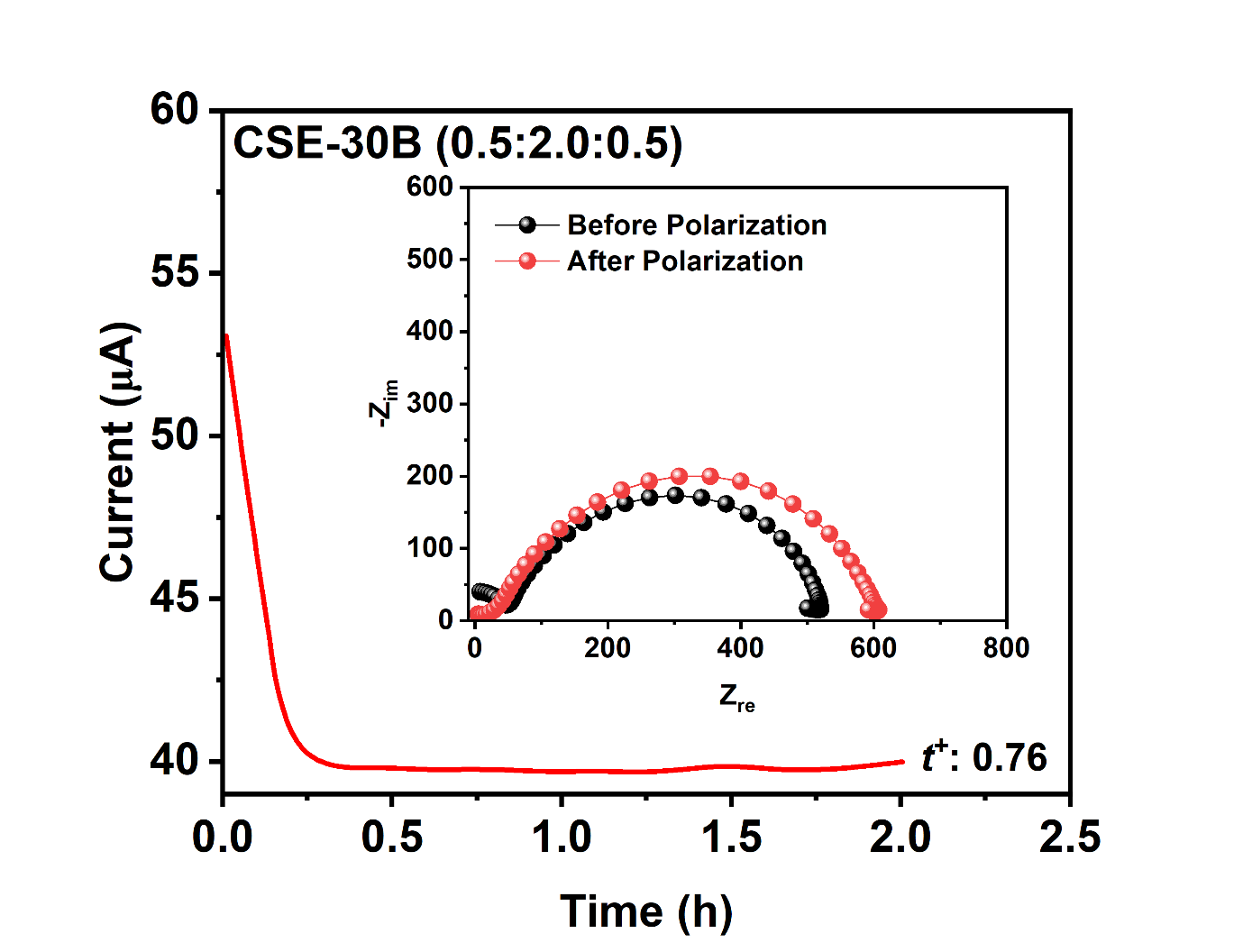


**Figure S18: Li⁺ transference number measurement for CSE-30B (0.5:2:0.5), yielding a calculated t⁺ of 0.76.** The corresponding Nyquist plots recorded before and after DC polarization reveal a moderate increase in interfacial resistance, confirming stable Li⁺ transport across the electrolyte/electrode interface.


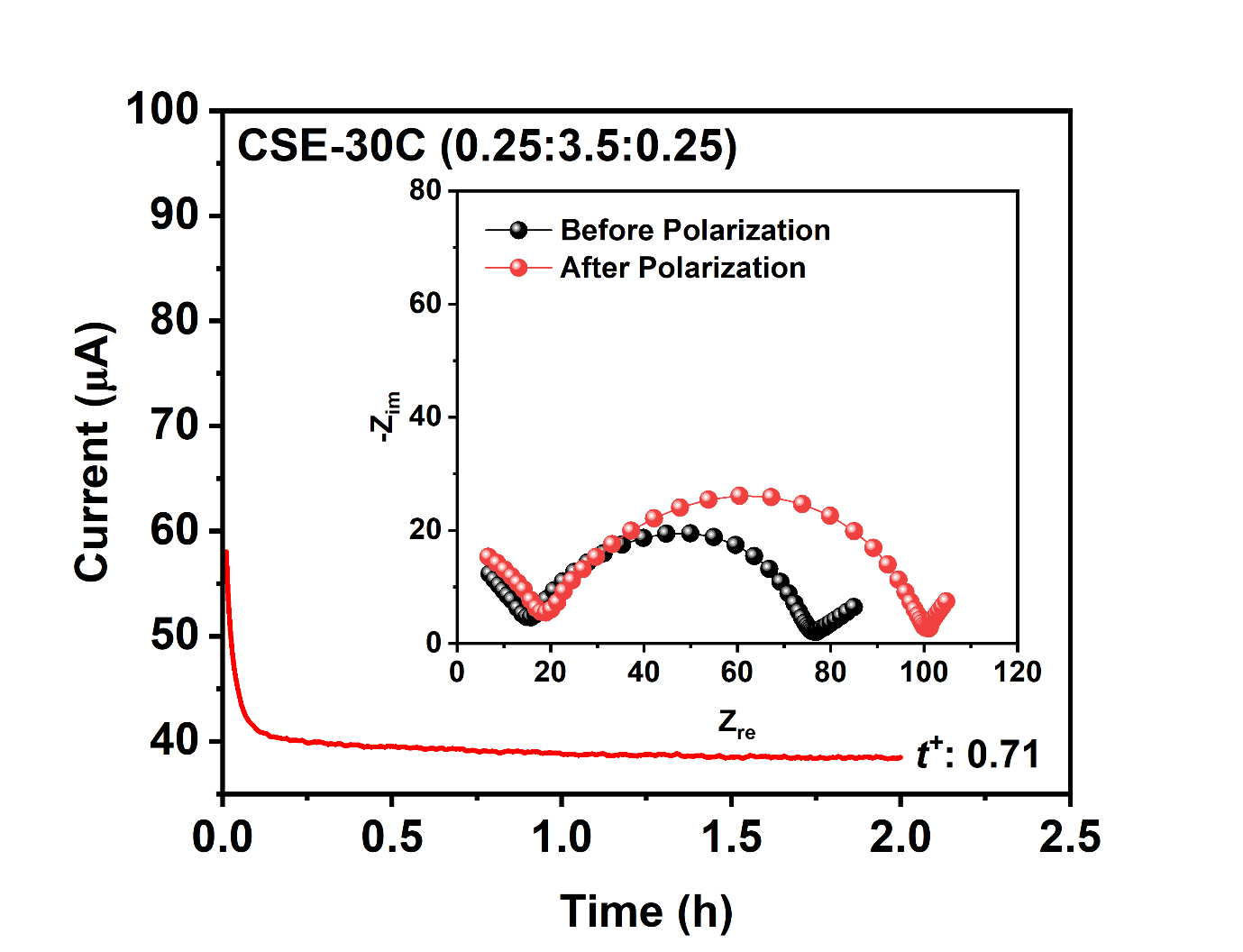


**Figure S19: Li⁺ transference number measurement for CSE-30C indicating a calculated t_Li_^+^ of 0.71.** Nyquist plots before and after polarization highlight moderate interfacial resistance growth, confirming stable ion conduction.


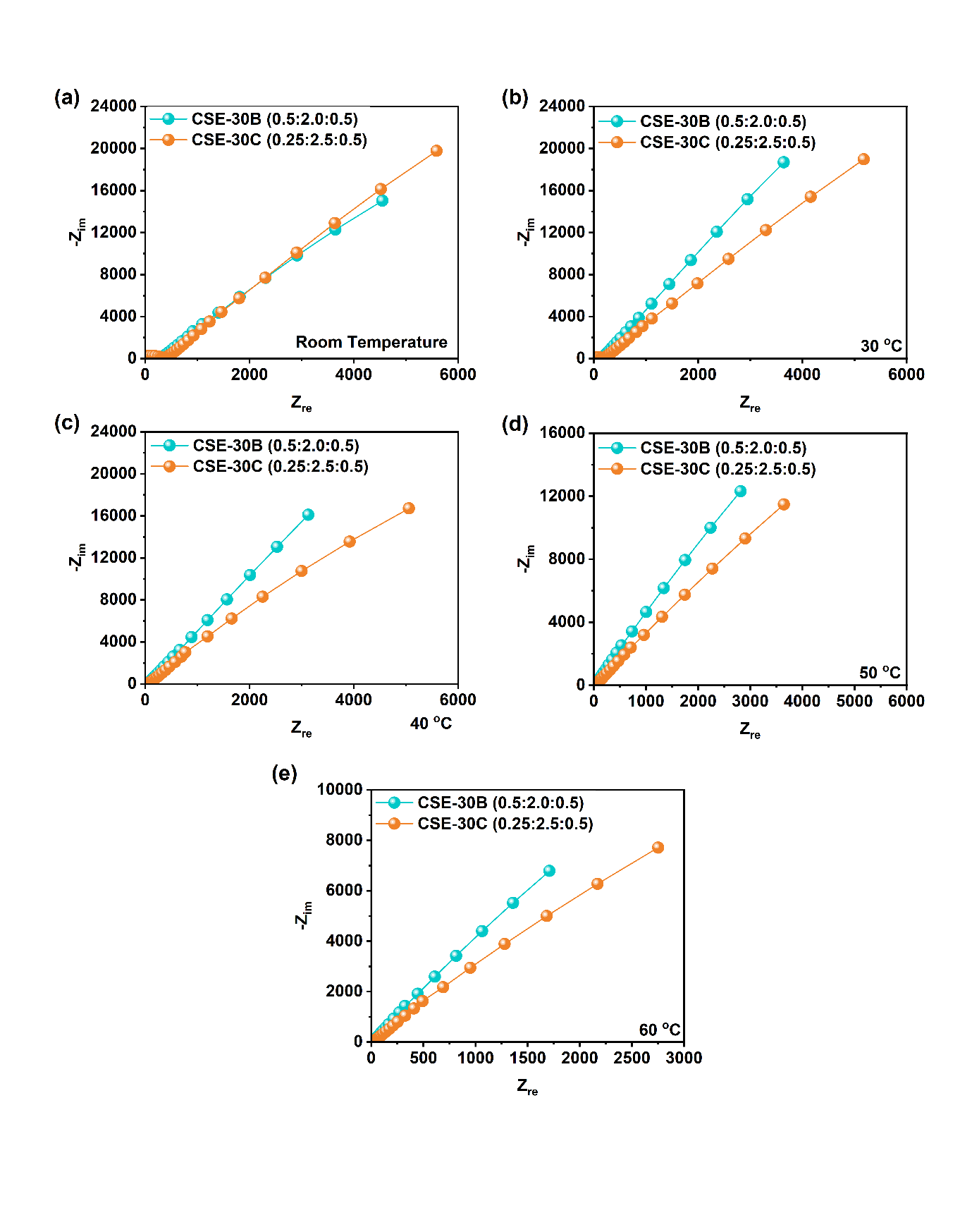


**Figure S20: Nyquist plots of CSE-30 and its variants (CSE-30 B & CSE-30C) at (a) Room temperature, (b) 30 ^o^C, (c) 40 ^o^C, (d) 50 ^o^C, and (e) 60 ^o^C.**


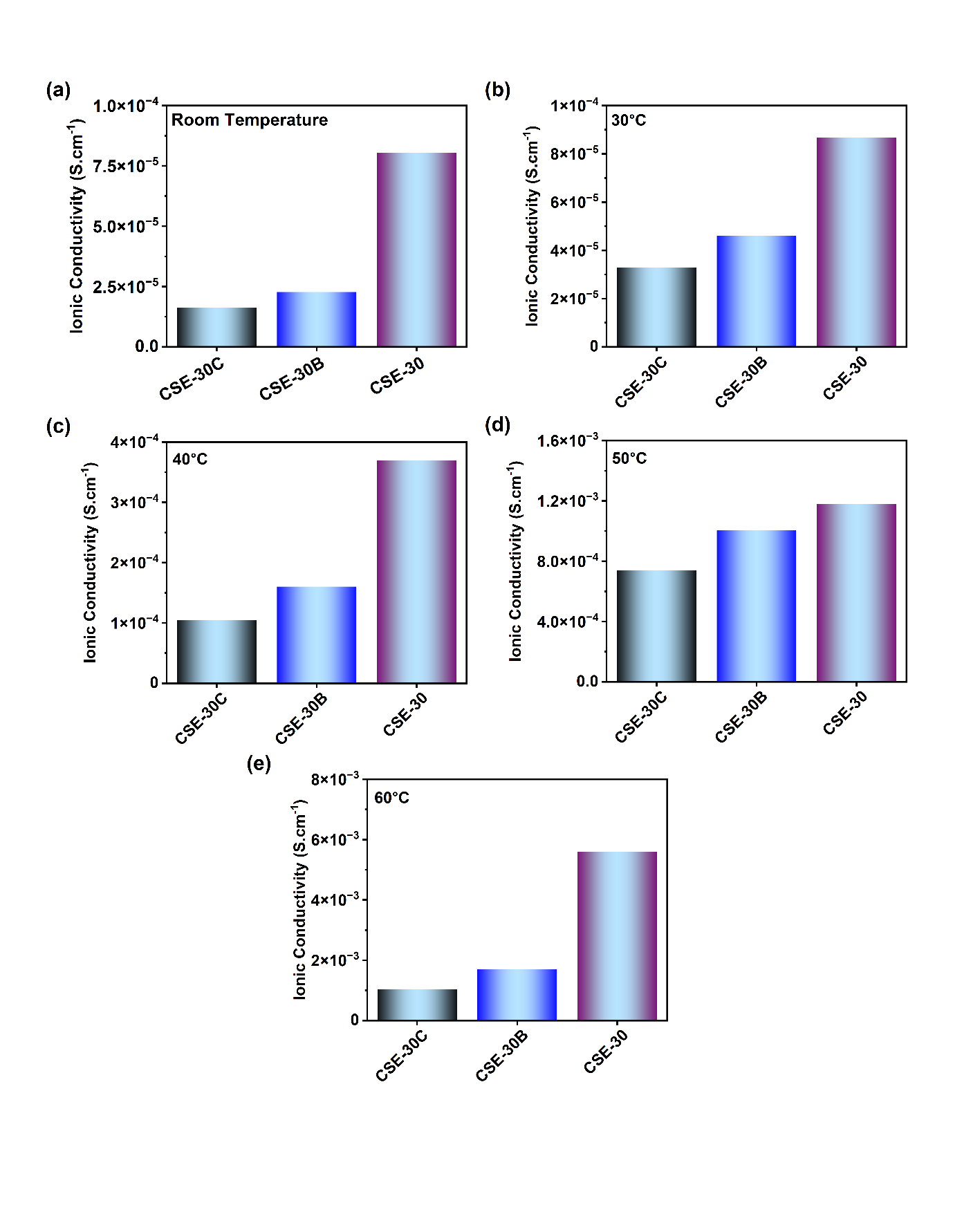


**Figure S21: Ionic conductivity trends of CSE-30 and its variants (CSE-30 B & CSE-30C) at (a) Room temperature, (b) 30 ^o^C, (c) 40 ^o^C, (d) 50 ^o^C, and (e) 60 ^o^C.**

**Table S3: Temperature-dependent Ionic conductivities of CSE-30 and its variants (CSE-30 B & CSE-30C)**

| **Electrolyte** | **Ionic Conductivity (S.cm^-1^)** | | | | |
| --- | --- | --- | --- | --- | --- |
|  | **RT** | **30 ^o^C** | **40 ^o^C** | **50 ^o^C** | **60 ^o^C** |
| **CSE-30C** | 1.63 × 10^-5^ | 3.30 × 10^-5^ | 1.05× 10^-4^ | 7.40 × 10^-4^ | 1.05 × 10^-3^ |
| **CSE-30B** | 2.28 × 10^-5^ | 4.61 × 10^-5^ | 1.61× 10^-4^ | 1.01 × 10^-3^ | 1.70 × 10^-3^ |
| **CSE-30** | 8.0 × 10^-5^ | 8.67 × 10^-5^ | 3.69× 10^-4^ | 1.18 × 10^-3^ | 5.60 × 10^-3^ |


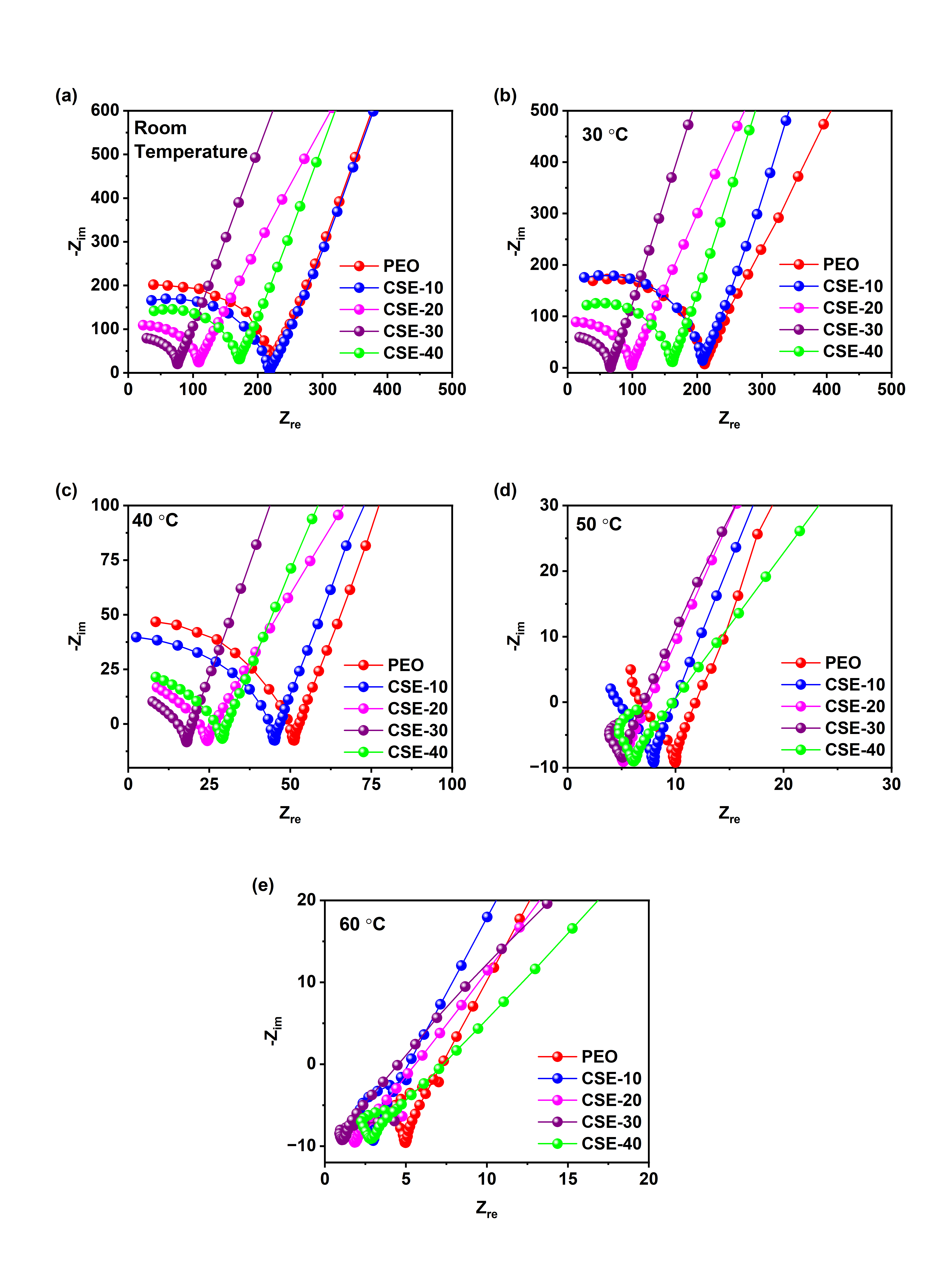
**Figure S22: Nyquist plots of PEO, CSE-10, CSE-20, CSE-30 & CSE-40 at various temperatures**


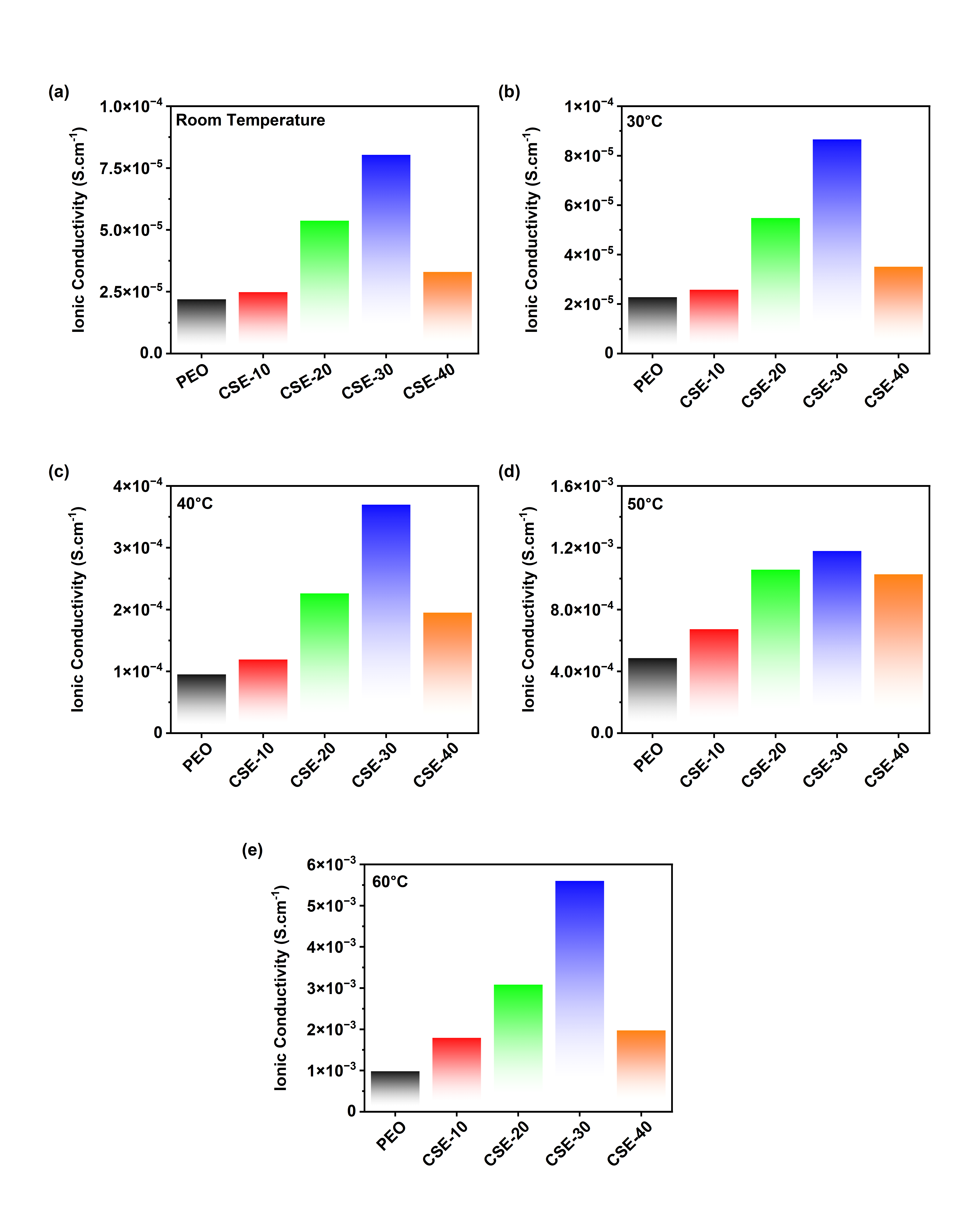


**Figure S23: Ionic conductivity trends of PEO, CSE-10, CSE-20, CSE-30 and CSE-40 at (a) Room temperature, (b) 30 ^o^C, (c) 40 ^o^C, (d) 50 ^o^C, and (e) 60 ^o^C.**

**Table S4: Temperature-dependent ionic conductivities and corresponding activation energies (Ea) of pristine PEO and CSEs with varying PDA@LLZO and PPP content.**

| **Electrolyte** | **Ionic Conductivity (S.cm^-1^)** | | | | | **E_a_**  **(eV)** |
| --- | --- | --- | --- | --- | --- | --- |
|  | **RT** | **30 ^o^C** | **40 ^o^C** | **50 ^o^C** | **60 ^o^C** |  |
| **PEO** | 2.20 × 10^-5^ | 2.29 × 10^-5^ | 9.55× 10^-5^ | 4.87 × 10^-4^ | 9.88 × 10^-4^ | 0.97 |
| **CSE-10** | 2.0 × 10^-5^ | 2.59 × 10^-5^ | 1.19× 10^-4^ | 6.74 × 10^-4^ | 1.80 × 10^-3^ | 1.14 |
| **CSE-20** | 5.0 × 10^-5^ | 5.49 × 10^-5^ | 2.26× 10^-4^ | 1.06 × 10^-3^ | 3.09 × 10^-3^ | 1.06 |
| **CSE-30** | 8.0 × 10^-5^ | 8.67 × 10^-5^ | 3.69× 10^-4^ | 1.18 × 10^-3^ | 5.60 × 10^-3^ | 1.07 |
| **CSE-40** | 3.0 × 10^-5^ | 3.52 × 10^-5^ | 1.95× 10^-4^ | 1.03 × 10^-3^ | 1.98 × 10^-3^ | 1.01 |


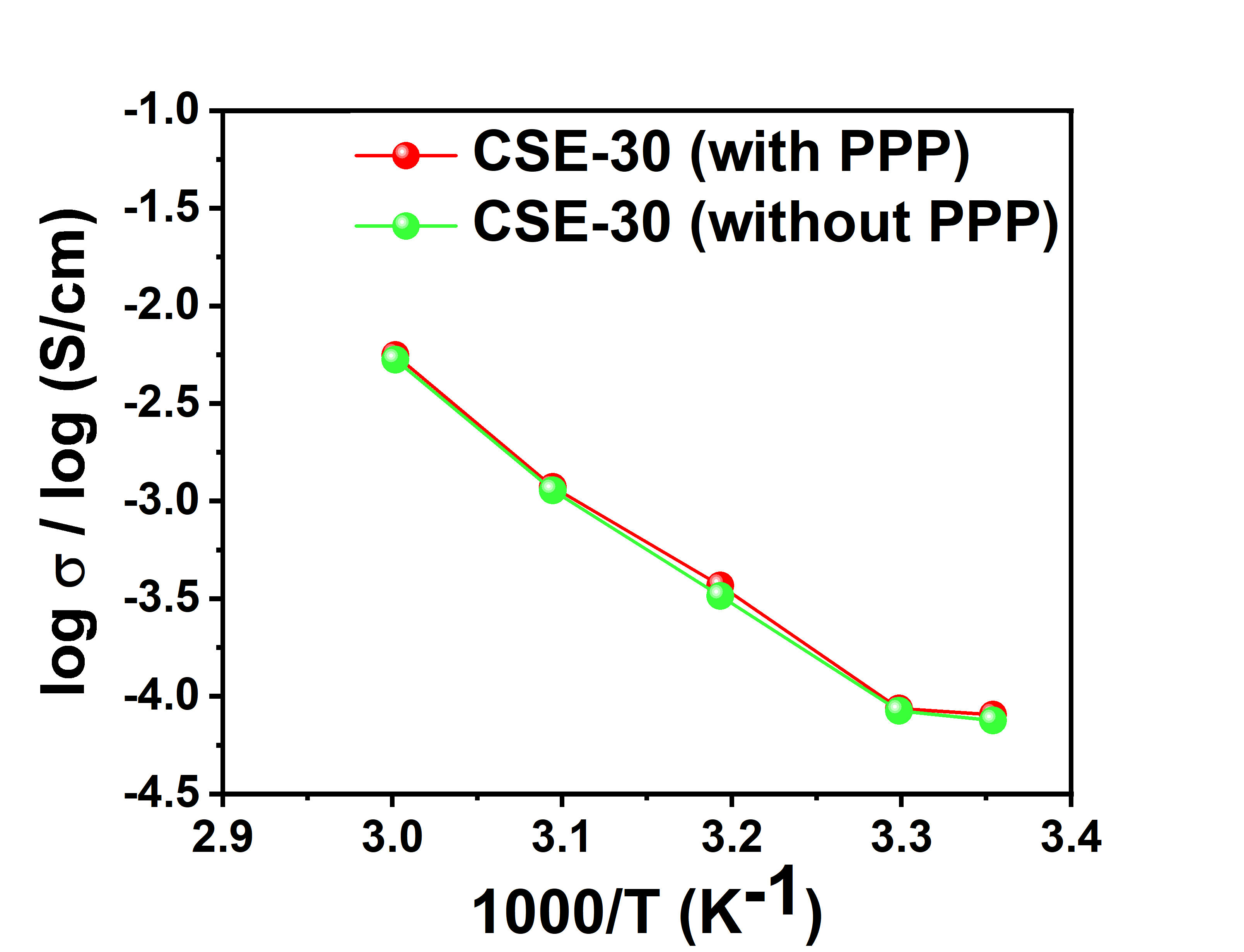


**Figure S24: Arrhenius plots comparing ionic conductivity of CSE-30 with and without PPP segments.** The incorporation of PPP enhances ionic conductivity across all temperatures by promoting polymer segmental motion and reducing ion transport barriers.


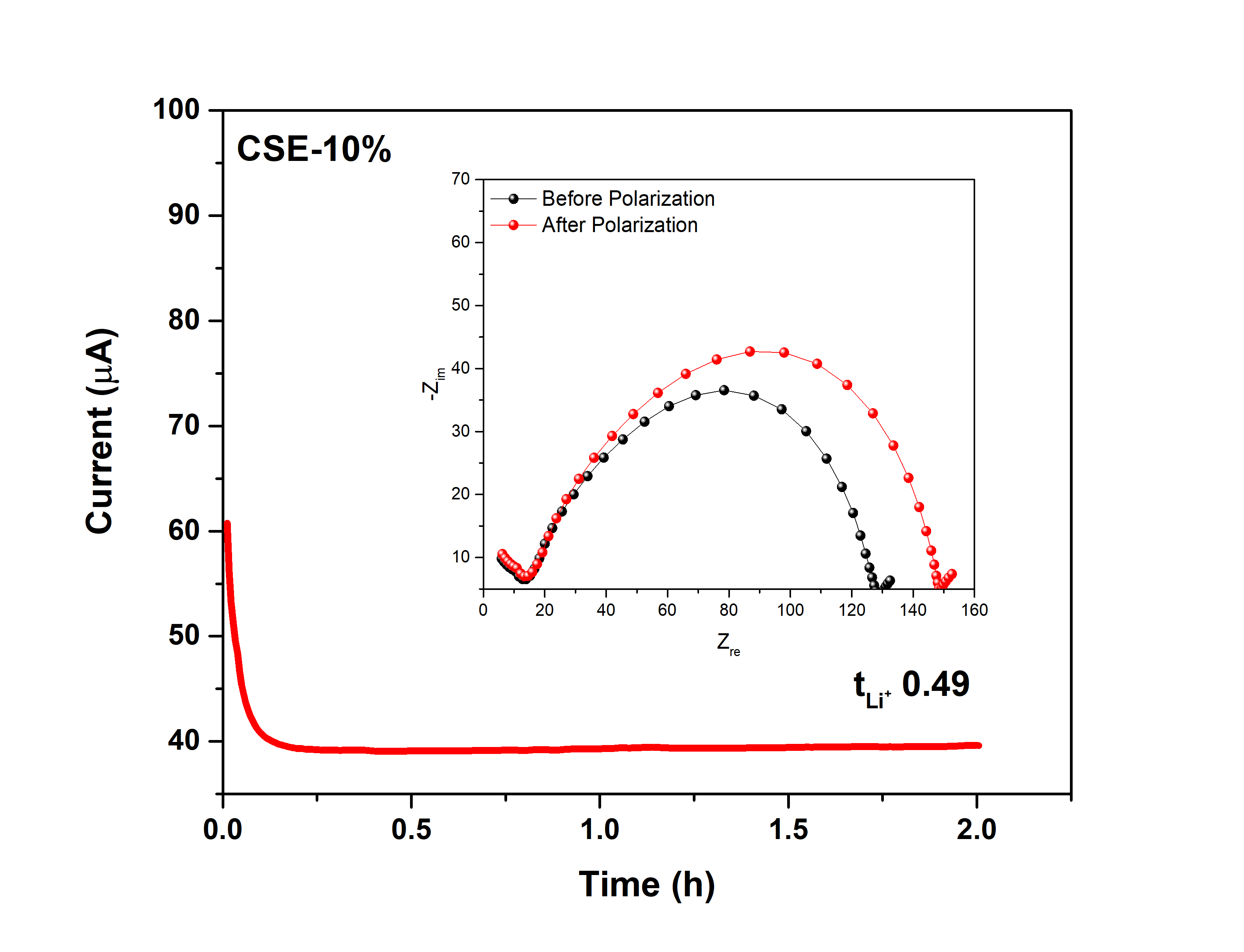


**Figure S25: Li⁺ transference number measurement of CSE-10 showing a stabilized current profile and calculated t_Li_^+^​ of 0.49.** Nyquist plots before and after DC polarization reveal moderate changes in interfacial resistance, indicating stable Li⁺ conduction dynamics.


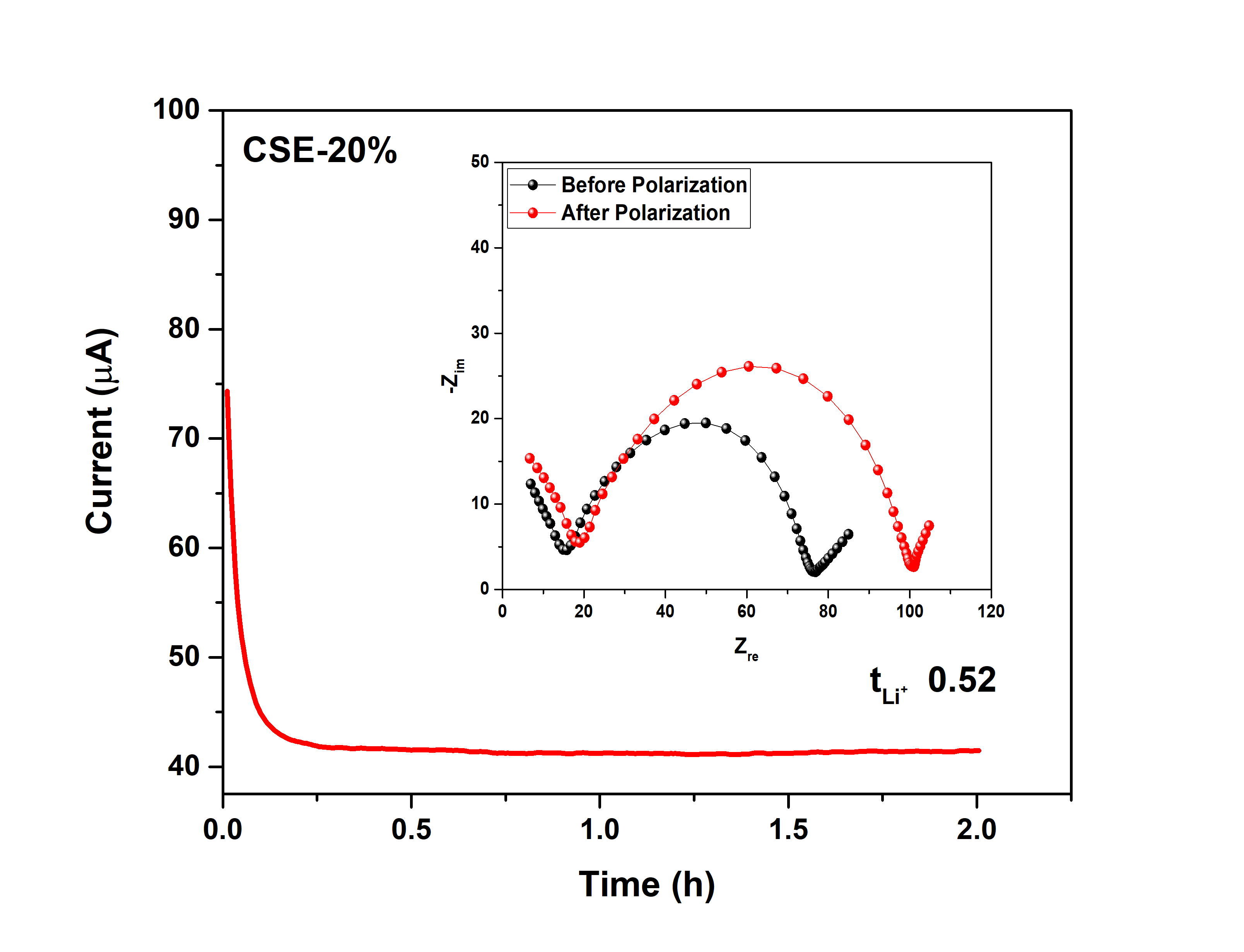


**Figure S26: Li⁺ transference number measurement for CSE-20 indicating a steady-state current profile and a calculated t_Li_^+^ of 0.52.** Nyquist plots before and after polarization highlight moderate interfacial resistance growth, confirming stable ion conduction.


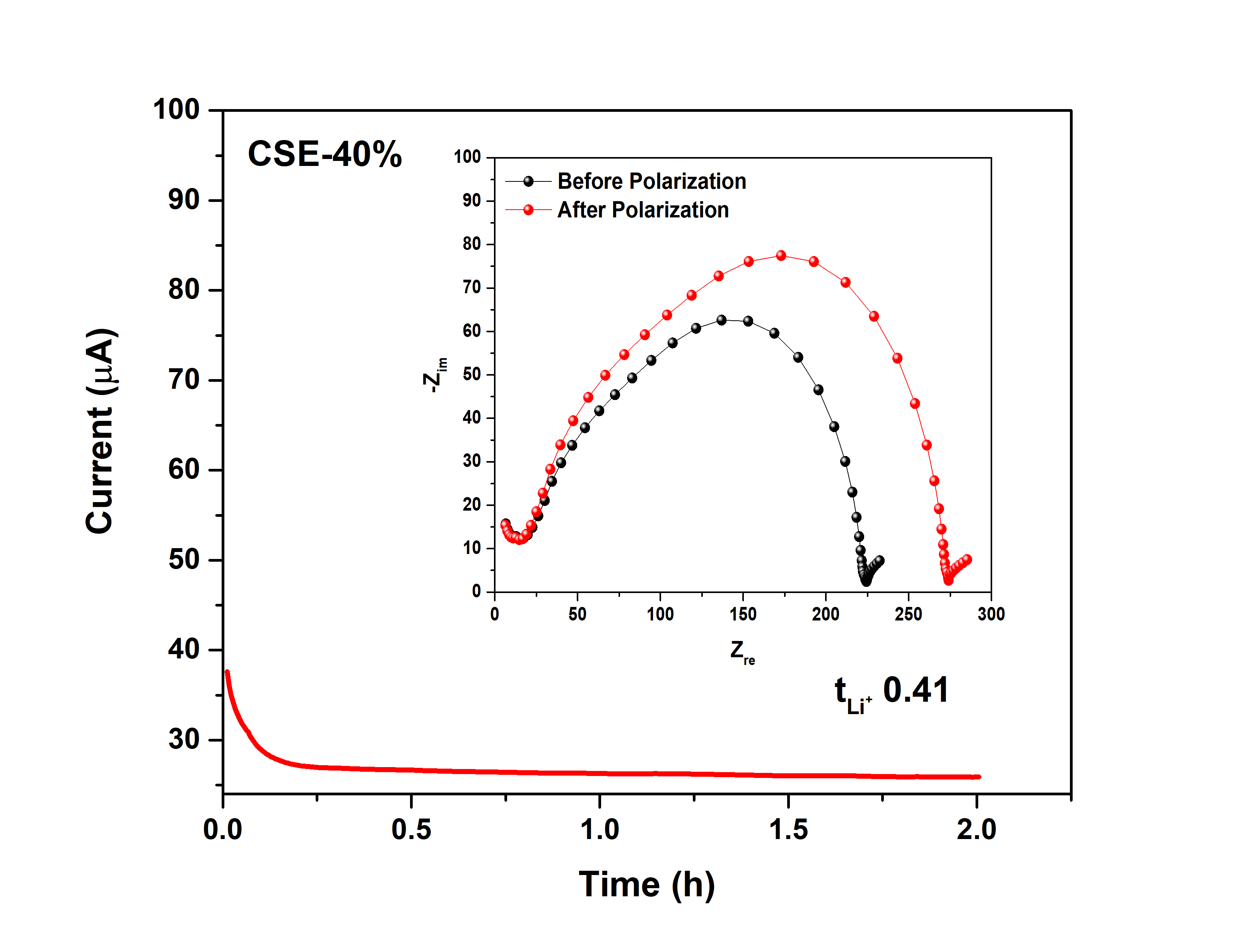


**Figure S27:** **Li⁺ transference number measurement for CSE-40 indicating a declined current profile and a calculated t_Li_^+^ of 0.41.** Nyquist plots before and after polarization highlight increased interfacial resistance growth, confirming unstable ion conduction.


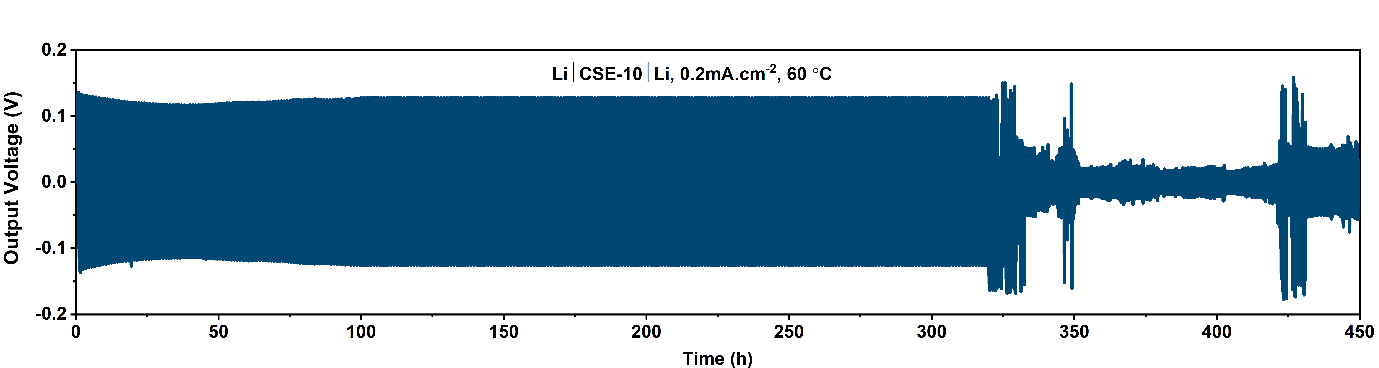


**Figure S28:** **Long-term Li stripping/plating performance of CSE-10.** Symmetric Li‖Li cells using CSE-10 were cycled at 0.2 mA cm⁻² and 60 °C, showing stable voltage profiles up to ~300 h before increased polarization and short-circuit features emerged. The prolonged cycling confirms the initial interfacial stability and mechanical resistance of the CSE-10 membrane under moderate current density.


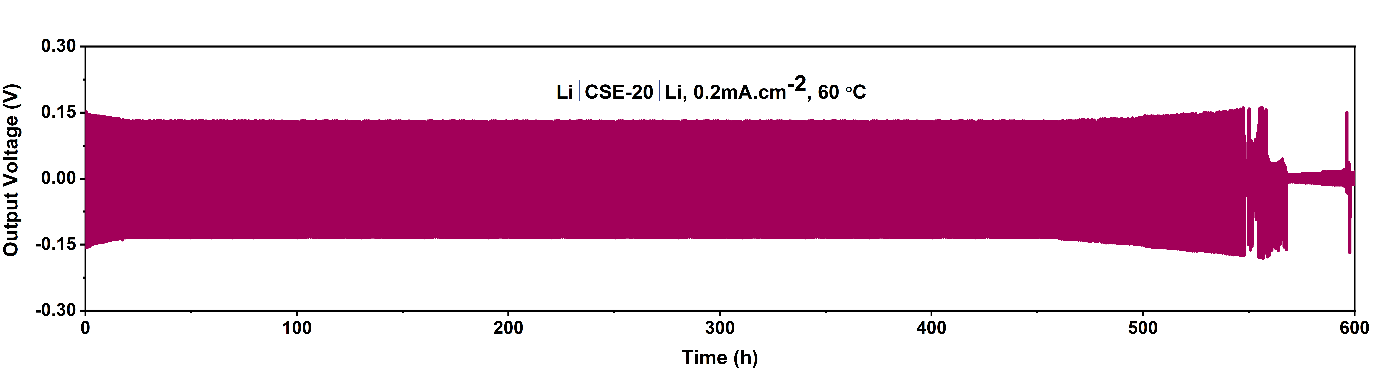


**Figure S29: Voltage stability of Li‖Li symmetric cells using CSE-20 electrolyte under prolonged cycling.** The cell operated at 0.2 mA cm⁻² and 60 °C maintained low polarization with stable Li stripping/plating over ~500 h, demonstrating superior interfacial and mechanical stability compared to CSE-10 before eventual short-circuit onset.


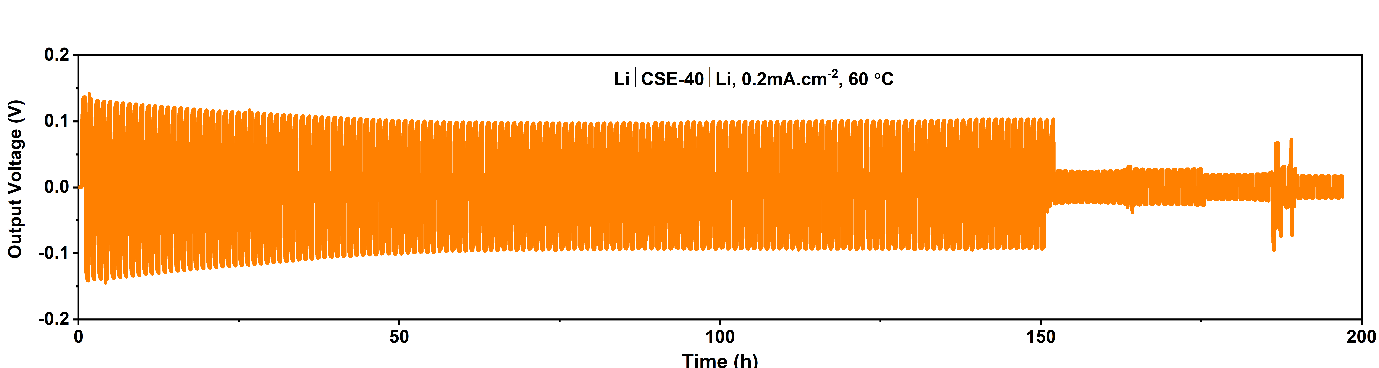


**Figure S30:** **Electrochemical cycling performance of Li‖Li symmetric cells with CSE-40 at elevated temperature.** The cell exhibited increasing polarization and early instability during Li stripping/plating at 0.2 mA cm⁻² and 60 °C, with clear signs of failure after ~160 h, indicating compromised interfacial stability and possible mechanical limitations at higher filler loading.


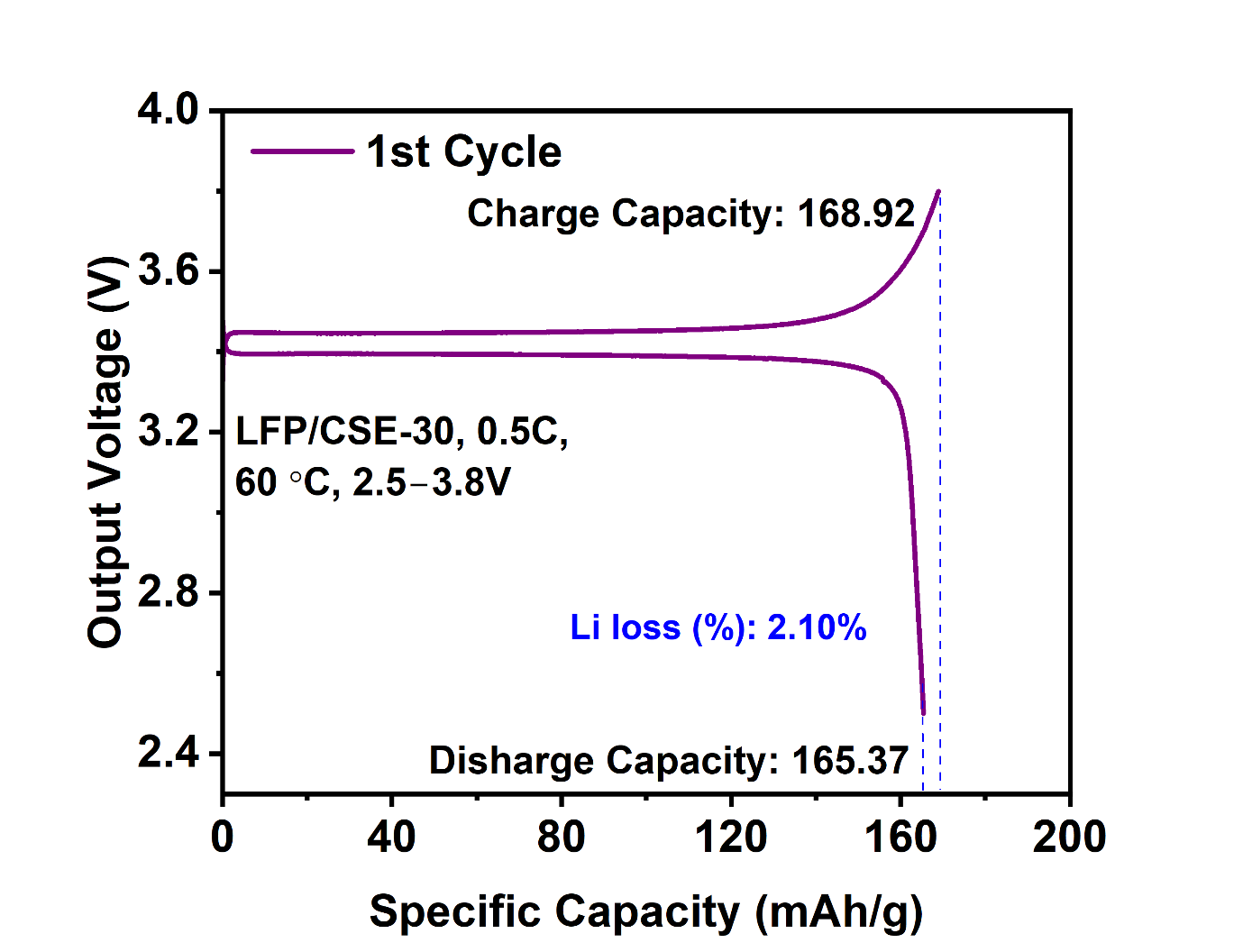


**Figure S31: First-cycle charge–discharge profile of the LFP‖CSE-30 full cell recorded at 0.5C and 60 °C within 2.5–3.8 V.** The cell delivers a charge capacity of 168.92 mAh g⁻¹ and a discharge capacity of 165.37 mAh g⁻¹, corresponding to a 2.10 % Li loss and a Coulombic efficiency of 97.9 %. This minimal irreversible capacity confirms efficient Li utilization and stable interfacial formation during the initial activation process.


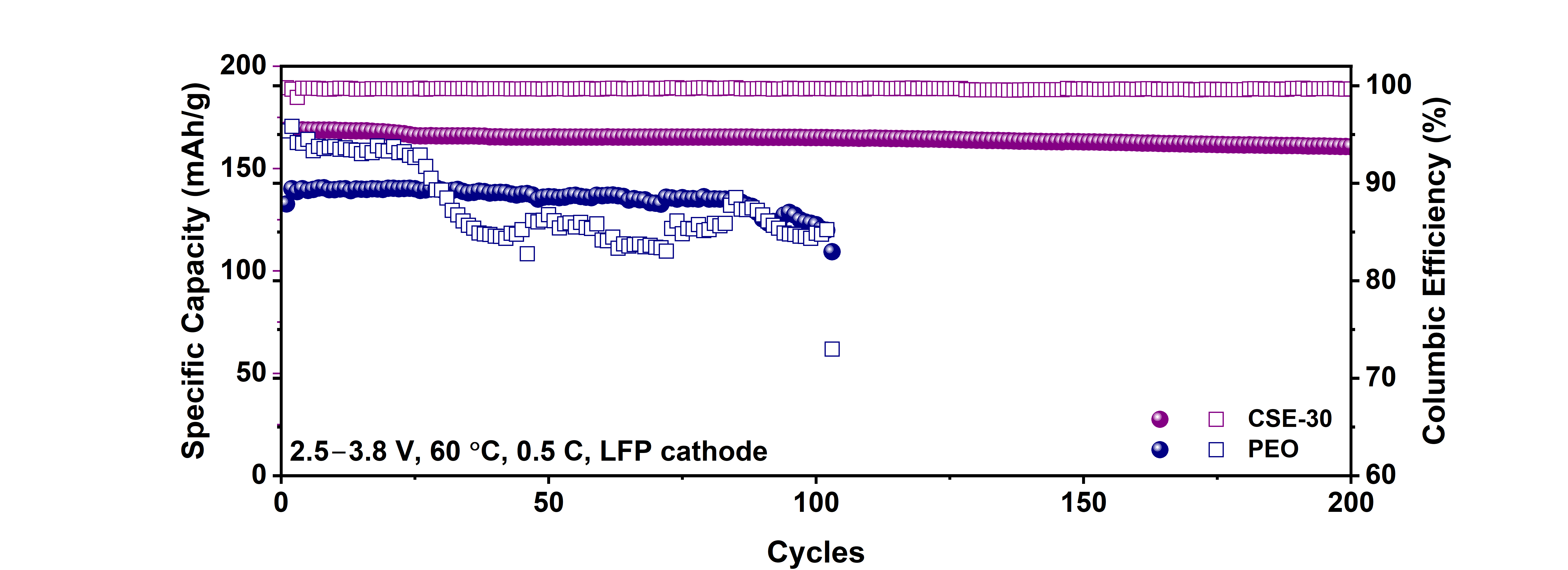


**Figure S32: Cycling performance comparison of Li‖LFP full cells at first 200 cycles using CSE-30 and PEO electrolytes at 0.5C and 60 °C.** The CSE-30-based cell exhibits outstanding capacity retention (165 mAh g⁻¹) and stable Coulombic efficiency (99%) over 200 cycles, reflecting robust interfacial stability. In contrast, the PEO-based cell shows rapid capacity fading and significant decline in efficiency, failing before 120 cycles due to interfacial degradation and poor Li⁺ transport kinetics.


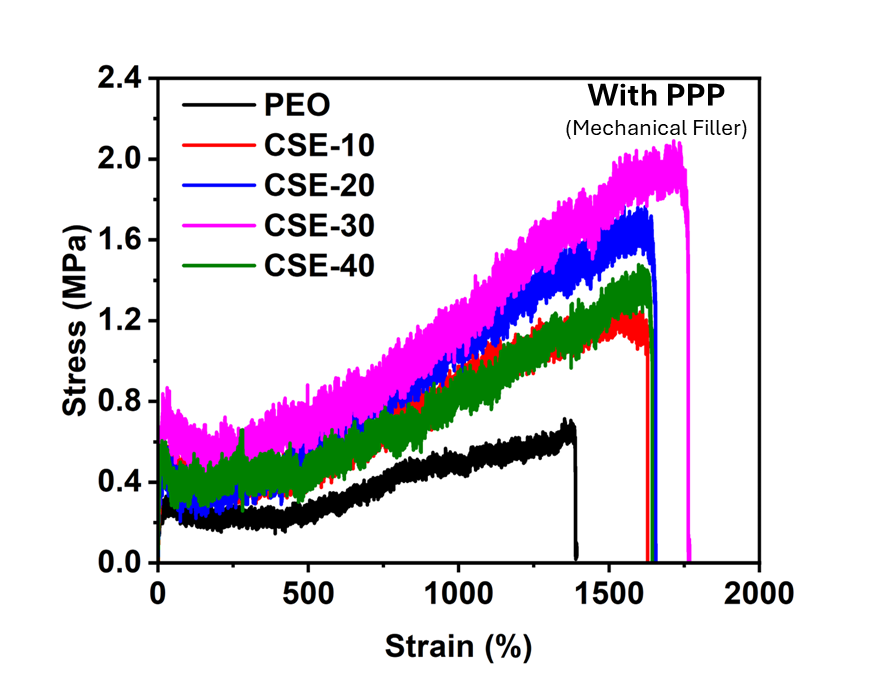


**Figure S33:** **Tensile stress–strain curves of PEO and CSEs incorporating PDA-coated LLZO and PPP as a mechanical filler.** The incorporation of PPP significantly enhances mechanical strength and ductility, with CSE-30 showing the highest tensile performance.


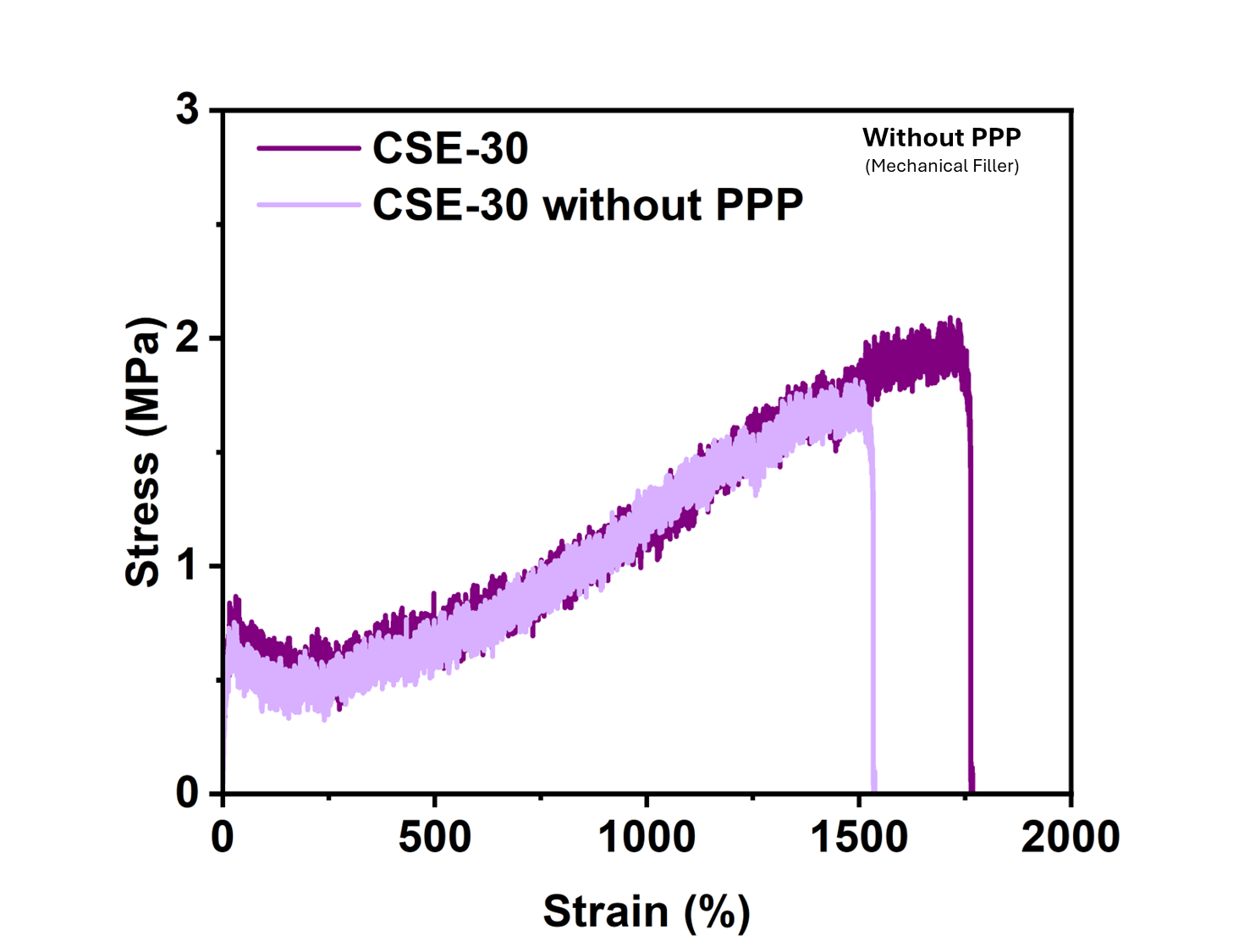


**Figure S34:** **Tensile stress–strain curves of CSE-30 with and without PPP as a mechanical filler.** The incorporation of PPP significantly enhances mechanical strength and ductility, showing the highest tensile performance.


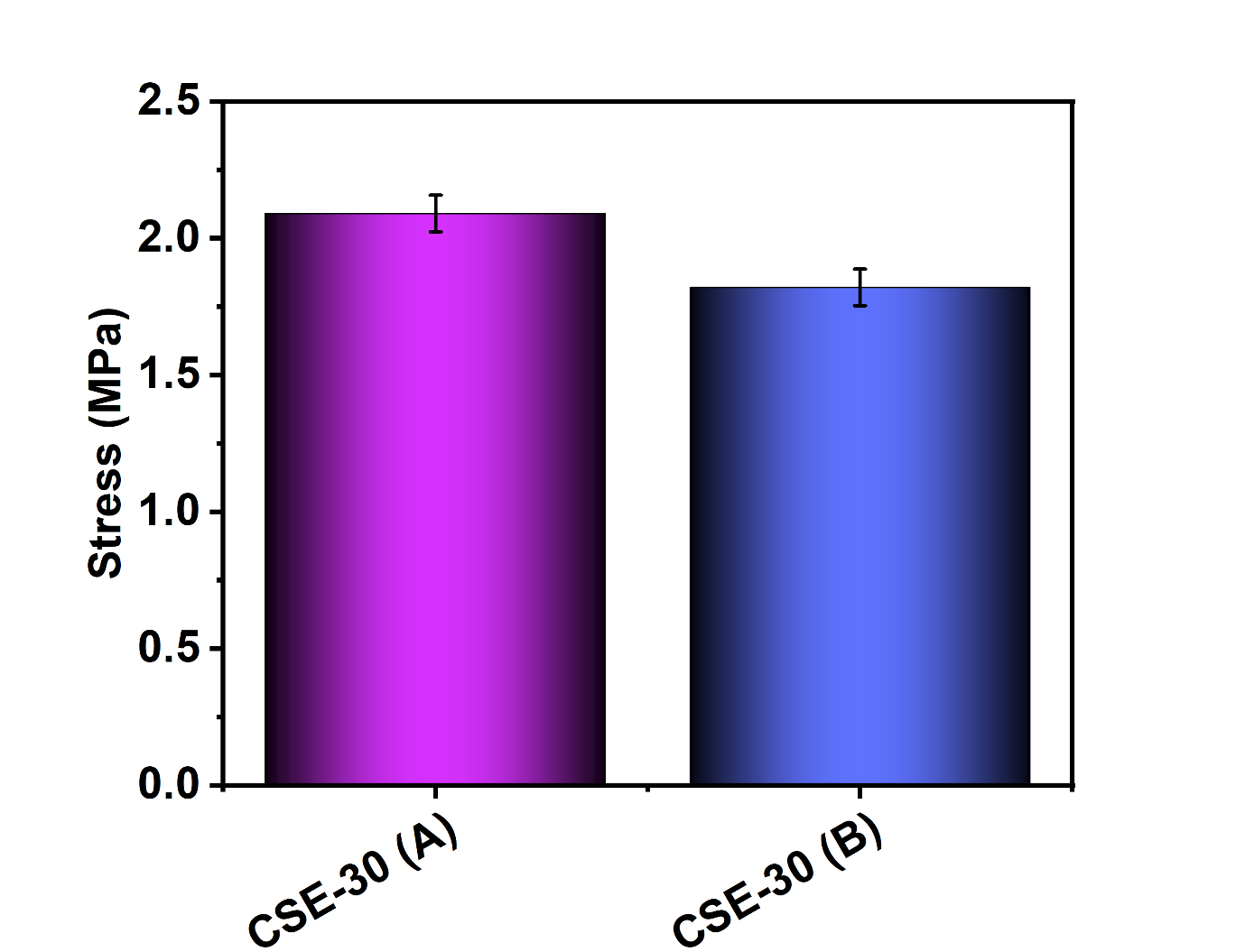


**Figure S35: Mechanical Stress of CSE-30 (A) with mechanical filler PPP and CSE-30 (B) without mechanical filler PPP.** CSE-30 (A) showing the highest stress (2.09 MPa), confirming optimal reinforcement caused by PPP.


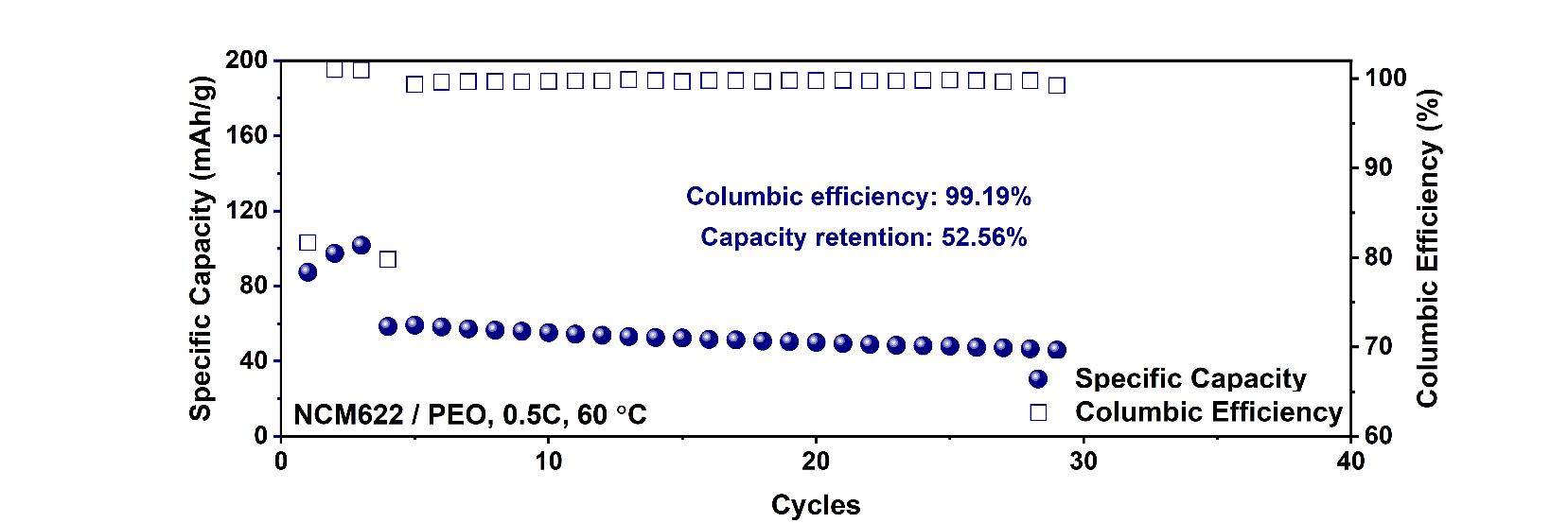


**Figure S36:** **Electrochemical performance of PEO full cell.** NCM622‖PEO‖Li full cell shows 52.56% capacity retention after 29 cycles, revealing pronounced capacity fading and limited long-term cycling stability.


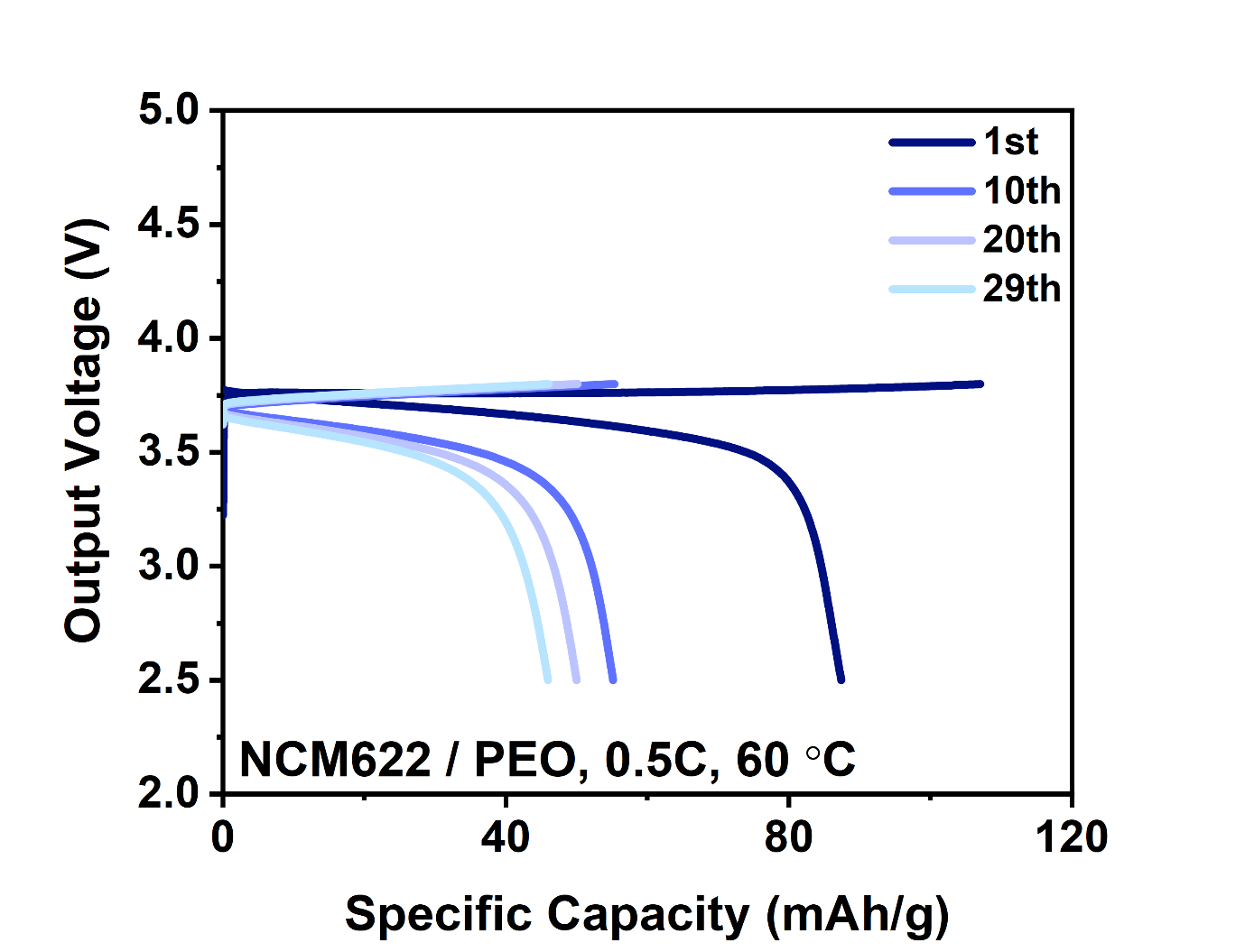


**Figure S37. Galvanostatic charge–discharge (GCD) profiles of PEO-based full cell (Li‖NCM622) at 0.5 C and 60 °C.** The cell shows pronounced and continuous capacity decay with cycling, accompanied by increasing polarization, as evidenced by progressive shift and distortion of voltage profiles, ultimately leading to failure by the 29^th^ cycle.


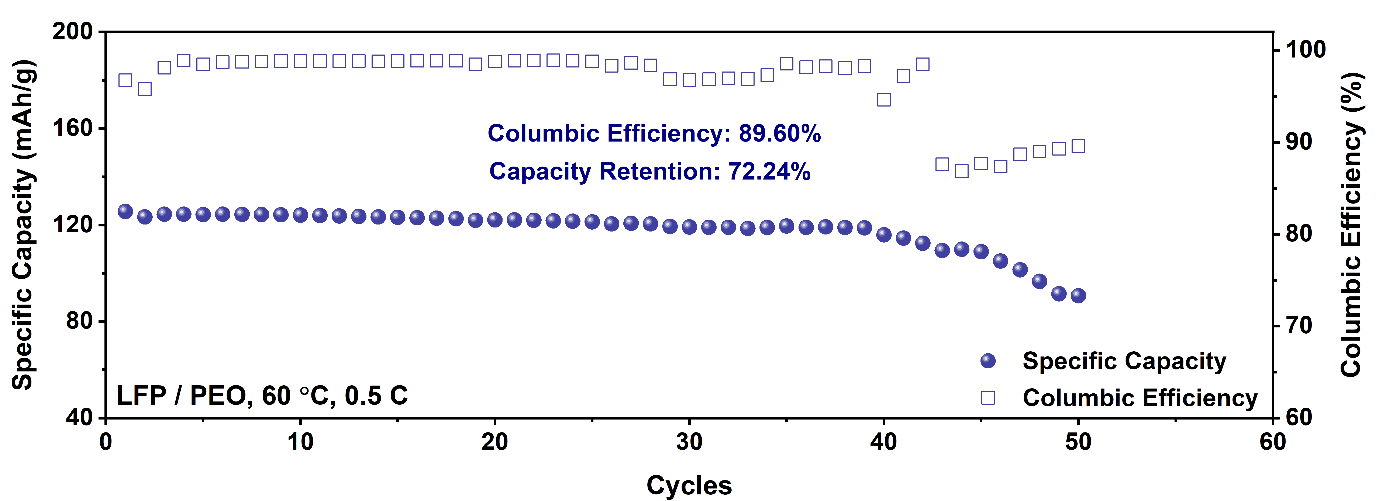


**Figure S38:** **Electrochemical performance of PEO flexible pouch cell.** LFP‖ PEO ‖Li pouch cell shows 72.24% capacity retention after 50 cycles, revealing pronounced capacity fading and limited long-term cycling stability.


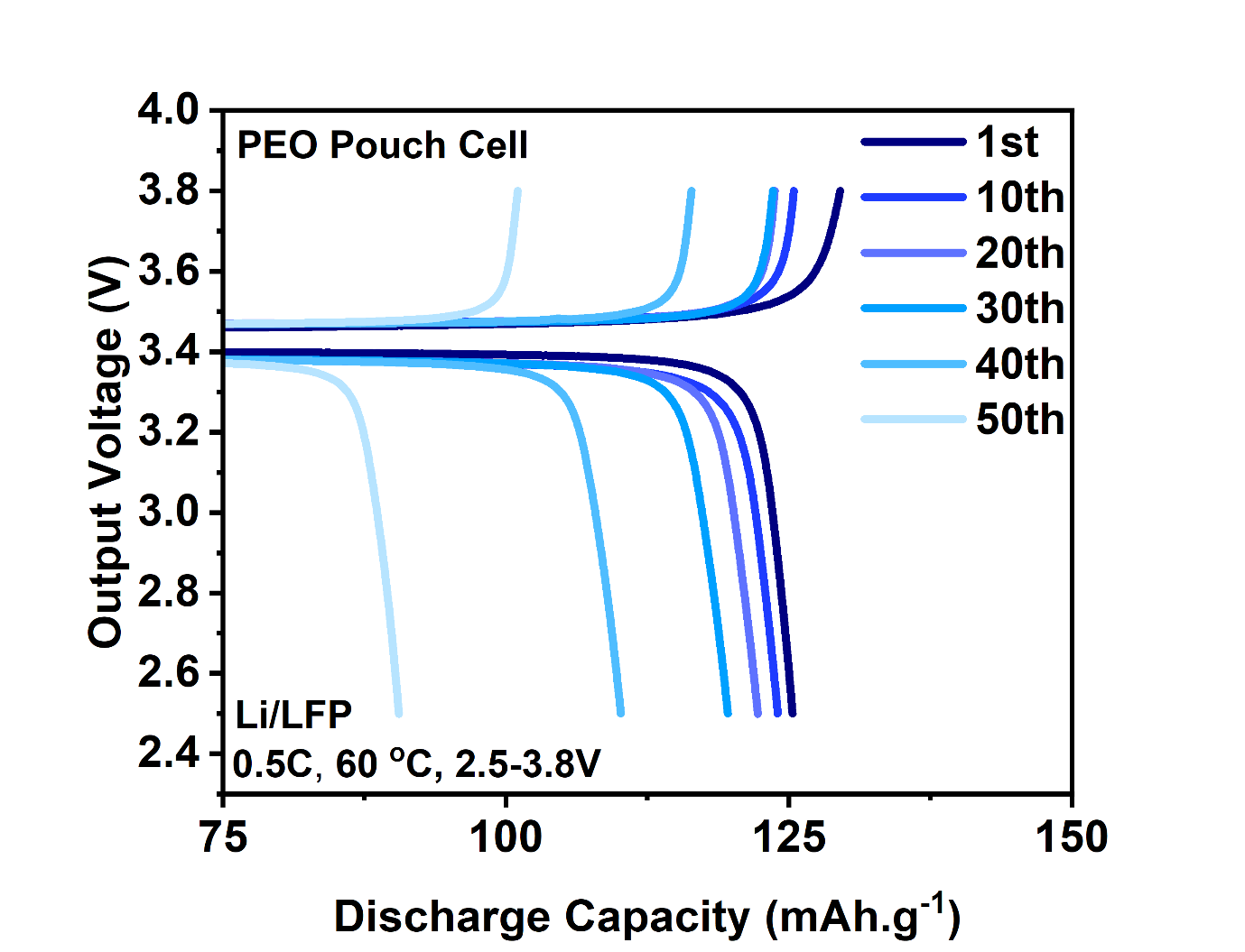


**Figure S39. Galvanostatic charge–discharge (GCD) profiles of PEO-based pouch cell (Li‖LFP) at 0.5 C and 60 °C (2.5–3.8 V).** The cell shows pronounced and continuous capacity decay with cycling, accompanied by increasing polarization, as evidenced by progressive shift and distortion of voltage profiles, ultimately leading to failure by the 50^th^ cycle.
